# Methodology and results of cost-effectiveness of LDL-C lowering with evolocumab in patients with acute myocardial infarction in China

**DOI:** 10.1186/s12962-023-00501-4

**Published:** 2023-12-01

**Authors:** Yuansheng Wan, Jinyu Liu, Xiaolian Zhan, Yu Zhang, Ruxu You

**Affiliations:** 1 Department of Pharmacy, Union Hospital, Tongji Medical College, Huazhong University of Science and Technology, 1277 Jiefang Avenue, Wuhan, 430022 Hubei China; 2 Department of Pharmacy, Tongji Hospital, Tongji Medical College, Huazhong University of Science and Technology, Wuhan, Hubei China

**Keywords:** Cost-effectiveness, Methodology, Evoloumab, Acute Myocardial Infarction

## Abstract

**Background:**

According to the Chinese guidelines for lipid management (2023), evolocumab in combination with statins was recommended as secondary prevention of cardiovascular disease. However, because of the variation in the price of evolocumab and its different methods of confirming clinical efficacy, it was necessary to explore its economics and the impact of different methods of confirming efficacy on its economic studies.

**Objective:**

The purpose of this paper was to assess the cost-effectiveness of evolocumab with statins versus statins alone for patients with acute myocardial infarction(AMI) in China and to investigate the impact of different clinical effectiveness modeling approaches on economic outcomes.

**Methods:**

A Markov cohort state-transition model was used to estimate the incremental cost-effectiveness ratio (ICER) based on Chinese observational data on cardiovascular event rates, efficacy from the Asian subgroup of the FOURIER trial, cost and utility from the Chinese Yearbook of Health Statistics, health insurance data, and published studies conducted in China. This study conducted subgroup analyses for different populations and dosing regimens; sensitivity analyses for parameters such as cost, utility, and cardiovascular event rates; and scenario analyses on hospital hierarchy, time horizon, starting age, and price for statins.

**Results:**

ICERs ranged from 27423 to 214777 Chinese yuan(CNY) per QALY gained, all below the willingness-to-pay threshold of CNY 257094. Only when the time horizon became small, the ICERs were greater than the willingness-to-pay. The probabilities that adding evolocumab to statins was cost-effective ranged from 76 to 98%. When the time horizon became small, i.e. evolocumab was discontinued before the age of 75 (after conversion), the corresponding ICERs were almost always greater than the willingness-to-pay. ICERs for modelling approaches based on clinical endpoints were 1.34 to 1.95 times higher than ICERs for modelling approaches based on reduced LDL-C levels.

**Conclusions:**

From the Chinese healthcare and private payer perspectives, adding evolocumab to statin therapy in AMI patients is more likely to be a cost-effective treatment option at the current list price of CNY 283.8. However, evolocumab may not be cost-effective if used for shorter periods of time. The results based on different clinical effectiveness modeling approaches were significantly different.

**Supplementary Information:**

The online version contains supplementary material available at 10.1186/s12962-023-00501-4.

## Introduction

Acute myocardial infarction (AMI) is the most severe cardiovascular disease (CVD) expression. Despite significant improvements in prognosis over the past decade, it remains one of the leading causes of morbidity and mortality worldwide, with more than 7 million people affected worldwide each year [1]. The morbidity and mortality of AMI in China showed an increasing trend year by year, causing a substantial economic burden. From 1980 to 2019, the average annual growth rate of AMI discharges in China was 10.94%, much higher than that of all disease discharges during the same period (6.33%). The total hospitalization cost for AMI in 2019 was Chinese yuan (CNY) 32 billion. The average annual growth rate of total inpatient costs for AMI since 2004 was 25.99%, much higher than that of cerebral infarction and cerebral hemorrhage (18.82% and 13.51%, respectively) [2, 3].

The association between lowering low-density lipoprotein cholesterol (LDL-C) levels and reducing the incidence of MI has been confirmed by several studies and is widely accepted [4, 5]. Conventional therapies for patients with elevated LDL cholesterol levels primarily involved oral medications such as statins and ezetimibe [6]. However, over 80% of high-risk patients do not meet the recommended LDL-C target. In recent years, inhibitors of the proprotein convertase subtilisin/kexin type 9 (PCSK9), including alirocumab and evolocumab, have been available for the treatment of patients with atherosclerotic cardiovascular disease (ASCVD) or familial hypercholesterolemia whose LDL-C levels remained elevated despite conventional therapy. Compared to conventional therapy alone, several large studies have demonstrated the superiority and safety of PCSK9 inhibitors combined with conventional therapy in terms of lipid-lowering and reduction of cardiovascular events [7–9]. Furthermore, PCSK9 inhibitors have been recommended by several guidelines and expert consensus for managing lipids [10–12].

Since the first PCSK9 inhibitor, evolocumab, appeared on the market in 2015, studies on the cost-effectiveness of PCSK9 inhibitors have continued to emerge [13]. Evolocumab entered the Chinese market in 2018 and was admitted to the National Medical Insurance Reimbursement List in 2022. Subsequently, the drug price was reduced from 1298 CNY/140 mg to 283.8 CNY/140 mg. To date, there have been three studies on the cost-effectiveness of evolocumab in China settings [14–16]. Two of the three studies were conducted before the drug price reduction [14, 15]. Only one study was performed based on the current price of 283.8 CNY/140 mg, which concluded that the probability of cost-effectiveness of adding evolocumab to statins was 100% [16]. However, this study left much to be desired; for example, most of the costs of disease treatment originated from a regional study and were much higher than the officially reported average, not to mention the large variations in the costs of disease treatment between different levels of hospitals; the effect on the results of changes in the price of statins used as a control (tens of times difference between the lowest and highest prices) is not mentioned; moreover, the health utility values used in this study were derived from a small sample survey in the UK [17] rather than from the Chinese population. In addition, as in previous studies, the treatment effect modeling approach considered only one of the two commonly used approaches (based on clinical endpoints [14] or based on reductions in LDL-C levels [15]), and the differences in outcomes produced by these two approaches have not been validated by studies. In view of the many issues mentioned above, further validation of the results of this study is therefore required.

This current study analyzed the cost-effectiveness of two dosing regimens (officially approved) of evolocumab from the perspectives of Chinese healthcare and private payers, respectively. The study analyzed the populations at different baseline levels used in previous economics studies based on officially reported disease treatment costs and health utility values of the Chinese population, respectively, and further discussed the impact of many factors on the results, such as the significant disparities in treatment costs between different levels of hospitals in China. In addition, the treatment effect modeling approach in this study considered both clinical endpoint-based and LDL-C level reduction-based approaches and validated the impact of these two approaches on outcomes. The results of this current study will provide public policymakers, health insurance providers, and private payers with as detailed a reference as possible when choosing a more economical strategy.

## Methods

The study was reported in line with the Consolidated Health Economic Evaluation Reporting Standards 2022 (CHEERS 2022) [18] (Supplementary Table S1).

### Model structure

**Fig. 1:**
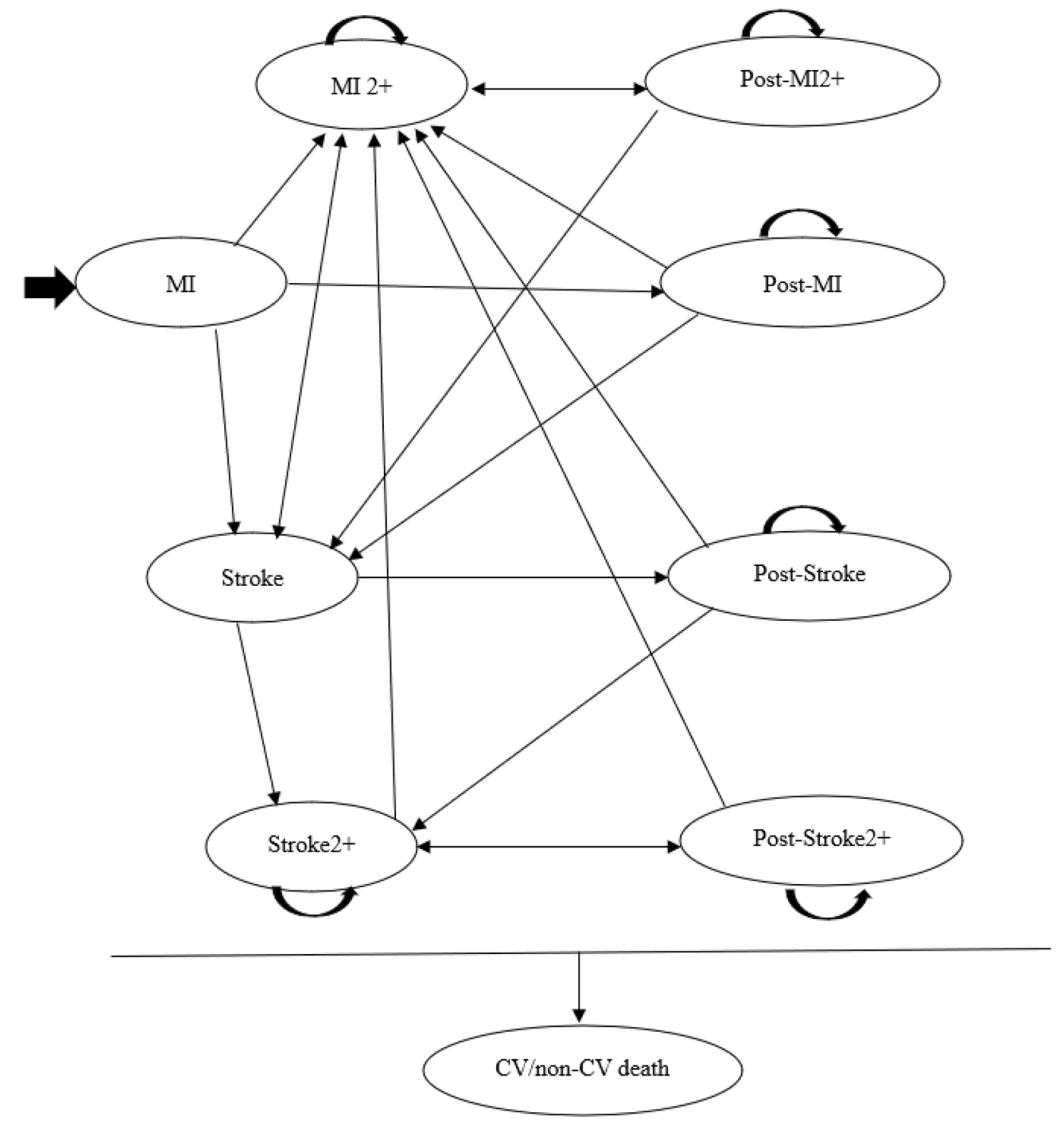
Markov cohort state-transition model Diagram CV, cardiovascular; MI, myocardial infarction

A Markov cohort state-transition model was employed to evaluate the cost-effectiveness of evolocumab and statins versus statins, which considered the perspectives of the Chinese healthcare and private payer and assumed lifetime horizon to model the lifetime progression of patients with AMI. The model (Fig. 1) was refined from the most recently published models [15, 16, 19] and was utilized to project subsequent cardiovascular events according to LDL-C levels, age, and history of cardiovascular events [15, 16, 19, 20] (Supplementary Table S2). The model consisted of ten primary and mutually exclusive health states, including four acute event states (Non-fatal MI and Non-fatal stroke for a 1-year duration after the first event, Non-fatal MI2 + and Non-fatal stroke2 + for the one year after two or more sequential MI or stroke), post-event health states, their composite health states, and cardiovascular (MI and stroke) or non-cardiovascular related death. These combined health states were a combination of two health states that preserved the memory of previous CV events and better reflected the incremental risk, decreased quality of life, and increased costs related to multiple CV event experiences. Thus, the utility value of the combined health state was assumed to be the lowest utility of all the individual health states that comprised the combined state; the cost of the combined health state was assumed to be the highest cost of the individual health states. In addition, as in previously published models [19, 20], revascularization (RV) was considered a procedure (i.e., cost) rather than an independent health state. The half-cycle correction was applied to all events in the model. The model was developed using TreeAge Pro software (Williamstown, MA, USA).

### Population

Real-world data were employed in this study to model the AMI population in clinical practice that reflected the representative characteristics of the Chinese MI patient population. Patients were extracted separately from three recently published, clinical practice-based studies, i.e., China Patient-Centered Evaluative Assessment of Cardiac Events Prospective Study of AMI (China PEACE-Prospective AMI Study) [21], the standardized Chinese hospital-based health information system electronic database (SuValue®) (containing two subgroups: LDL-C ≥ 100 mg/dl [15] or LDL-C ≥ 70 mg/dl [16]), and the Chinese population from the BERSON clinical trial (evolocumaB Efficacy study for LDL-C Reduction in subjectS with T_2_DM On background statiN) [22]. Detailed information is shown in the Table S3 and Supplementary material.

### Mortality

In each cycle after the first cycle, cardiovascular mortality rates varied per age-related changes in China-specific mortality records (Health Statistics Yearbook, 2022 edition [2]). Non-cardiovascular mortality was calculated by subtracting cardiovascular mortality from all-cause mortality to capture the natural mortality of the Chinese people. The all-cause mortality rates for different age groups were derived from the Chinese census [23].

### Treatment effects

Treatment effects in the model were derived from the analysis of clinical endpoints of various cardiovascular events in the FOURIER trial [7] and the association between LDL-C lowering and the reduction of cardiovascular event rates (Results of meta-analyses performed by the Cholesterol Treatment Trialists Collaborative (CTTC)) [4, 5], as previously reported [16, 20]. Detailed information is shown in Table 1.

**Table 1:** Key Inputs in the Model

Parameter	Value	Range	Distribution	Source
**Event rate per 100 patient-years, PEACE population with AMI**				
Nonfatal MI	1.7	NA	NA	[21]
Nonfatal stroke	0.9	NA	NA	
Cardiovascular-related death	2.2	NA	NA	
All-cause mortality	3.1	NA	NA	
Coronary revascularization	6.4	NA	NA	
**Event rate per 100 patient-years, trial population of FOURIER trial**				
Nonfatal MI	2.5	2.2–2.7	Lognormal	[7]
Nonfatal stroke	0.9	0.8–1.1	Lognormal	
Cardiovascular-related death	0.8	0.7–1.0	Lognormal	
Coronary revascularization	3.9	3.6–4.3	Lognormal	
**Intervention effect, hazard ratio**				
Nonfatal MI (year 1)	0.79	0.67–0.93	Lognormal	[7, 24]
Nonfatal MI (beyond 1y)	0.64	0.54–0.76	Lognormal	
Nonfatal stroke (year 1)	0.52	0.29–0.92	Lognormal	
Nonfatal stroke (beyond 1y)	0.52	0.29–0.92	Lognormal	
Coronary revascularization (year 1)	0.84	0.74–0.96	Lognormal	
Coronary revascularization (beyond 1y)	0.72	0.63–0.82	Lognormal	
**Intervention effect, rate ratio per 1mmol/l LDL-C reduction**				
Nonfatal MI (year 1)	0.84	0.76–0.92	Lognormal	[4]
Nonfatal MI (beyond 1y)	0.74	0.70–0.78	Lognormal	
Nonfatal stroke (year 1)	0.96	0.82–1.12	Lognormal	
Nonfatal stroke (beyond 1y)	0.81	0.74–0.88	Lognormal	
Coronary revascularization (year 1)	0.88	0.80–0.97	Lognormal	
Coronary revascularization (beyond 1y)	0.71	0.67–0.75	Lognormal	
Vascular causes of death	0.86	0·82 − 0·90	Lognormal	
**Annual cost of drugs, CNY**				
Evolocumab (140 mg/2W)	7405	NA	NA	[28]
Evolocumab (420 mg/M)	10365	NA	NA	[28]
Statins	2855	2141.25–3568.75	Gamma	[28]
**Cost of cardiovascular events, CNY**				
**Direct cost**				
Nonfatal MI (year 1)	26518.90	19889.18–33148.63	Gamma	[2]
Nonfatal MI (beyond 1y)	13377.54	10033.15–16721.92	Gamma	[25]
Nonfatal stroke (year 1)	12634.51	9475.88–15793.14	Gamma	[2]
Nonfatal stroke (beyond 1y)	10141.98	7606.49–12677.48	Gamma	[27]
Coronary revascularization	116279.90	87209.93–145349.88	Gamma	[2]
Stroke + Post MI	13637.40	10228.05–17046.76	Gamma	[37]
Death due to stroke	14063.87	10547.9–17579.83	Gamma	[27, 38]
Death due to MI	22687.86	17015.89–28359.82	Gamma	[26, 39–41]
**Indirect cost**				
Nonfatal MI (year 1)	2037.44	1528.08–2546.8	Gamma	[2]
Nonfatal stroke (year 1)	2629.56	1972.17–3286.95	Gamma	[2]
Nonfatal stroke (beyond 1y)	13312.44	9984.33–16640.55	Gamma	[29]
Coronary revascularization	5282.26	3961.69–6602.82	Gamma	[2]
Stroke + Post MI	2766.90	2075.17–3458.62	Gamma	[37]
Death due to stroke	3395.74	2546.81–4244.68	Gamma	[42]
Death due to MI	2489.37	1867.03–3111.71	Gamma	[26, 39–41]
**Utility**				
Nonfatal MI (year 1)	0.866	0.847–0.886	Beta	[21, 33, 43]
Nonfatal MI (beyond 1y)	0.950	0.942–0.958	Beta	[32, 33, 43]
MI 2+(year 1)	0.819	0.793–0.846	Beta	[33, 43]
MI 2+ (beyond 1y)	0.940	0.905–0.975	Beta	[32, 33, 43]
Nonfatal stroke (year 1)	0.510	0.470–0.540	Beta	[34]
Nonfatal stroke (beyond 1y)	0.750	0.710–0.800	Beta	[34, 44]
Stroke 2+ (year 1)	0.340	0.320–0.360	Beta	[35]
Stroke 2+ (beyond 1y)	0.420	0.390–0.451	Beta	[35]
Injection site reaction, disutility	-0.0003	-0.002–0	Beta	[20]

AMI, Acute myocardial infarction; CNY, Chinese Yuan; LDL-C, low-density lipoprotein cholesterol; MI, myocardial infarction; NA, not applicable;

For the PEACE study population, the assumed treatment effects were based on the hazard ratios(HRs) for MI, stroke, and coronary revascularization in the FOURIER trial (with rates of 79%, 83%, and 84% in the first year and 64%, 76%, and 72% beyond year 1, respectively) [7, 20]. For 2723 Asian patients (including 1165 Chinese) in the FOURIER trial, risk reductions for MI and coronary revascularization were similar among Asian patients and other patients, but the risk reduction for stroke was more significant in the Asian population (HR: 0.52) than in other populations (HR: 0.82) [24]. Therefore, the HRs for MI, stroke, and coronary revascularization in the PEACE study population were assumed to be 79%, 52%, and 84% in the first year and 64%, 52%, and 72% after the first year, respectively. In addition, since only deaths due to MI and stroke were considered in this model, only the mortality associated with MI and stroke were extracted from the results of the FOURIER trial and combined into cardiovascular mortality to obtain the corresponding HRs.

For the SuValue® database population, the assumed treatment effects were based on the association between LDL-C lowering and the reduction of cardiovascular event rates identified in CTTC meta-analyses. In the FOURIER trial, at 48 weeks, compared with placebo (high-intensity statin), the mean percentage decrease in LDL-C levels was higher in Asians with evolocumab than in others (66% vs. 58%; *P* < 0.001) [24]. Moreover, the effectiveness of evolocumab has been shown to last up to 5 years (the longest follow-up to date) by the results of the OSLER-1 trial [8]. Therefore, it was assumed that the 66% reduction in mean LDL-C with evolocumab compared with placebo would last for the lifetime of treatment. As previously reported [16, 19], the event-specific ratios employed in this model were derived from the CTTC meta-analysis results, which demonstrated that the rate ratio (RR) for MI, stroke, coronary revascularization, and CV death per 38.7 mg/dl (1.0 mmol/l) of LDL-C reduction were 84%, 96%, 88%, and 86% in the first year and 74%, 81%, 71%, and 86% after the first year, respectively [4, 5]. The incidences of CV events after treatment were measured by the following equation:

$${r_{tx}}\, = \,{r_0}\, \times \,R{R^{\Delta LDL - C}}$$

where r_tx_, rate after treatment; r_0_, rate before treatment; RR, rate ratio per 38.7 mg/dl (1.0 mmol/l) of LDL-C reduction; ΔLDL-C, the absolute reduction of LDL-C.

For Chinses patients in the BERSON trial, evolocumab treatment reduced LDL-C by 72.8 mg/dl (1.88 mmol/l) for the 140 mg Q2W dose and 65.4 mg/dl (1.69 mmol/l) for the 420 mg QM dose at week 12 compared to the placebo (atorvastatin 20 mg/d) [22]. As previously described, the treatment effect of evolocumab was assumed to be maintained over a lifetime. Moreover, the treatment effect was calculated approximately in the same way for the SuValue® database population.

For drug safety in the FOURIER trial and the BERSON study, no significant differences were found between evolocumab and placebo in the total rate of adverse events and serious adverse events. However, injection site reactions were more likely to occur when evolocumab was used (*P* < 0.001). Therefore, adverse drug reactions in this model included only mild injection site reactions: 2.1% of individuals experienced mild injection site reactions within 26 months [24]. The effects of persistence and compliance on efficacy have been covered by the results of the FOURIER trials and were therefore not replicated in this model.

### Costs

In this model, costs related to cardiovascular events considered separately for direct and indirect costs (Table 1). Direct costs incorporated direct medical costs such as medications and hospitalization but excluded direct non-medical costs such as transportation and nutrition costs. Hospitalization costs were mainly from the officially published Health Statistics Yearbook (2022 edition) [2]. Post-hospital long-term follow-up costs were obtained from previous studies based on basic medical insurance [25, 26] and the China Stroke Big Data Observatory platform [27], respectively. Furthermore, as previously reported [16, 20], non-CVD deaths were assumed to be costless and did not differ by treatment option. Medication costs were estimated mainly based on the national centralized procurement prices or winning bids for each drug published on the China Pharmcube website in February 2023 [28]. The price of statins was calculated by weighting the median price of low-, medium-, and high-intensity statins by the proportion in the FOURIER trial. Details are provided in Supplementary Table S4. The price of evolocumab (140 mg) was CNY 283.8. The annual cost of evolocumab for 140 mg Q2W was CNY 7405, while the annual cost for 420 mg QM was CNY 10365. Indirect costs included lost wages and informal family care. Since the study subjects in this model were the retired population (age over 60), the lost wages were the costs or lost wages of the caregiver during the hospitalization of the patient rather than the patient’s wage loss. Costs for informal home care after hospital discharge were mainly from previous reports [29] and estimated from data from the CNSR.

Costs were presented in US dollars and Chinese Yuan based on the 2022 average market exchange rate (US$1 = CNY 6.726) [30]. All cost data were inflated to 2022 values with the Consumer Price Index (CPI) for Health Care [31].

### Utility

Utility scores (ranging from 0 (death) to 1 (perfect health)) were obtained from the Chinese PEACE-Prospective AMI Study [21] and other studies of utility values in the Chinese population [32–35]. In these studies, utility values were measured using the Chinese version of EuroQoL five-dimension three-level scales (EQ-5D-3L), and the calculation formula was derived from the Chinese-specific scoring algorithm of EQ-5D. Utility scores of each health state are presented in Table 1.

### Base case cost-effectiveness analysis (CEA)

Costs and quality-adjusted life years (QALYs) were developed from the model for two treatment regimens (evolocumab with statins vs. statins alone). The differences in costs and QALYs between the two treatment regimens were the incremental costs and incremental QALYs. The ratio of incremental costs to incremental QALYs was the cost-effectiveness, usually stated as incremental cost-effectiveness ratios (ICERs). According to the recommendations of China guidelines for pharmacoeconomic evaluations (2020) [36], the annual discount rates for costs and utilities were 5%, and the willingness-to-pay (WTP) threshold for QALY was three times the gross domestic product (GDP) per capita.

A treatment strategy was regarded as “highly cost-effective” if the ICER was less than GDP per capita; “cost-effective” if the ICER was between one and three times the GDP per capita; otherwise, this strategy was regarded as “not cost-effective”. China’s GDP per capita in 2022 was CNY 85698 (US$12741) [31], and the WTP was set at CNY 257094 (US$38224). The study time horizon of the model was assumed to be 25 years (covering the majority of the Chinese population).

### Sensitivity analyses

Sensitivity analyses, including deterministic (DSA) and probabilistic sensitivity analyses (PSA), were performed to assess the impact of reasonable changes in model parameters (including utility scores, costs, and discount rates) on the robustness of the results. DSA was performed with univariate sensitivity analysis and the results were presented in a tornado diagram. The efficacy parameters, utility values, baseline rates, and adjustment factors varied within their 95%CI. The event costs varied between ± 25% of the baseline value. The discount rate was assigned to change between 0 and 8% (Table 1). PSA was conducted using Monte Carlo simulations (10000 replications) with the results displayed as cost-effectiveness acceptability curves and Monte Carlo simulation scatter plots. The distribution of each model parameter is presented in Table 1.

### Scenario analyses

Scenario analyses were conducted separately for time horizon, age of starting treatment, discount rate, maximum and minimum centralized purchasing prices or winning prices for statins, and costs, utility values, and HRs used in other CEA studies that were beyond the scope of the sensitivity analyses. The analysis was also performed for the cost of disease treatment at different levels of hospitals based on the Health Statistics Yearbook. More detailed parameters are shown in Supplementary Tables S5, S6, and S7.

### Model validation

The model was improved from an existing model, peer-reviewed, and cross-checked by clinicians to make the model broadly consistent with the actual course of the disease, ensuring the face validity and cross-validity of the model. In addition, calculations at each step were made as accurately as possible to ensure the technical validity of the model.

### Comparison of different modeling treatment effect methodologies

Based on the FOURIER trial population, the treatment effects of evolocumab were modeled utilizing the two most popular methodologies described above, i.e., clinical outcomes and LDL-C reduction levels from the FOURIER trial, respectively. The corresponding ICER values were then calculated and the scenario analysis described above was performed.

## Results

### Base-case analyses

From the Chinese healthcare perspective, in these base cases using the PEACE study population, the SuValue® database population with LDL-C ≥ 100 mg/dl or LDL-C ≥ 70 mg/dl, and the BERSON trial population, respectively, the ICERs with evolocumab 140 mg Q2W compared to statins were 137755.18, 40905.80, 72449.95, and 94869.54 CNY per QALY gained; likewise, the ICERs with evolocumab 420 mg QM were 214777.76, 90214.47, 133390.36, and 177450.90 CNY per QALY gained, in that order (Table 2).

**Table 2:** Base-case cost-effectiveness results from the Chinese healthcare perspective

	Cost, CNY		QALY		ICER, CNY
	Total	Incremental	Total	Incremental	
PEACE					
Statins	299509.17	NA	8.19	NA	NA
Statins + Evo 140mgQ2W	348242.12	48732.96	8.54	0.35	137755.18
Statins + Evo 420mgQM	375490.01	75980.84	8.54	0.35	214777.76
BERSON^a^					
Statins	279123.88	NA	9.64	NA	NA
Statins + Evo 140mgQ2W	330152.91	51029.03	10.18	0.54	94869.54
Statins + Evo 420mgQM	365284.28	86160.40	10.13	0.49	177450.90
SuValue®, LDL-C ≥ 100 mg/dl					
Statins	300258.69	NA	8.33	NA	NA
Statins + Evo 140mgQ2W	324484.37	24225.68	8.92	0.59	40905.80
Statins + Evo 420mgQM	353686.48	53427.79	8.92	0.59	90214.47
SuValue®, LDL-C ≥ 70 mg/dl					
Statins	284482.16	NA	8.39	NA	NA
Statins + Evo 140mgQ2W	318921.61	34439.45	8.86	0.48	72449.95
Statins + Evo 420mgQM	347889.94	63407.78	8.86	0.48	133390.36

CNY, Chinese Yuan; Evo 140mgQ2W, evolocumab 140 mg every 2 weeks; Evo 420mgQM, evolocumab 420 mg monthly; LDL-C, low-density lipoprotein cholesterol; MI, myocardial infarction; NA, not applicable

a: The background statin in the BERSON study was atorvastatin 20 mg/d

From the Chinese private payer perspective, in these base cases using the PEACE study population, the SuValue® database population with LDL-C ≥ 100 mg/dl, the SuValue® database population with LDL-C ≥ 70 mg/dl, and the BERSON trial population, respectively, the ICERs with evolocumab 140 mg Q2W compared to statins were 121986.67, 27423.06, 58806.29, and 83078.56 CNY per QALY gained, in that order; likewise, the ICERs with evolocumab 420 mg QM were 199009.25, 76731.73, 119746.70, and 165565.29 CNY per QALY gained, in that order (Table 3).

**Table 3:** Base-case cost-effectiveness results from the Chinese private payers perspective

	Cost, CNY		QALY		ICER, CNY
	Total	Incremental	Total	Incremental	
PEACE					
Statins	324863.85	NA	8.19	NA	NA
Statins + Evo 140mgQ2W	368018.46	43154.61	8.54	0.35	121986.67
Statins + Evo 420mgQM	395266.35	70402.50	8.54	0.35	199009.25
BERSON^a^					
Statins	306842.49	NA	9.64	NA	NA
Statins + Evo 140mgQ2W	351529.31	44686.82	10.18	0.54	83078.56
Statins + Evo 420mgQM	387231.89	80389.40	10.13	0.49	165565.29
SuValue®, LDL-C ≥ 100 mg/dl					
Statins	330228.73	NA	8.33	NA	NA
Statins + Evo 140mgQ2W	346469.52	16240.78	8.92	0.59	27423.06
Statins + Evo 420mgQM	375671.63	45442.90	8.92	0.59	76731.73
SuValue®, LDL-C ≥ 70 mg/dl					
Statins	312147.77	NA	8.39	NA	NA
Statins + Evo 140mgQ2W	340101.64	27953.87	8.86	0.48	58806.29
Statins + Evo 420mgQM	369069.97	56922.20	8.86	0.48	119746.70

CNY, Chinese Yuan; Evo 140mgQ2W, evolocumab 140 mg every 2 weeks; Evo 420mgQM, evolocumab 420 mg monthly; LDL-C, low-density lipoprotein cholesterol; MI, myocardial infarction; NA, not applicable

a: The background statin in the BERSON study was atorvastatin 20 mg/d

All of the above ICERs were less than the WTP (CNY 257094), indicating that both the evolocumab 140 mg Q2W regimen and the evolocumab 420 mg QM regimen were cost-effective compared to statins alone. In addition, ICERs with evolocumab 140 mg Q2W and evolocumab 420 mg QM were lower than GDP per capita (CNY 85698) in the SuValue® database population with LDL-C ≥ 70 mg/dl or LDL-C ≥ 100 mg/dl, indicating that both regimens were highly cost-effective.

### Sensitivity analyses

**Fig. 2:**
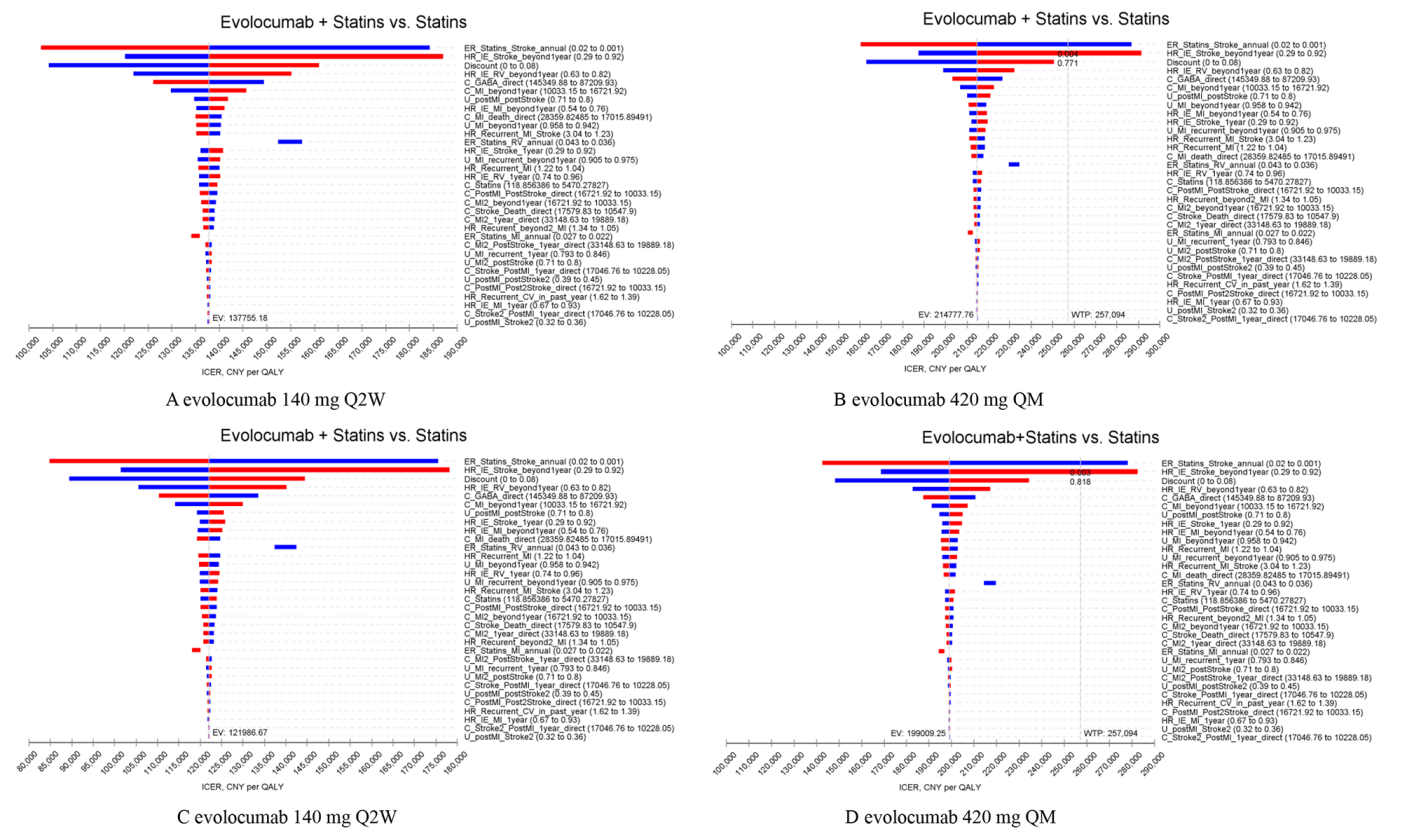
Tornado plots for one-way deterministic sensitivity analysis of the PEACE study population. (**A** and **B**) are the results from the Chinese healthcare perspective, C and D are the results from the Chinese private payer perspective The dotted line shows the willingness-to-pay threshold of CNY 257094 per quality-adjusted life-year gained; C_, Cost; CNY, Chinese yuan, CV, cardiovascular; ER_, event rate; EV, expected value; HR_, Hazard ratio; ICER, incremental cost-effectiveness ratio; IE_, Intervention effect; MI, myocardial infarction; RV_, revascularization; U_, Utility; WTP, willingness-to-pay

DSA showed that almost all of the above base case results were robust to changes of parameters in the model, except for the results for ICERs using evolocumab 420 mg QM in the PEACE study population from both perspectives (Fig. 2, and Supplementary Figures S2, S3, and S4). These parameters that had a greater impact on the base-case outcomes were the discount rate, the HRs for patients with a history of stroke or RV, the cost of post-discharge for patients with MI, and the cost of hospitalization for GABA, respectively. Uncertainty in the HR for patients with a history of stroke resulted in a large change in the ICER for evolocumab 420 mg QM under both perspectives, from 214777.76 to 199009.25 CNY per QALY gained (cost-effective) to not cost-effective, respectively; Similarly, the uncertainty in the event rate of stroke after statin therapy alone led to the same results (Fig. 2).

**Fig. 3:**
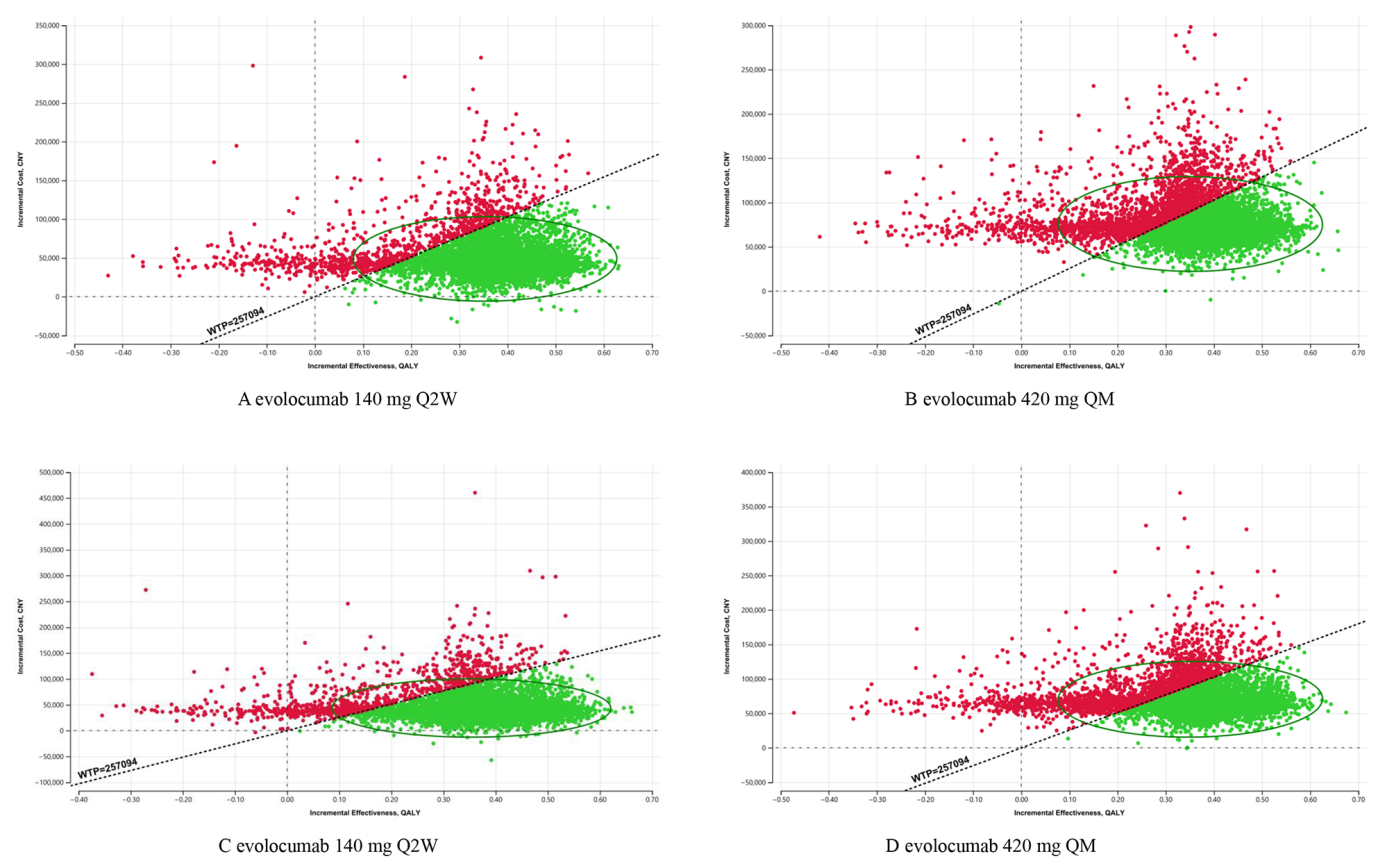
Monte Carlo simulation scatters plot in probabilistic sensitivity analyses of the PEACE study population. (**A** and **B**) are the results from the Chinese healthcare perspective, C and D are the results from the Chinese private payer perspective The dotted line shows the willingness-to-pay threshold, with a slope of CNY 257094 per quality-adjusted life-year gained. CNY, Chinese yuan; QALY, quality-adjusted life-year; WTP, willingness-to-pay

**Fig. 4:**
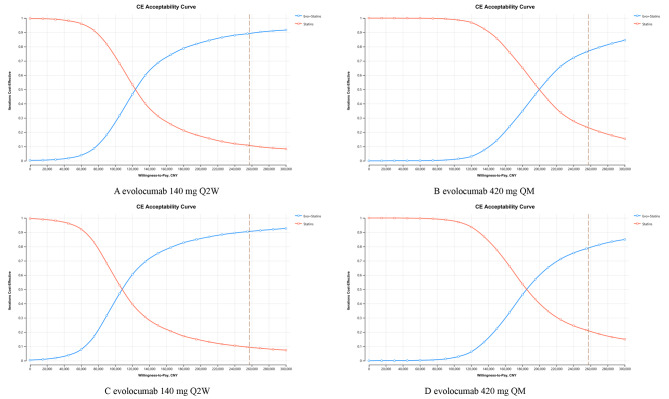
Cost-effectiveness acceptability curves in probabilistic sensitivity analyses of the PEACE study population. (**A** and **B**) are the results from the Chinese healthcare perspective, C and D are the results from the Chinese private payer perspective The dashed line shows the willingness-to-pay threshold of CNY 257094 per quality-adjusted life-year gained. CNY, Chinese yuan; Evo, evolocumab; QALY, quality-adjusted life-year

The results of the PSA were presented in Figs. 3 and 4, and Figures S4 to S9 in the Supplement. For the PEACE study population, the SuValue® database population with LDL-C ≥ 100 mg/dl or ≥ 70 mg/dl, and the BERSON trial population, from a Chinese healthcare perspective, at the list price of CNY283.8/140 mg, the probabilities that evolocumab 140 mg Q2W added to statins is cost-effective at the generally accepted WTP of CNY 257,094 per QALY gained were 89.19%, 95.61%, 93.93%, and 92.45%, respectively; Similarly, the probabilities for evolocumab 420 mg QM were 76.91%, 92.51%, 88.94%, and 91.07% respectively. From a Chinese private payer perspective, and in the same setting described above, the probabilities of evolocumab 140 mg Q2W were 90.39%, 96.45%, 94.87%, and 98.10%, respectively, while the probabilities of evolocumab 420 mg QM were 79.29%, 93.66%, 90.43%, and 86.63% respectively.

### Scenario analyses

Scenario analyses examined nearly 30 alternative scenarios of the treatment benefit. The results of scenario analyses showed that the base case results, i.e., ICERs below three times GDP per capita, were robust in the majority of scenarios. Only when the time horizon became smaller did the ICER values change significantly, i.e., when the study ended before age 75, the corresponding ICER values were almost always higher than the WTP values. Detailed information is shown in Tables 4 and 5.

**Table 4:** ICERs of scenario analyses from the Chinese healthcare perspective (CNY per QALY gained)

	PEACE		BERSON		SuValue® LDL-C ≥ 100 mg/dl		SuValue® LDL-C ≥ 70 mg/dl	
	Evo 140mgQ2W	Evo 420mgQM	Evo 140mgQ2W	Evo 420mgQM	Evo 140mgQ2W	Evo 420mgQM	Evo 140mgQ2W	Evo 420mgQM
**Time horizon**								
5 years	**560431.63**	**862489.78**	**501332.97**	**893539.69**	195951.91	242966.70	**322809.94**	**557316.71**
10 years	**284569.36**	**440560.92**	242330.14	**439597.72**	94332.72	194182.08	160883.45	**285355.56**
15 years	191287.82	**296835.37**	154973.97	**284122.89**	*62166.54*	131380.50	107590.05	193482.78
20 years	152155.45	236588.60	114428.44	211855.12	*47781.40*	103208.66	*83668.90*	152268.70
30 years	134240.70	209956.59	86535.32	163514.29	37302.63	84166.61	*67030.66*	124934.42
35 years	133083.42	208719.66	*83376.17*	158919.09	*NA*	NA	*NA*	NA
**Starting age**								
65-year	121365.52	172717.45	*73383.57*	138037.76	*NA*	NA	*NA*	NA
70-year	103267.71	150351.27	*56517.58*	108060.03	*39605.72*	87392.58	*70034.35*	129059.14
75-year	*84946.74*	126891.51	*42483.76*	*83985.21*	*30553.67*	*68195.31*	*53678.13*	99970.03
**Discount rate**								
3%	123519.88	192790.91	*83849.77*	157503.59	36035.97	80462.88	*64207.24*	119040.93
6%	145241.79	226352.26	100806.54	188192.28	*43473.21*	95365.52	*76801.17*	140974.87
**Annual Cost of Stains (CNY)** ^**a**^								
118.86	131102.46	212813.67	93705.52	176282.05	*38710.07*	88018.74	*70251.67*	131192.08
5470.28	134976.64	216654.87	95526.50	178110.59	*43004.30*	92312.97	*74550.88*	135491.29
**Costs of CV-related death** ^b^								
Death due to stroke 1	116570.06	193592.63	*73068.07*	155525.57	*16745.35*	*66054.01*	*48025.65*	108966.06
Death due to stroke 2	130382.94	207405.52	87351.56	169888.68	*32541.32*	*81849.98*	*63999.21*	124939.62
Death due to MI 1	118319.42	195341.99	*68158.82*	150384.52	*6528.07*	*55836.73*	*39090.98*	100031.39
Death due to MI 2	92301.26	169323.84	*35964.22*	117891.62	Dominance	*17416.88*	*1113.88*	*62054.29*
**Utility** ^c^								
Higher utility	156999.82	244782.59	103011.41	193226.79	*45005.11*	99255.17	*80019.62*	147327.16
Lower utility	126564.51	197330.08	89484.04	166987.79	38284.51	84433.42	*67616.20*	124490.76
UK- utility	146062.51	227729.93	100800.52	187476.68	*42461.83*	93646.16	*74998.51*	138082.62
**Hospital classification**								
County hospitals	154811.12	231833.69	113682.98	196641.29	*67932.09*	117240.76	98423.88	159364.30
County-level municipal hospitals	150510.37	227532.95	108608.40	191463.97	*60612.92*	109921.59	91418.36	152358.77
Prefecture-level municipal hospitals	136460.69	213483.27	93365.00	175916.39	*38748.53*	88057.20	*70387.91*	131328.32
Provincial hospitals	134431.86	211454.44	90173.78	172658.18	*34080.28*	*83388.94*	*65984.28*	126924.69
Hospitals directly under the Health Commission	129164.77	206187.35	*84385.48*	166754.78	*25788.08*	*75096.74*	*58021.98*	118962.39

CNY, Chinese Yuan; Dominance, ICERs, incremental cost-effectiveness ratio; Lower cost, higher utility; LDL-C, low-density lipoprotein cholesterol; MI, myocardial infarction; NA, not applicable

italic text: ICERs below CNY 85698 (US$12741); bold text: ICERs above CNY 257094 (US$38224).

a: the maximum and minimum annual cost of statins, more detailed parameters are shown in Supplementary Tables S4

b: CV-related mortality costs used in other CEA studies and beyond the scope of sensitivity analysis, more detailed parameters are shown in Supplementary Tables S5

c: Utility used in other CEA studies and beyond the scope of sensitivity analysis, more detailed parameters are shown in Supplementary Tables S6

**Table 5:** ICERs of scenario analyses from the Chinese private payer perspective (CNY per QALY gained)

	PEACE		BERSON		SuValue® LDL-C ≥ 100 mg/dl		SuValue® LDL-C ≥ 70 mg/dl	
	Evo 140mgQ2W	Evo 420mgQM	Evo 140mgQ2W	Evo 420mgQM	Evo 140mgQ2W	Evo 420mgQM	Evo 140mgQ2W	Evo 420mgQM
**Time horizon**								
5 years	**538578.65**	**840636.79**	**481061.72**	**873164.78**	174043.04	**360901.94**	**301236.99**	**535743.77**
10 years	**264847.73**	**420839.28**	225087.72	**422267.59**	*75562.59*	175411.94	142255.13	**266727.23**
15 years	173410.07	**278957.63**	139891.58	**268955.17**	*45586.22*	114800.18	91036.01	176928.74
20 years	135696.37	220129.52	101220.39	198558.46	*33040.08*	88467.34	*68849.69*	137449.49
30 years	118511.82	194227.70	*75503.67*	152378.28	*24439.97*	*71303.95*	*53946.33*	111850.08
35 years	117217.93	192854.18	*72506.95*	147936.29	NA	NA	NA	NA
**Starting age**								
65-year	108771.20	177305.36	*64624.43*	129223.90	NA	NA	NA	NA
70-year	93050.81	152702.01	*49543.94*	101051.16	*26725.74*	*74512.59*	*56998.67*	116023.46
75-year	*76221.16*	127484.02	36443.19	*77920.22*	*20824.50*	*58466.14*	*43803.33*	90095.23
**Discount rate**								
3%	108174.04	177445.07	*72565.94*	146128.62	*23150.27*	*67577.18*	*51123.46*	105957.15
6%	129263.01	210373.48	88758.57	176048.14	*29692.79*	*81585.10*	*62878.15*	127051.85
**Annual Cost of Stains (CNY)** ^a^								
118.86	120022.58	197045.16	*81914.54*	164396.45	*25227.33*	*74536.00*	*56608.02*	117548.43
5470.28	123863.78	200886.36	*83735.51*	166224.98	*29521.56*	*78830.23*	*60907.22*	121847.64
**Costs of CV-related death** ^b^								
Death due to stroke 1	100522.74	177545.32	*61045.06*	143405.39	*2979.10*	*52287.76*	*34099.45*	95039.86
Death due to stroke 2	114482.41	191504.99	*75468.74*	157909.73	*18935.65*	*68244.32*	*50234.53*	111174.94
Death due to MI 1	94656.25	171678.83	*55830.30*	137955.69	Dominance	*41690.59*	*24793.53*	85733.94
Death due to MI 2	*69467.93*	146490.51	*24696.42*	106532.67	Dominance	*4528.46*	Dominance	*49006.85*
**Utility** ^b^								
Higher utility	130475.81	218258.57	90208.50	180284.52	*30171.23*	*84421.29*	*64950.45*	132258.00
Lower utility	105182.33	175947.90	*78362.40*	155803.00	*25665.76*	*71814.68*	*54882.83*	111757.38
UK- utility	119895.54	200560.01	88272.39	174919.55	*28466.22*	*79650.55*	*60874.92*	123959.02
**Hospital classification** ^c^								
County hospitals	139220.80	216243.38	102079.64	184879.97	*54709.26*	104017.93	*85032.51*	145972.92
County-level municipal hospitals	134508.03	211530.61	96579.00	179335.09	*46786.37*	96095.03	*77442.66*	138383.07
Prefecture-level municipal hospitals	120332.05	197354.62	*81196.28*	163645.82	*24721.08*	*74029.74*	*56226.15*	117166.56
Provincial hospitals	118859.87	195882.45	*78581.27*	160974.85	*20756.61*	*70065.28*	*52612.97*	113553.38
Hospitals directly under the Health Commission	113799.45	190822.02	*73017.46*	155300.28	*12840.81*	*62149.47*	*44959.21*	105899.62

CNY, Chinese Yuan; Dominance, ICERs, incremental cost-effectiveness ratio; Lower cost, higher utility; LDL-C, low-density lipoprotein cholesterol; MI, myocardial infarction; NA, not applicable

italic text: ICERs below CNY 85698 (US$12741); bold text: ICERs above CNY 257094 (US$38224).

a: the maximum and minimum annual cost of statins, more detailed parameters are shown in Supplementary Tables S4

b: CV-related mortality costs used in other CEA studies and beyond the scope of sensitivity analysis, more detailed parameters are shown in Supplementary Tables S5

c: Utility used in other CEA studies and beyond the scope of sensitivity analysis, more detailed parameters are shown in Supplementary Tables S6

### Comparison of different modeling treatment effect methodologies

In the FOURIER trial population, the ICERs for the base case analysis and the 16 different scenario analyses generated by the treatment effect modeling approach based on clinical endpoints (from both perspectives, for two dosing regimens, respectively) were significantly higher than the corresponding ICERs generated by the treatment effect modeling approach based on reduced levels of LDL-C, with the former being essentially about 1.6 times higher than the latter. More detailed parameters are shown in Supplementary Tables S8, and S9.

## Discussion

As far as we know, the present study is the first CEA of evolocumab in treating patients with AMI as add-on therapy to statins from the perspectives of Chinese healthcare and private payers after evolocumab was admitted to the Chinese National Medical Insurance Reimbursement List and the drug price was decreased from 1298 CNY/140 mg to 283.8 CNY/140 mg. In this study, a Markov cohort state-transition model based on the FOURIER trial and CTTC meta-analysis was employed to investigate the cost-effectiveness of two dosing regimens (140 mg Q2W, 420 mg QM) of evolocumab in Chinese AMI populations from four separate clinical practice-based cohorts: the PEACE study population, the SuValue® database population with LDL-C ≥ 100 mg/dl or ≥ 70 mg/dl, and the BERSON trial population. The evaluation found that evolocumab had a lower ICER than the WTP at a current list price of 283.8 CNY/140 mg in all base-case CEA. Furthermore, the ICERs for the base-case CEA with evolocumab 140 mg Q2W were lower than the GDP per capita in the SuValue® database population with LDL-C ≥ 70 mg/dl or LDL-C ≥ 100 mg/dl.

The ICERs for sensitivity analysis and scenario analysis were mostly below the WTP, with a few exceptions, i.e., scenarios with shortened time horizons. After conversion, the ICERs in the scenario analyses with a time horizon cutoff before age 75 mostly exceeded the WTP. The probabilities that adding evolocumab to statins was cost-effective ranged from 76 to 98% in different populations. A series of scenario analyses revealed that ICERs became progressively smaller with increasing time horizons, and when the time horizon increased from 5 to 35 years, the corresponding ICERs decreased by around 80%. The older the starting age, the smaller the corresponding ICERs, and when the starting age increased from baseline to 75 years, the corresponding ICERs decreased by 24–56%. As the hospital hierarchy gradually upgraded, the hospitalization costs for various disease states also gradually increased (Supplementary Table S6), and the corresponding ICERs gradually decreased. When the hospital hierarchy upgraded from County hospitals to hospitals directly under the Health Commission, the hospitalization costs for various disease states increased by 148–289%, and the corresponding ICERs decreased by 11–62%. As the annual cost of statins increased, the corresponding ICERs gradually incremented. However, when the annual cost of statins rose from the lowest to the highest value (from CNY 118 to CNY 5470), the corresponding ICERs incremented by no more than 6%, indicating that the price changes of statins had a relatively weak effect on ICERs of evolocumab. As the annual cost of evolocumab increased, the corresponding ICERs also grew gradually. When the annual cost of evolocumab increased by 40% (from CNY 7404 to CNY 10366), the corresponding ICERs grew by 56–179%. In addition, the more significant the difference in the health utility values between the various disease states and the baseline state, i.e., the more severe the disease, the lower the corresponding ICERs.

Although the ICERs of all four populations in this study were lower than the WTP, the ICERs of the four populations differed considerably. The ICERs of the PEACE study population were much higher than the ICERs of the BERSON trial population and the SuValue® database population with LDL-C levels ≥ 100 mg/dl or ≥ 70 mg/dl. The reason might be: first, the treatment effect modeling approaches used in the economic evaluation for the different populations were different, with that for the PEACE study population being based on clinical outcomes and that for the rest of the population being based on LDL-C reduction levels. This study has validated that the ICERs from the clinical endpoint-based modeling approach were significantly higher than the corresponding ICERs from the modeling approach based on reduced LDL-C levels when the background conditions were equivalent; second, the age of both the PEACE study population and the BERSON trial population was lower than that of the SuValue® database population (61 versus 69).

To date, there have been three studies on the cost-effectiveness of evolocumab in China setting [14–16]. Two of the three studies compared evolocumab plus statins versus statins alone, and both studies were based on a Markov cohort state-transition model from Chinese healthcare perspective [14, 16]. The study of Zhe et al. was conducted before the drug price reduction and therefore has limited reference to the current [14]. The study of Xi et al. reported in 2023 on the cost-effectiveness of evolocumab in treating Chinese ASCVD patients with LDL-C ≥ 70 mg/dl [16]. Its results showed that compared to statins (627.80 CNY annually per person), the ICER for evolocumab was 14969 CNY per QALY gained based on the price of 283.8 CNY/140 mg and a dosing regimen of 140 mg Q2W. This result was much lower than the vast majority of results in the present study for the following reasons: first, the modeling approach for the effectiveness of treatment in this study was derived from the reduction of LDL-C levels, which would have produced lower results. Second, the starting age of the population in this study was 69 years, which was older than the starting age in the current study (around 60 years). Finally, most of the treatment costs in the study were derived from data for the Beijing region (one of the most developed regions in China), which was much higher than the treatment costs used in the present study (i.e., the officially reported national average).

Compared with previous studies in China, the present study had several advantages. Firstly, in terms of model structure, the model structure of this study was extracted from recently published domestic [15, 16] and foreign studies [19, 20]. The effects of LDL-C level, age, and history of cardiovascular events on subsequent cardiovascular events were fully considered [45]. The parameters used in the model (RR, HR) were selected from Chinese [22] or Asian [24] populations as far as possible. In addition, this study was the first to validate the differences in outcomes produced by different treatment effect modeling approaches (based on clinical endpoints or based on reductions in LDL-C levels), and to fully account for the results generated by different modeling approaches in the study. Secondly, in terms of the costs of disease treatment, the vast majority of the data employed in this study were derived from officially reported national [2] or health insurance data [25, 26] rather than from a single local data [14, 16]. Also, considering the enormous cost differences between different levels of hospitals, we conducted a series of scenario analyses. In addition, this study was the first to include indirect costs into cost considerations, such as lost wages and informal care costs, which had yet to be considered in previous studies. Thirdly, in terms of drug costs, for statins, this study did not consider only the average cost as in previous studies but fully considered the effect of centralized procurement on the drug price, and in addition to the average price, discussed the effect of the highest and lowest prices of centralized procurement on the study results separately; for evolocumab, this study examined the cost of all officially recommended dosing regimens (140 mg Q2W and 420 mg QM) separately, instead of considering only one dosing regimen (140 mg Q2W) as in previous studies. Fourthly, in terms of health utility values, the health utility values in this study were derived from a series of surveys of the Chinese population [21, 32–35], and taking into account the variability in survey respondents and survey regions, this study also conducted scenario analysis for some data beyond the scope of sensitivity analysis. Finally, in terms of study subjects, we evaluated the previous study populations separately and compared these results, considering that there were no clinical studies reported in China that were entirely consistent with the inclusion criteria of the FOURIER trial and that the study populations of previous studies were at various baseline levels.

In summary, the addition of evolocumab to statin therapy in patients with AMI was likely to be a cost-effective therapeutic option from the perspectives of Chinese healthcare and private payers at current pricing, but this did not mean that it could be used arbitrarily from the economic viewpoint. Therefore, when determining medicare reimbursement policies for evolocumab, the age, dosage, and length of adherence of users of this regimen, as well as the LDL-C value of the initiating therapy, should be clearly and strictly defined. From the perspective of private payers, starting age, dose, duration of use, and starting LDL-C values could be appropriately liberalized somewhat because of the indirect costs involved.

### Limitations

Despite the many advantages of the present study, there were still some limitations: First, due to the lack of relevant data on the incidence of CV events and the probabilities of recurrence in the Chinese population, this study had to extrapolate from the results of foreign studies such as the FOURIER trial and CTTC meta-analysis, which to a certain extent increases the uncertainty of the conclusions of this study. Second, the parameters in this study (e.g., costs, health utility values, and transfer probabilities) were derived from different studies that did not have entirely the same baseline levels, which may affect the accuracy of the results of the present study. Third, the data on the efficacy, safety, and adherence of evolocumab for long-term (lifetime) were from the FOURIER trial. However, the longest follow-up period in the FOURIER trial was only 168 weeks. While the OSLER-1 trial has demonstrated the efficacy, safety, and adherence of evolocumab over a 5-year follow-up period, additional studies are needed to validate parameters related to the longer-term use of evolocumab. Finally, the results of this study were only applicable to patients with AMI in the Chinese setting. They were not fully applicable to patients with other cardiovascular diseases, nor could they be directly applied to patients in other countries.

## Conclusions

From the Chinese healthcare and private payer perspectives, adding evolocumab (140 mg Q2W or 420 mg QM) to statin therapy in AMI patients is more likely to be a cost-effective treatment option at the current list price of CNY 283.8. The probabilities that adding evolocumab to statins was cost-effective ranged from 76 to 98% in different populations. However, evolocumab may not be cost-effective if used for shorter periods of time. The dosing regimen of 140 mg Q2W is more cost-effective than the dosing regimen of 420 mg QM. The earlier evolocumab is used, the longer it is used, the sicker the patient is, and the higher the hierarchy of hospitals, the more cost-effective evolocumab becomes. The results based on different treatment effect modeling approaches (clinical endpoints or LDL-C level reduction) were significantly different, so this factor should be fully considered when conducting further similar studies.

### Electronic supplementary material

Below is the link to the electronic supplementary material.

Supplementary Material 1

## Data Availability

The datasets generated during and/or analyzed during the current study are available from the first author on reasonable request.

## Code Availability

The code generated during and/or analyzed during the current study are available from the first author on reasonable request.
