# Supplementary material for: Methodology and results of cost-effectiveness of LDL-C lowering with evolocumab in patients with acute myocardial infarction in China
Source: Cost Eff Resour Alloc. 2023 Dec 1;21:93. doi: 10.1186/s12962-023-00501-4 (PMC10690971; doi:10.1186/s12962-023-00501-4)
Supplement: Supplementary file 1 — Supplementary Material 1 [file 12962_2023_501_MOESM1_ESM.docx]

**Methodology and results of Cost-effectiveness of LDL-C lowering with evolocumab in patients with Acute myocardial infarction in China**

Yuansheng Wan^1^, Jinyu Liu ^2^, Xiaolian Zhan^1^, Yu Zhang^1^, Ruxu You^#1^

1 Department of Pharmacy, Union Hospital, Tongji Medical College, Huazhong University of Science and Technology, Wuhan, Hubei, China

2 Department of Pharmacy, Tongji Hospital, Tongji Medical College, Huazhong University of Science and Technology, Wuhan, Hubei, China

**^#^Correspondence:** Ruxu You

Address: Department of Pharmacy, Union Hospital, Tongji Medical College, Huazhong University of Science and Technology, 1277 Jiefang Avenue, Wuhan 430022, China.

E-mail: youruxu2008@163.com

**Online supplement**

Table S1 Consolidated Health Economic Evaluation Reporting Standards 2022 (CHEERS 2022)

Table S2 Hazard ratios for major adverse CV events history

Table S3 Baseline characteristics of the model population

Table S4 Details of dosage regimens for all interventions considered in this study

Table S5 CV-related mortality costs used in other CEA studies and beyond the scope of sensitivity analysis

Table S6 Utility used in other CEA studies and beyond the scope of sensitivity analysis

Table S7 Average hospitalization costs of diseases at all levels of hospitals

Table S8 ICERs for 16 different scenario analyses of 2 different modelling effectiveness ways of evolocumab 140mg Q2W in the FOURIER trial population

Table S9 ICERs for 16 different scenario analyses of 2 different modelling effectiveness ways of evolocumab 420mg QM in the FOURIER trial population

Figure S1 Tornado plots for one-way deterministic sensitivity analysis of the BERSON study population

Figure S2 Tornado plots for one-way deterministic sensitivity analysis of the SuValue® database population with LDL-C levels ≥100 mg/dL

Figure S3 Tornado plots for one-way deterministic sensitivity analysis of the SuValue® database population with LDL-C levels ≥70 mg/dL

Figure S4 Monte Carlo simulation scatters plot in probabilistic sensitivity analyses of the BERSON study population

Figure S5 Monte Carlo simulation scatters plot in probabilistic sensitivity analyses of the SuValue® database population with LDL-C levels ≥100 mg/dL

Figure S6 Monte Carlo simulation scatters plot in probabilistic sensitivity analyses of the SuValue® database population with LDL-C levels ≥70 mg/dL

Figure S7 Cost-effectiveness acceptability curves in probabilistic sensitivity analyses of the BERSON study population

Figure S8 Cost-effectiveness acceptability curves in probabilistic sensitivity analyses of the SuValue® database population with LDL-C levels ≥100 mg/dL

Figure S9 Cost-effectiveness acceptability curves in probabilistic sensitivity analyses of the SuValue® database population with LDL-C levels ≥70 mg/dL

Table S1: Consolidated Health Economic Evaluation Reporting Standards 2022 (CHEERS 2022)[1]

| **Topic** | **No.** | **Item** | **Location where item is reported** |
| --- | --- | --- | --- |
| **Title** |  |  |  |
|  | 1 | Identify the study as an economic evaluation and specify the interventions being compared. | Title page |
| **Abstract** |  |  |  |
|  | 2 | Provide a structured summary that highlights context, key methods, results, and alternative analyses. | Abstract |
| **Introduction** |  |  |  |
| Background and objectives | 3 | Give the context for the study, the study question, and its practical relevance for decision making in policy or practice. | Introduction |
| **Methods** |  |  |  |
| Health economic analysis plan | 4 | Indicate whether a health economic analysis plan was developed and where available. | Section "Methods", first paragraph |
| Study population | 5 | Describe characteristics of the study population (such as age range, demographics, socioeconomic, or clinical characteristics). | Subsection "Patient population"; Supplementary material and Table S1 |
| Setting and location | 6 | Provide relevant contextual information that may influence findings. | Section "Methods", first paragraph |
| Comparators | 7 | Describe the interventions or strategies being compared and why chosen. | Section "Base case cost-effectiveness analysis" |
| Perspective | 8 | State the perspective(s) adopted by the study and why chosen. | Section "Methods", first paragraph |
| Time horizon | 9 | State the time horizon for the study and why appropriate. | Section "Methods",first paragraph |
| Discount rate | 10 | Report the discount rate(s) and reason chosen. | Section "Base case cost-effectiveness analysis" |
| Selection of outcomes | 11 | Describe what outcomes were used as the measure(s) of benefit(s) and harm(s). | Section "Base case cost-effectiveness analysis" |
| Measurement of outcomes | 12 | Describe how outcomes used to capture benefit(s) and harm(s) were measured. | Section "Base case cost-effectiveness analysis" and "Utility" |
| Valuation of outcomes | 13 | Describe the population and methods used to measure and value outcomes. | Not Applicable |
| Measurement and valuation of resources and costs | 14 | Describe how costs were valued. | Subsection "costs" and "Utility" |
| Currency, price date, and conversion | 15 | Report the dates of the estimated resource quantities and unit costs, plus the currency and year of conversion. | Subsection "costs" |
| Rationale and description of model | 16 | If modelling is used, describe in detail and why used. Report if the model is publicly available and where it can be accessed. | Subsection "Model structure" |
| Analytics and assumptions | 17 | Describe any methods for analysing or statistically transforming data, any extrapolation methods, and approaches for validating any model used. | Subsections "Model structure", "Treatment effects" and "Mortality" |
| Characterising heterogeneity | 18 | Describe any methods used for estimating how the results of the study vary for subgroups. | Subsections "Patient population", Supplementary material and Table S1 |
| Characterising distributional effects | 19 | Describe how impacts are distributed across different individuals or adjustments made to reflect priority populations. | Not applicable |
| Characterising uncertainty | 20 | Describe methods to characterise any sources of uncertainty in the analysis. | Subsection "Sensitivity analyses" and "Scenario Analyses" |
| Approach to engagement with patients and others affected by the study | 21 | Describe any approaches to engage patients or service recipients, the general public, communities, or stakeholders (such as clinicians or payers) in the design of the study. | Subsection "Model validation" |
| **Results** |  |  |  |
| Study parameters | 22 | Report all analytic inputs (such as values, ranges, references) including uncertainty or distributional assumptions. | Tables 1, Supplementary material: Table S3-S6 |
| Summary of main results | 23 | Report the mean values for the main categories of costs and outcomes of interest and summarise them in the most appropriate overall measure. | Subsection "Base-case analysis" |
| Effect of uncertainty | 24 | Describe how uncertainty about analytic judgments, inputs, or projections affect findings. Report the effect of choice of discount rate and time horizon, if applicable. | Subsection "Sensitivity analyses" and "Scenario Analyses" |
| Effect of engagement with patients and others affected by the study | 25 | Report on any difference patient/service recipient, general public, community, or stakeholder involvement made to the approach or findings of the study | Not applicable |
| **Discussion** |  |  |  |
| Study findings, limitations, generalisability, and current knowledge | 26 | Report key findings, limitations, ethical or equity considerations not captured, and how these could affect patients, policy, or practice. | Section Discussion |
| **Other relevant information** |  |  |  |
| Source of funding | 27 | Describe how the study was funded and any role of the funder in the identification, design, conduct, and reporting of the analysis | Subsection "Funding" |
| Conflicts of interest | 28 | Report authors conflicts of interest according to journal or International Committee of Medical Journal Editors requirements. | Subsection "Conflicts of Interest" |

Table S2 Hazard ratios for major adverse CV events history[2]

| Risk factor | Hazard ratio | |  |
| --- | --- | --- | --- |
| Age (years) | 1.06 | 1.06 - 1.06 | Lognormal |
| MI | 1.13 | 1.04 - 1.22 | Lognormal |
| IS | 1.13 | 0.99 - 1.30 | Lognormal |
| 2+ MI | 1.19 | 1.05 - 1.34 | Lognormal |
| 2+ IS | 1.36 | 1.03 - 1.80 | Lognormal |
| MI and IS | 1.94 | 1.23 - 3.04 | Lognormal |
| oASCVD and MI | 1.31 | 1.24 - 1.38 | Lognormal |
| oASCVD and IS | 1.40 | 1.23 - 1.60 | Lognormal |
| oASCVD and MI and IS | 1.93 | 1.47 - 2.54 | Lognormal |
| CV event in past year (yes/no) | 1.50 | 1.39 - 1.62 | Lognormal |
|  | 3.85 | 3.36 - 4.37 | Lognormal |
| Diabetes | 1.55 | 1.49 - 1.62 | Lognormal |

CV, cardiovascular; IS, ischaemic stroke; MI, myocardial infarction; oASCVD, other ASCVD

**Population**

Real-world data were employed in this study to model the AMI population in clinical practice that reflected the representative characteristics of the Chinese MI patient population. Patients were extracted separately from three recently published, clinical practice-based studies, i.e., China Patient-Centered Evaluative Assessment of Cardiac Events Prospective Study of AMI (China PEACE-Prospective AMI Study)[3], the standardized Chinese hospital-based health information system electronic database (SuValue®)[4, 5], and the Chinese population from the BERSON clinical trial (evolocumaB Efficacy study for LDL-C Reduction in subjectS with T_2_DM On background statiN)[6]. Detailed information is shown in Supplementary Table S2.

The China PEACE-Prospective AMI Study (2012–2014) [3] was the first and only longitudinal study on patient-reported outcomes and clinical events in AMI in China. The study enrolled 3415 AMI patients aged 18 years or older (mean age, 60.7 years) from 53 hospitals located in 21 provinces in China. Throughout one year after AMI, 76.9% of enrolled patients were adherent to statin therapy. One-year clinical outcomes included cardiac mortality (2.2%), non-fatal AMI (1.7%), and stroke (0.9%).

The SuValue® database was a standardized electronic database of Chinese health information systems for hospital settings, including information on more than 90 million patients from 168 hospitals in China with 10-year longitudinal data. A total of 17966 patients with ASCVD who received high-intensity statin therapy and had LDL-C levels ≥100mg/dL (≈ 2.6 mmol/L, target values for lipid-regulating therapy in high-risk patients[7, 8]) were ascertained from the database. The average age was 69 years, the mean LDL-C was 132.7 mg/dL, and the CV event rate per 100 patient-years at baseline was 9.09[4]. Similarly, 31411 ASCVD patients with LDL-C levels ≥ 70mg/dl (≈ 1.8 mmol/L, target values for lipid-regulating therapy in extremely high-risk patients[7, 8]) and treated with high-intensity statins were collected from the database. The average age was 69 years, the mean LDL-C was 112.92mg/dL, and the CV event rate per 100 patient-years at baseline was 6.65[5]. Baseline CV event rates were generated from the clinical results of the FOURIER study[9] and adapted by age and LDL-C levels with a standard published methodology[2].

The BERSON study was a double-blind, randomized, controlled study performed at 98 centers in 10 countries[10]. Approximately 50% of the subjects in this study were recruited from China. The study evaluated the efficacy and safety of evolocumab in combination with atorvastatin in patients with T_2_DM and hyperlipidemia or mixed dyslipidemia. For the 451 Chinese patients in the BERSON study[6], LDL-C ≥100mg/dL (≈2.6 mmol/L) was required for patients receiving statins at screening. The average age was 60 years, and the mean LDL-C was 89mg/dL (2.3 mmol/L). Baseline CV event rates were generated from the above methods.

Table S3 Baseline characteristics of the model population

| **Characteristics** | FOURIER population (n=27,564) | PEACE population (n=3415) | BERSON population (Chinese, n=451) | SuValue®, LDL-C >= 100 mg/dL | SuValue®, LDL-C >= 70 mg/dL |
| --- | --- | --- | --- | --- | --- |
| **Demographics** |  |  |  |  |  |
| Age, mean (SD), y /median age (range), y | 62.5 (9.0) | 60.7 (11.9) | 61 (33-79) | 69 (12.98) | 69 (12.98) |
| Female sex, No. (%) | 6769 (24.6) | 791 (23.2) | 230 (51.0) | 53% | 50% |
| LDL-C, mean (SD), mg/dL | 97.2 (27.9) | NA | 88.9 (34.8) | 132.7 (29.82) | 112.92 |
| **Cardiovascular risk factors, No. (%)** |  |  |  |  |  |
| Hypertension | 22,084 (80.1) | 1899 (55.6) | 279 (61.9) | NA | NA |
| Diabetes mellitus | 10,081 (36.6) | 794 (23.3) | **451 (100)** | NA | NA |
| Current cigarette use | 7777 (28.2) | 1994 (58.4) | NA | NA | NA |
| **Cardiovascular medications, No. (%)** |  |  |  |  |  |
| Statins | 27,495 (99.7) | 2561 (75.0) | 175 (38.8) | NA | NA |
| Aspirin or/and P2Y12 inhibitor | 25,432 (92.3) | 2616 (76.6) | NA | NA | NA |
| Beta-blockers | 20,815 (75.5) | 1827 (53.5) | NA | NA | NA |
| ACEIs or ARBs | 21,533 (78.1) | 1448 (42.4) | NA | NA | NA |
| **LDL-C reduction with Evo, mmol/L (%)** | 1.45 (59%) | NA | 140mg Q2W:1.88 (88%); 420mg QM: 1.69(74.9%) | 66.00%[11] | 66.00%[11] |
| **MACEs per year, No. (%)** |  |  |  |  |  |
| Death from vascular cause | 223 (0.8) | 75 (2.2) | NA | NA | NA |
| Death from any cause | 395 (1.4) | 105 (3.1) | NA | NA | NA |
| MI | 503 (1.8) | 58 (1.7) | NA | NA | NA |
| Stroke | 213 (0.8) | 30 (0.9) | NA | NA | NA |
| Coronary revascularization | 784 (2.8) | 219 (6.4) | NA | NA | NA |

ACEI: angiotensin-converting enzyme inhibitor; ARB: angiotensin-receptor blocker; MACE: major adverse cardiovascular event; MI: Myocardial infarction; NA, not applicable; SD: standard deviation.

Table S4 Details of dosage regimens for all interventions considered in this study[12]

| Intensity | Drug name | Dosage | Cost per unit | Range of cost per unit (CNY) | | | Annual median cost (CNY) | | |
| --- | --- | --- | --- | --- | --- | --- | --- | --- | --- |
|  |  |  | (median, CNY) | min | max | median | min | max | median |
| Low | Atorvastatin | 10mg | 2.00 | 0.09 | 6.59 | 2.00 | 118.86 | 5470.28 | 2855.15 |
|  | Fluvastatin | 20mg | 1.66 |  |  |  |  |  |  |
|  |  | 40mg | 2.82 |  |  |  |  |  |  |
|  | Pravastatin | 10mg | 1.85 |  |  |  |  |  |  |
|  |  | 20mg | 3.70 |  |  |  |  |  |  |
|  | Pitavastatin | 1mg | 3.00 |  |  |  |  |  |  |
|  | Rosuvastatin | 5mg | 2.23 |  |  |  |  |  |  |
|  | Simvastatin | 5mg | 0.52 |  |  |  |  |  |  |
|  |  | 10mg | 0.69 |  |  |  |  |  |  |
| Moderate | Atorvastatin | 20mg | 3.95 | 0.11 | 12.72 | 4.21 |  |  |  |
|  | Fluvastatin | 80mg | 4.47 |  |  |  |  |  |  |
|  | Pravastatin | 40mg | 8.22 |  |  |  |  |  |  |
|  | Pitavastatin | 2mg | 5.03 |  |  |  |  |  |  |
|  |  | 4mg | 11.70 |  |  |  |  |  |  |
|  | Simvastatin | 20mg | 1.25 |  |  |  |  |  |  |
|  |  | 40mg | 3.80 |  |  |  |  |  |  |
|  | Rosuvastatin | 10mg | 3.37 |  |  |  |  |  |  |
| High | Atorvastatin | 40mg | 10.39 | 0.42 | 16.00 | 9.42 |  |  |  |
|  | Rosuvastatin | 20mg | 9.42 |  |  |  |  |  |  |
|  | Simvastatin | 80mg | 6.55 |  |  |  |  |  |  |
|  | Atorvastatin | 20mg |  | 0.12 | 6.11 | 3.95 | 44.87 | 2231.68 | 1442.74 |

CNY, Chinese Yuan

The weights of low, medium, and high intensity were based on the baseline of the FOURIER test.

Table S5 CV-related mortality costs used in other CEA studies and beyond the scope of sensitivity analysis (CNY)

|  | Cost of cardiovascular events | | Costs of CV-related death | | Source |
| --- | --- | --- | --- | --- | --- |
|  | direct cost | Indirect cost | direct cost | Indirect cost |  |
| Death due to stroke 1 | 60714.12 | 3647.27 | 31526.3 | 3647.27 | [13] |
| Death due to stroke 2 | 29843.85 | 3395.74 | 20670.35 | 3395.74 | [14] |
| Death due to MI 1 | 54141.41 | 2945.49 | 46603.83 | 2945.49 | [15] |
| Death due to MI 2 | 72597.75 | 1705.41 | 89692.91 | 1705.41 | [16] |

CEA, cost-effectiveness analysis; CNY, Chinese Yuan; CV, cardiovascular; MI, myocardial infarction;

Table S6 Utility used in other CEA studies and beyond the scope of sensitivity analysis

|  | Health status | Value | Source |
| --- | --- | --- | --- |
| Higher utility | Nonfatal stroke (year 1) | 0.746 | [17] |
|  | Nonfatal stroke (beyond 1y) | 0.880 | [18] |
| Lower utility | Nonfatal stroke (beyond 1y) | 0.618 | [19] |
| UK- utility | MI | 0.672 | [20] |
|  | Post-MI | 0.824 |  |
|  | Stroke | 0.327 |  |
|  | Post- Stroke | 0.524 |  |

CEA, cost-effectiveness analysis; MI, myocardial infarction;

Table S7 Average hospitalization costs of diseases at all levels of hospitals (CNY)[21]

| **Hospital Grade** | Health status | Direct cost | Indirect cost |
| --- | --- | --- | --- |
| Hospitals directly under the Health Commission | Nonfatal myocardial infarction (year 1) | 38144.60 | 1886.52 |
|  | Nonfatal stroke (year 1) | 24258.72 | 2753.19 |
|  | Coronary revascularization | 123836.30 | 4351.57 |
| Provincial hospitals | Nonfatal myocardial infarction (year 1) | 34299.60 | 1987.13 |
|  | Nonfatal stroke (year 1) | 19112.08 | 2689.82 |
|  | Coronary revascularization | 116124.00 | 4854.64 |
| Prefecture-level municipal hospitals | Nonfatal myocardial infarction (year 1) | 28170.40 | 2188.36 |
|  | Nonfatal stroke (year 1) | 16069.37 | 2814.95 |
|  | Coronary revascularization | 117053.90 | 6087.17 |
| County-level municipal hospitals | Nonfatal myocardial infarction (year 1) | 21158.70 | 1987.13 |
|  | Nonfatal stroke (year 1) | 10516.31 | 2585.46 |
|  | Coronary revascularization | 89852.20 | 5986.56 |
| County hospitals | Nonfatal myocardial infarction (year 1) | 17044.90 | 1836.21 |
|  | Nonfatal stroke (year 1) | 8389.63 | 2481.74 |
|  | Coronary revascularization | 83165.00 | 5081.03 |

**Calculation:**

Indirect cost = Average length of stay * Average Daily Wage[22]

Table S8 ICERs for 16 different scenario analyses of 2 different modelling effectiveness ways of evolocumab 140mg Q2W in the FOURIER trial population (CNY/QALY)

|  | From the healthcare perspective | |  | From the private payer perspective | |
| --- | --- | --- | --- | --- | --- |
|  | Based on clinical endpoints | Based on LDL-C reduction |  | Based on clinical endpoints | Based on LDL-C reduction |
| **Base-case analyses** | 243017.60 | 146536.13 |  | 227586.39 | 135828.64 |
| **Time horizon** |  |  |  |  |  |
| 5 years | **1091329.37** | **794423.41** |  | **1067896.92** | **775131.75** |
| 10 years | **557623.37** | **372699.19** |  | **537150.77** | **356993.58** |
| 15 years | **368484.04** | 235744.02 |  | **350172.58** | 222236.30 |
| 20 years | **281929.71** | 174573.38 |  | **265335.38** | 162737.54 |
| 30 years | 228699.49 | 135296.35 |  | 213790.14 | 125103.88 |
| 35 years | 223875.44 | 130904.48 |  | 209047.56 | 120793.31 |
| **Starting age** |  |  |  |  |  |
| 65-year | 221173.82 | 129805.32 |  | 207499.43 | 120409.42 |
| 70-year | 178948.95 | 99120.04 |  | 168409.81 | 91813.62 |
| 75-year | 143128.40 | *75123.78* |  | 134615.03 | *68937.20* |
| **Discount rate** |  |  |  |  |  |
| 3% | 216548.99 | 129362.28 |  | 201617.95 | 119106.65 |
| 6% | **257140.63** | 155785.18 |  | 241457.42 | 144847.36 |
| **Hospital classification** |  |  |  |  |  |
| County hospitals | **264397.20** | 163200.98 |  | 249204.04 | 152659.65 |
| County-level municipal hospitals | **258285.18** | 158755.84 |  | 242626.62 | 147830.73 |
| Prefecture-level municipal hospitals | 241038.08 | 145255.53 |  | 225193.38 | 134207.07 |
| Provincial hospitals | 236405.67 | 142574.86 |  | 221189.22 | 132045.63 |
| Hospitals directly under the Health Commission | 229482.82 | 137522.90 |  | 214509.81 | 127197.72 |

CNY, Chinese Yuan; Dominance, ICERs, incremental cost-effectiveness ratio; Lower cost, higher utility; LDL-C, low-density lipoprotein cholesterol; QALY, quality-adjusted life-year.

italic text: ICERs below CNY 85698 (US$12741); bold text: ICERs above CNY 257094 (US$38224).

Table S9 ICERs for 16 different scenario analyses of 2 different modelling effectiveness ways of evolocumab 420mg QM in the FOURIER trial population (CNY/QALY)

|  | From the healthcare perspective | |  | From the private payer perspective | |
| --- | --- | --- | --- | --- | --- |
|  | Based on clinical endpoints | Based on LDL-C reduction |  | Based on clinical endpoints | Based on LDL-C reduction |
| **Base-case analyses** | **364770.76** | 226287.79 |  | **349339.55** | 215580.30 |
| **Time horizon** |  |  |  |  |  |
| 5 years | **1617798.82** | **1203911.02** |  | **1594366.37** | **1184619.35** |
| 10 years | **830643.67** | **569207.83** |  | **810171.07** | **553502.22** |
| 15 years | **550418.57** | **361423.99** |  | **532107.10** | **347916.27** |
| 20 years | **422146.96** | **268537.25** |  | **405552.62** | 256701.41 |
| 30 years | **344053.59** | 209820.92 |  | **329144.24** | 199628.45 |
| 35 years | **337472.22** | 203854.89 |  | **322644.35** | 193743.72 |
| **Starting age** |  |  |  |  |  |
| 65-year | **332152.09** | 200731.96 |  | **318477.71** | 191336.06 |
| 70-year | **269493.36** | 154325.81 |  | **258954.22** | 147019.39 |
| 75-year | **216851.15** | 118575.42 |  | 208337.78 | 112388.85 |
| **Discount rate** |  |  |  |  |  |
| 3% | **325477.46** | 200170.93 |  | **310546.42** | 189915.31 |
| 6% | **385743.14** | 240357.80 |  | **370059.92** | 229419.97 |
| **Hospital classification** |  |  |  |  |  |
| County hospitals | **386150.36** | 242952.64 |  | **370957.19** | 232411.31 |
| County-level municipal hospitals | **380038.33** | 238507.50 |  | **364379.77** | 227582.39 |
| Prefecture-level municipal hospitals | **362791.23** | 225007.19 |  | **346946.53** | 213958.73 |
| Provincial hospitals | **358158.83** | 222326.52 |  | **342942.37** | 211797.29 |
| Hospitals directly under the Health Commission | **351235.97** | 217274.56 |  | **336262.96** | 206949.37 |

CNY, Chinese Yuan; Dominance, ICERs, incremental cost-effectiveness ratio; Lower cost, higher utility; LDL-C, low-density lipoprotein cholesterol; QALY, quality-adjusted life-year.

bold text: ICERs above CNY 257094 (US$38224).


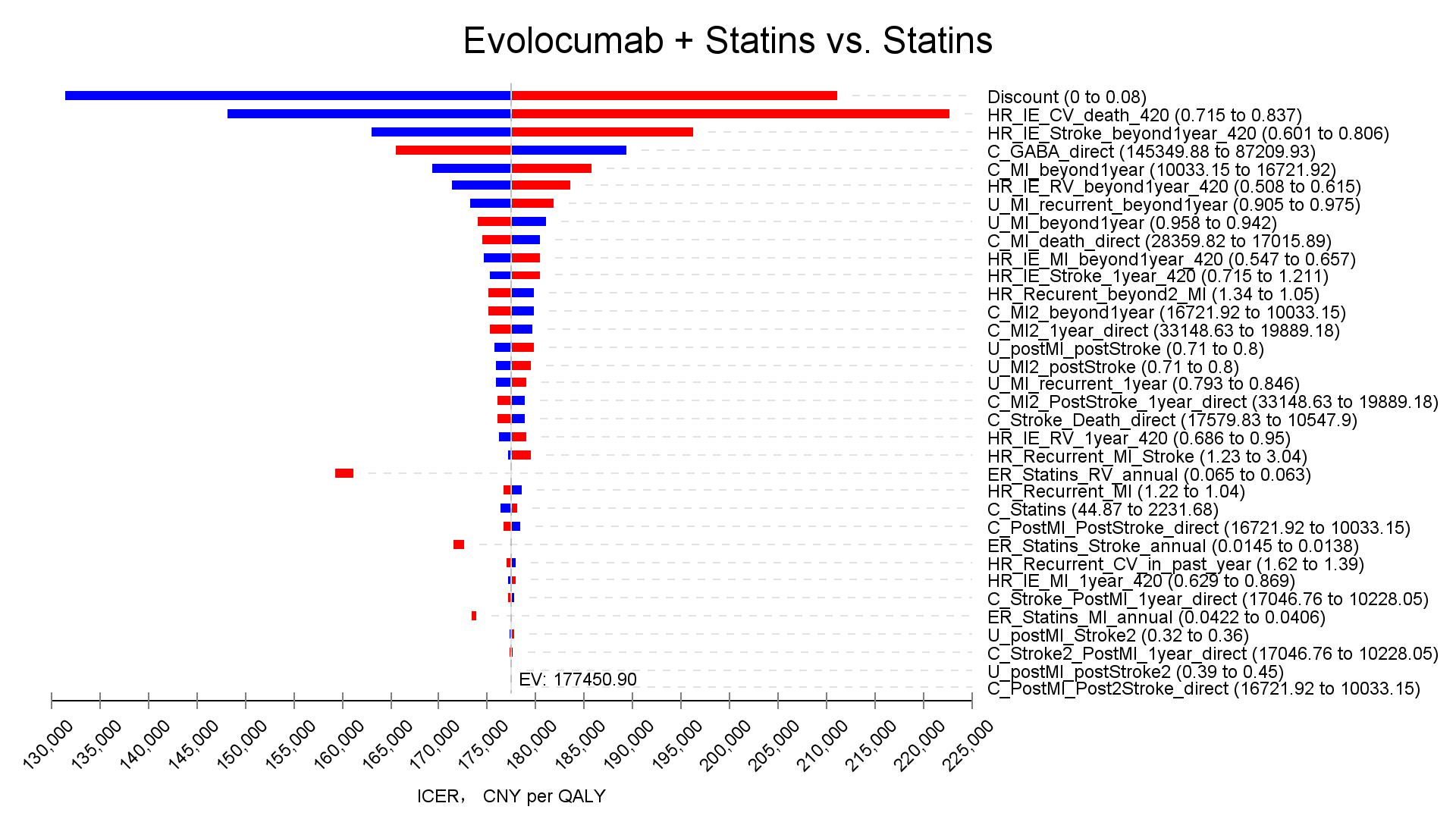

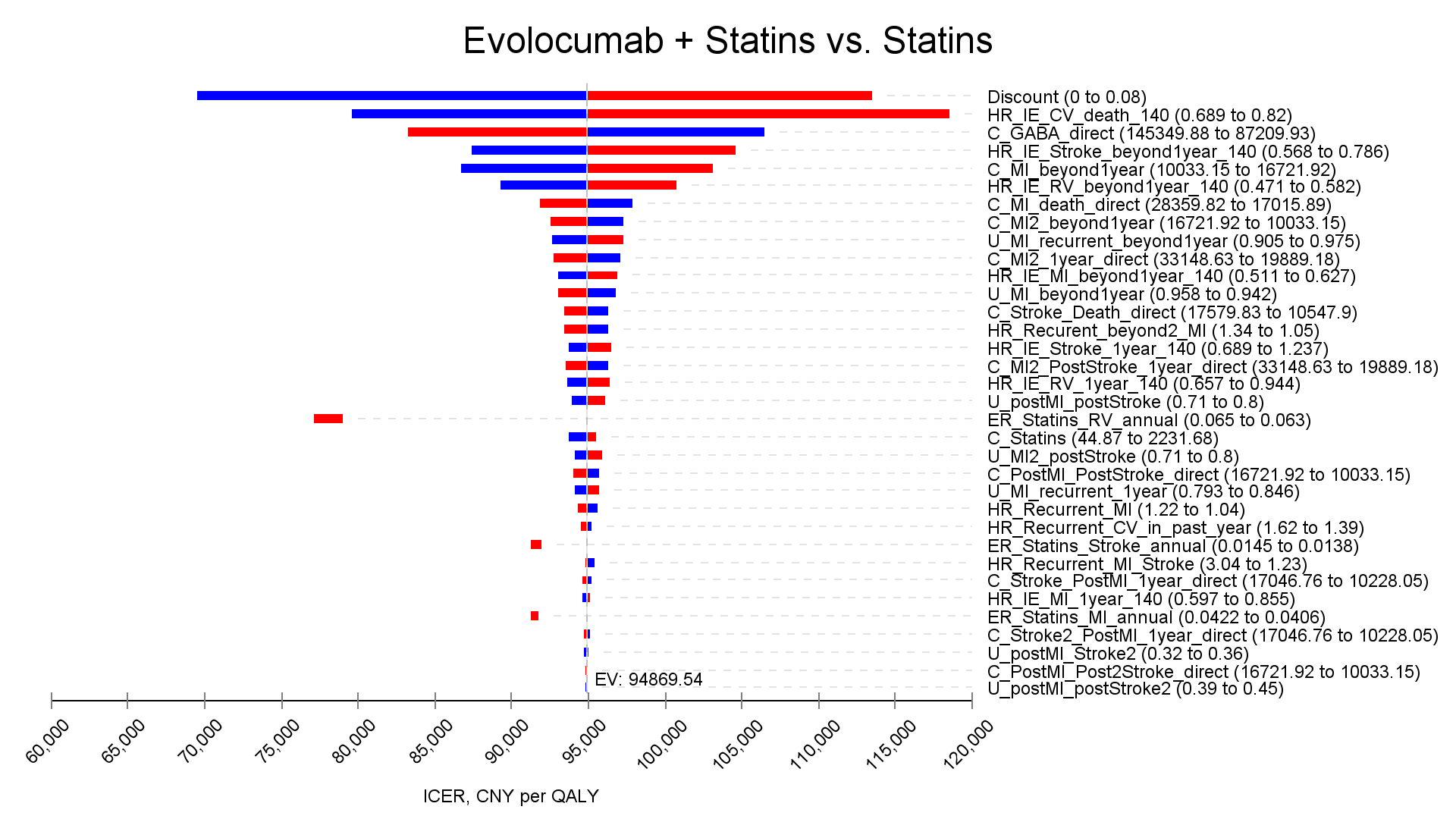


A evolocumab 140 mg Q2W B evolocumab 420 mg QM


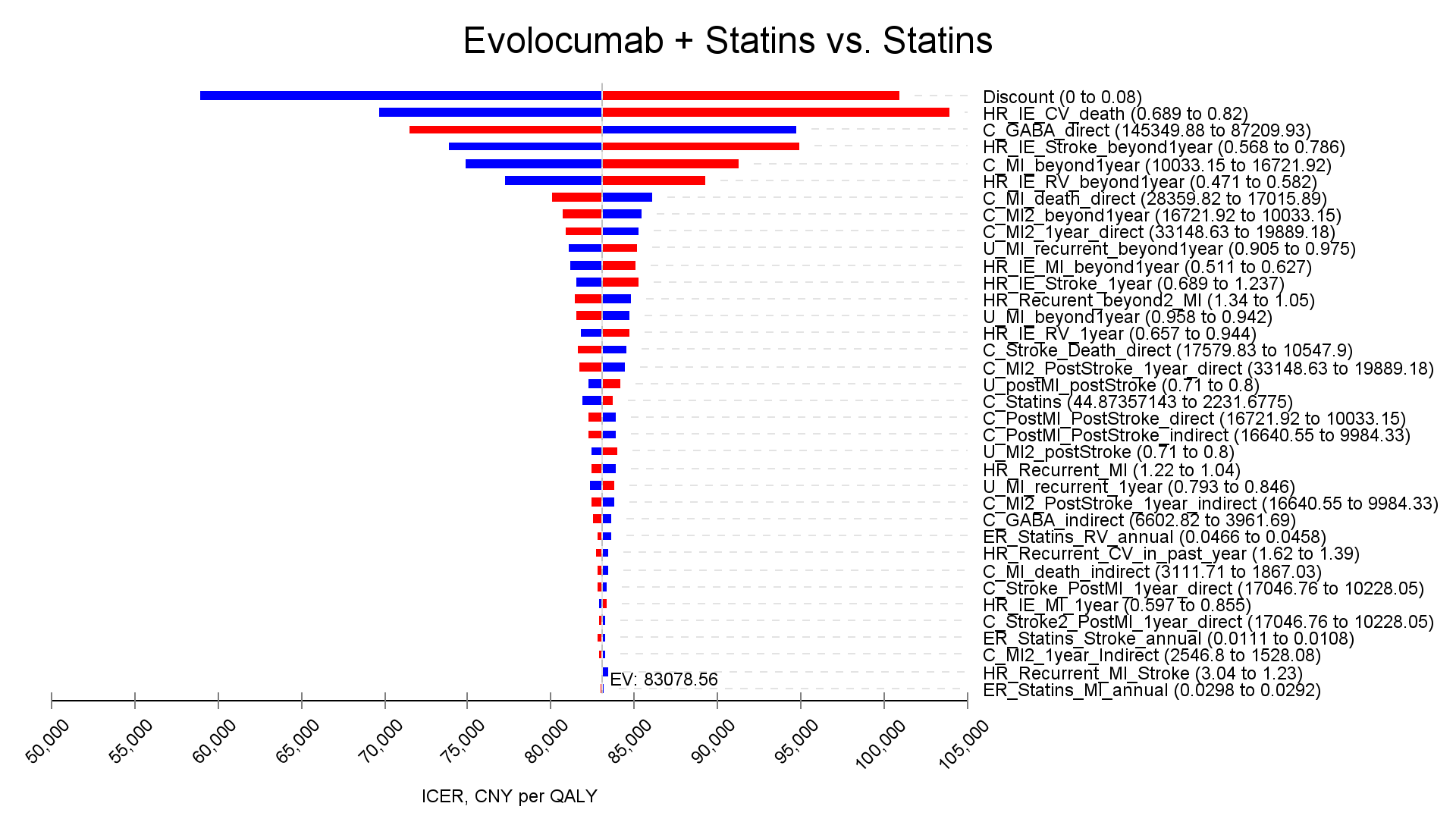

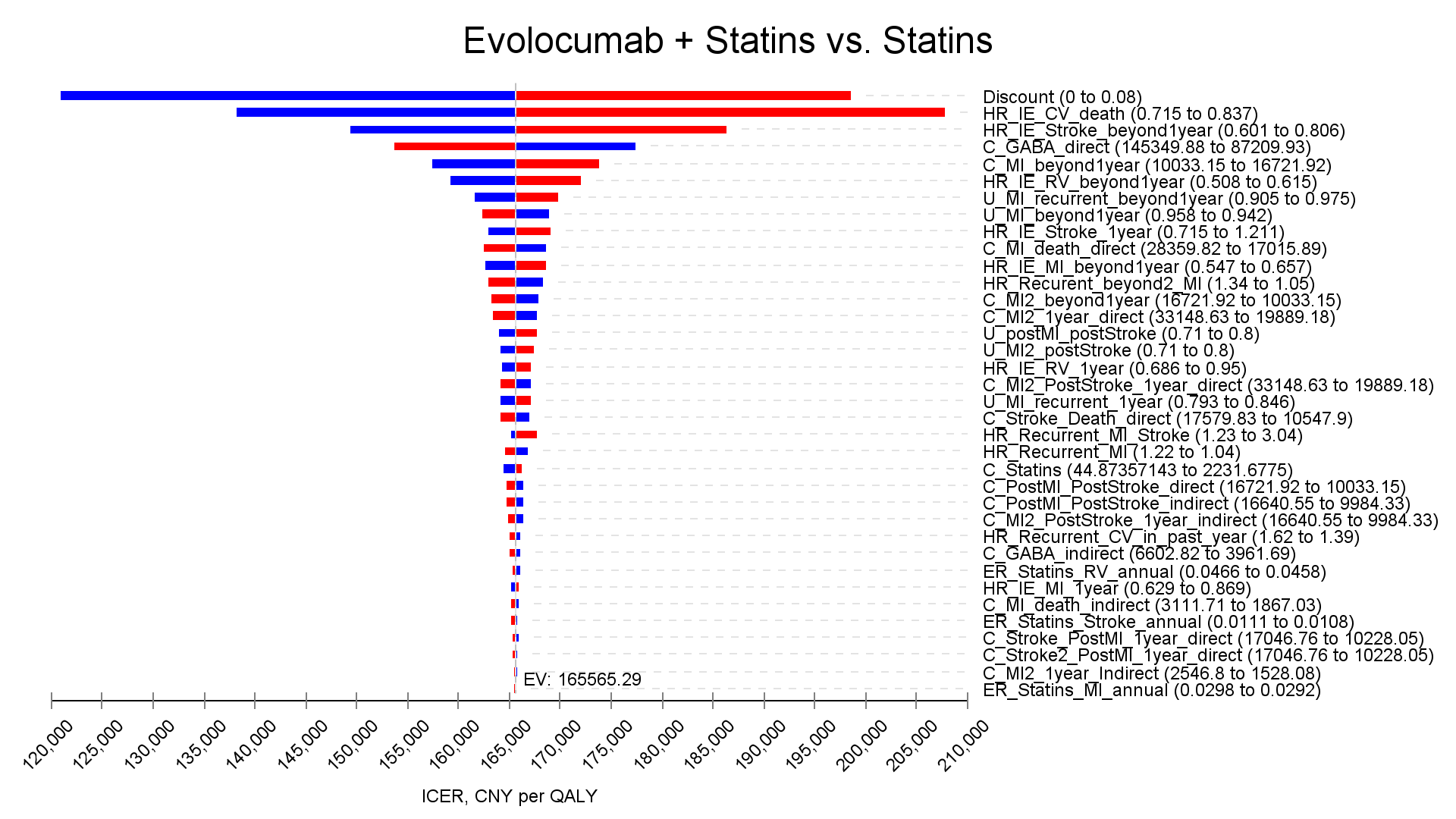


C evolocumab 140 mg Q2W D evolocumab 420 mg QM

Figure S1 Tornado plots for one-way deterministic sensitivity analysis of the BERSON study population

A and B are the results from the Chinese healthcare perspective, C and D are the results from the Chinese private payer perspective;

C_, Cost; CNY, Chinese yuan, CV: cardiovascular; ER_, event rate; EV, expected value; HR_, Hazard ratio; ICER, incremental cost-effectiveness ratio; IE_, Intervention effect; MI, myocardial infarction; RV_, revascularization; U_, Utility; WTP, willingness-to-pay.


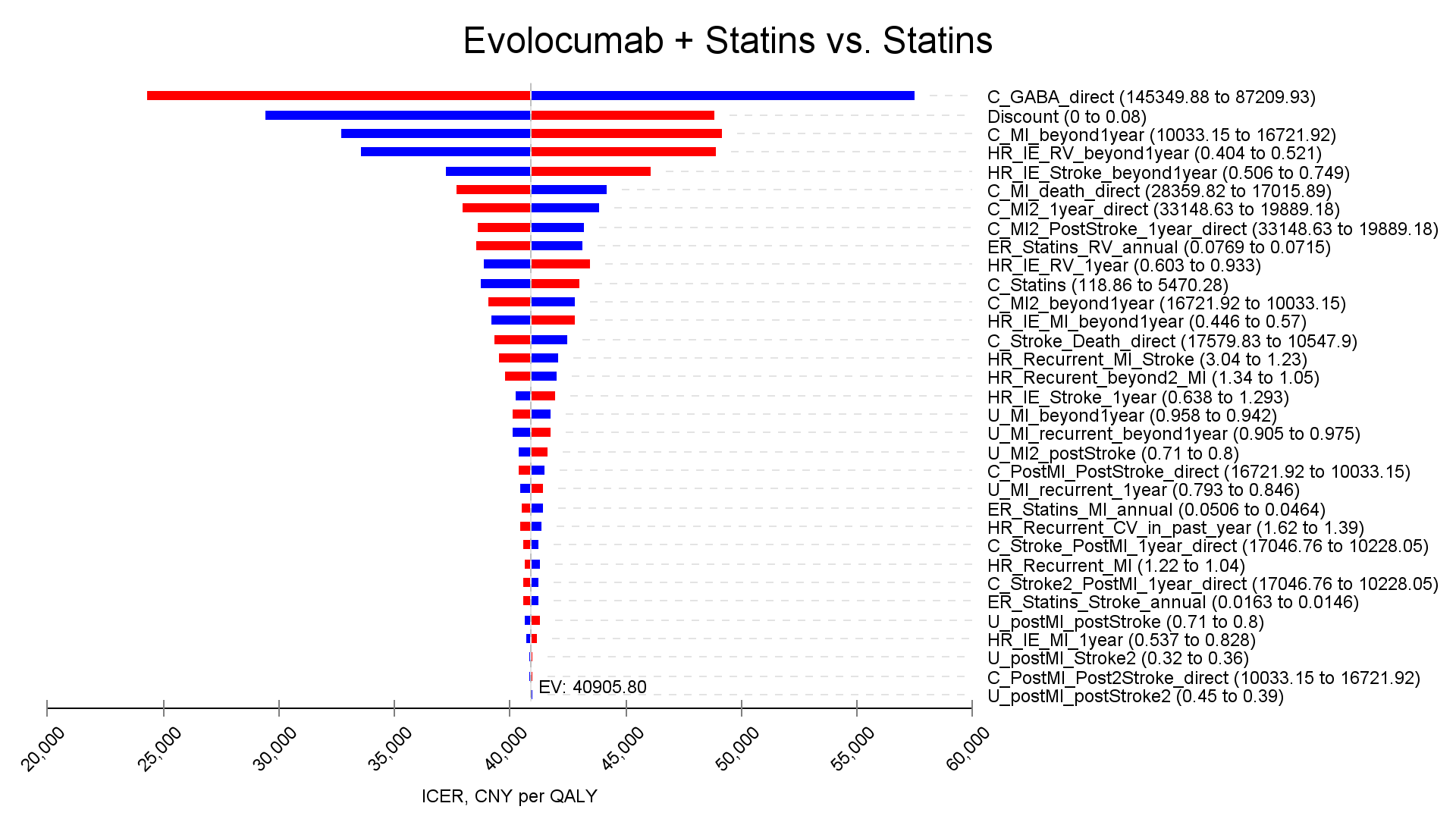

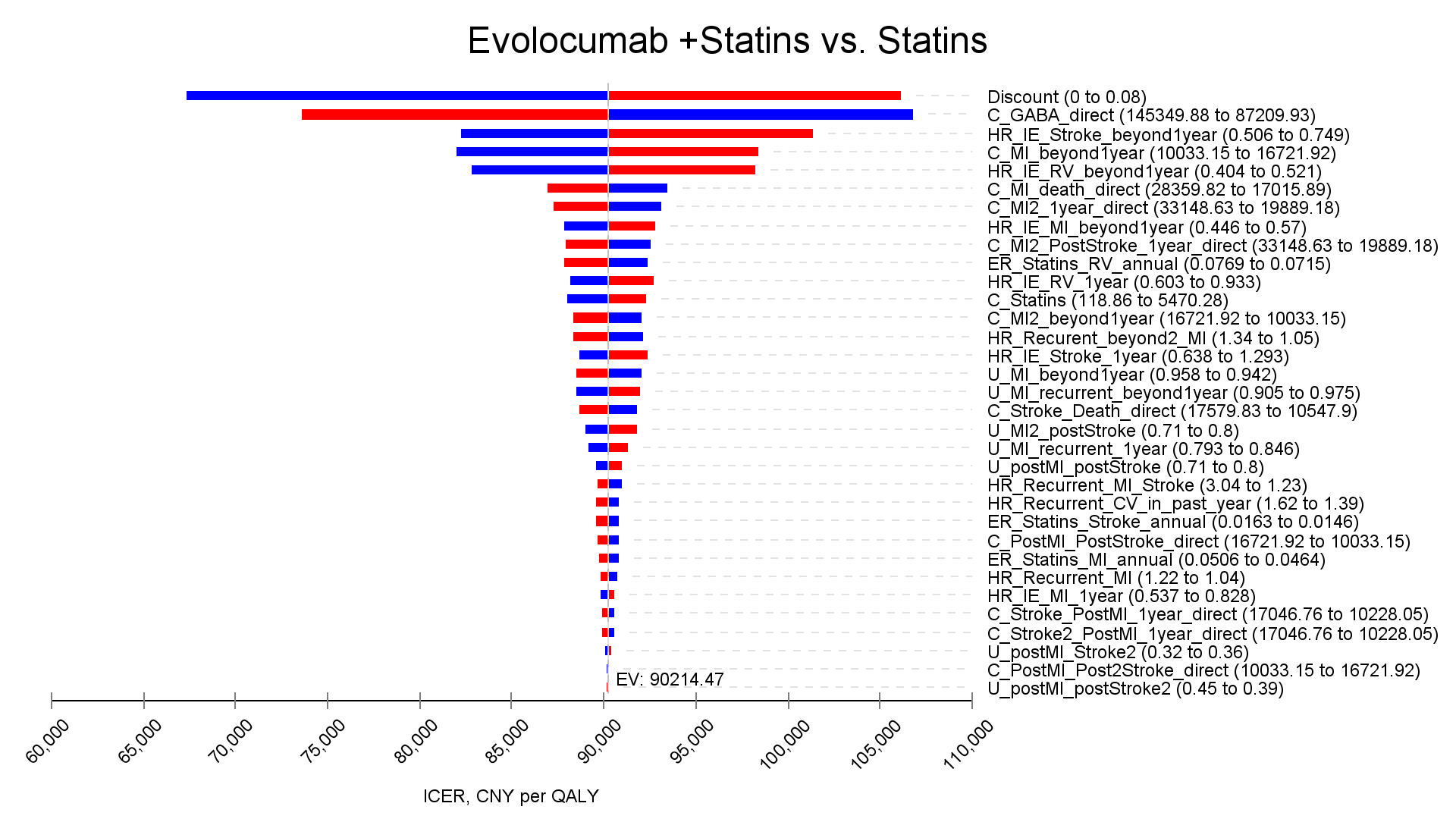


A evolocumab 140 mg Q2W B evolocumab 420 mg QM


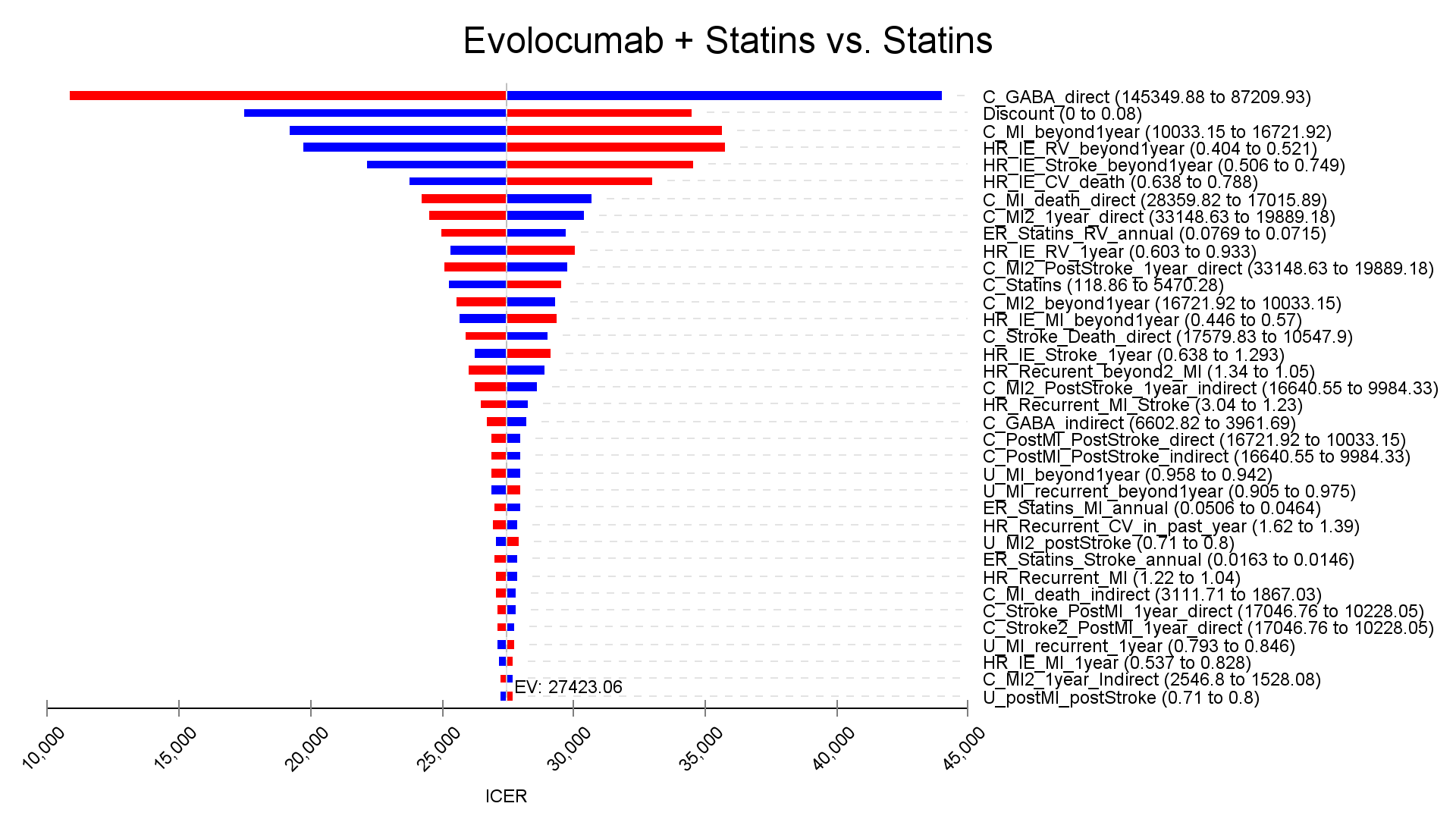

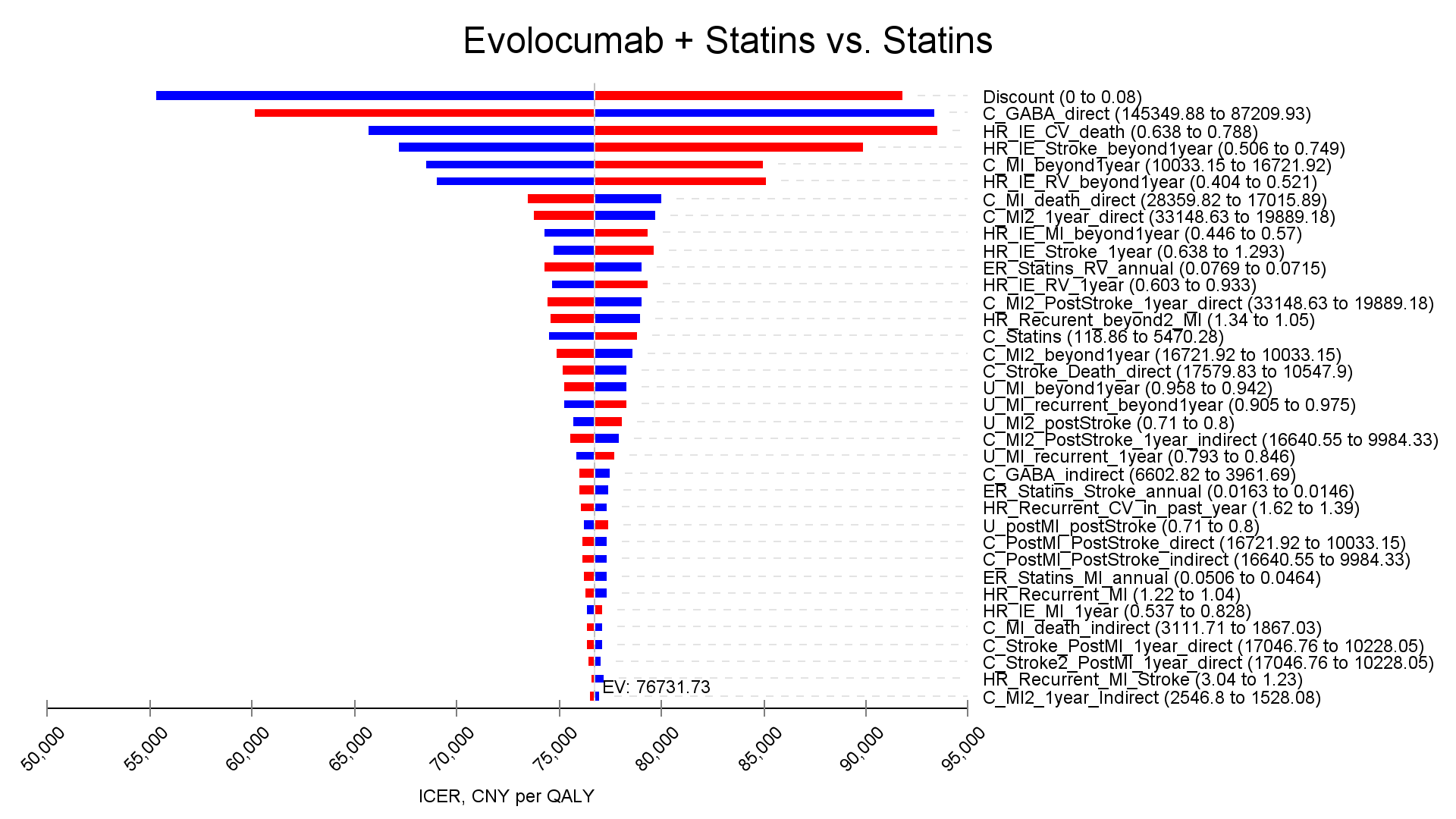


C evolocumab 140 mg Q2W D evolocumab 420 mg QM

Fig. S2 Tornado plots for one-way deterministic sensitivity analysis of the SuValue® database population with LDL-C levels ≥100 mg/dL

A and B are the results from the Chinese healthcare perspective, C and D are the results from the Chinese private payer perspective;

C_, Cost; CNY, Chinese yuan, CV: cardiovascular; ER_, event rate; EV, expected value; HR_, Hazard ratio; ICER, incremental cost-effectiveness ratio; IE_, Intervention effect; MI, myocardial infarction; RV_, revascularization; U_, Utility; WTP, willingness-to-pay.


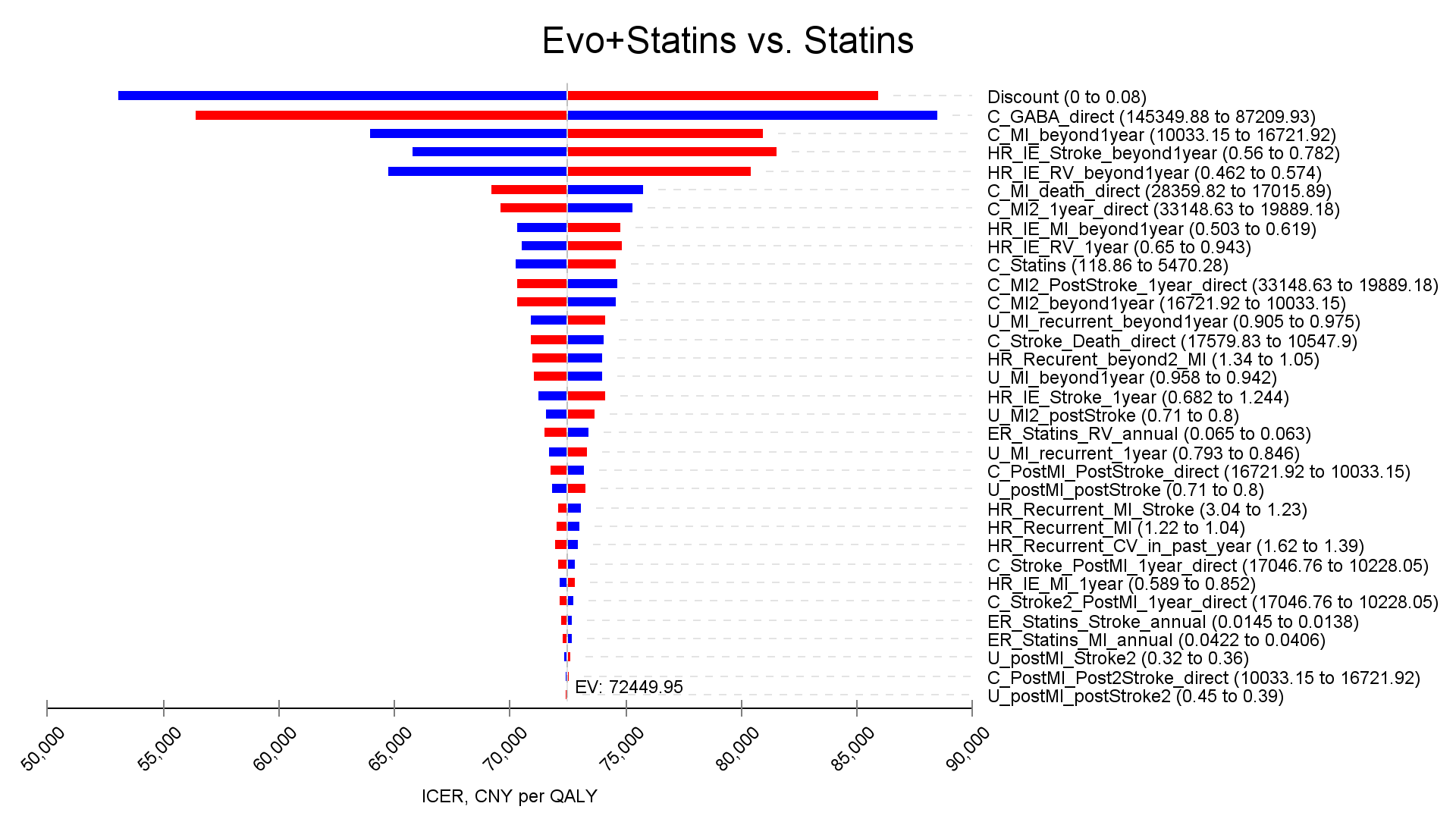

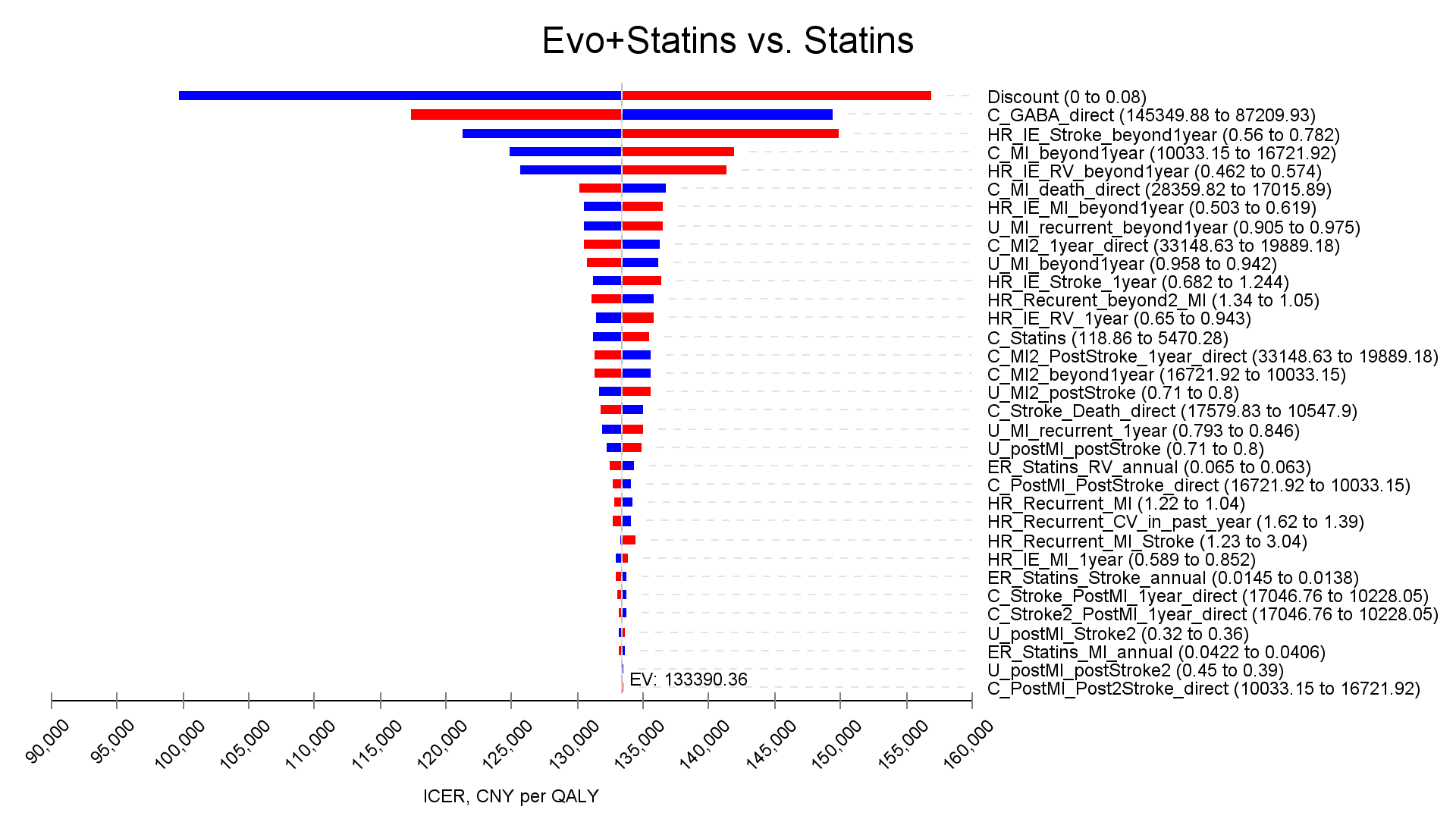


A evolocumab 140 mg Q2W B evolocumab 420 mg QM


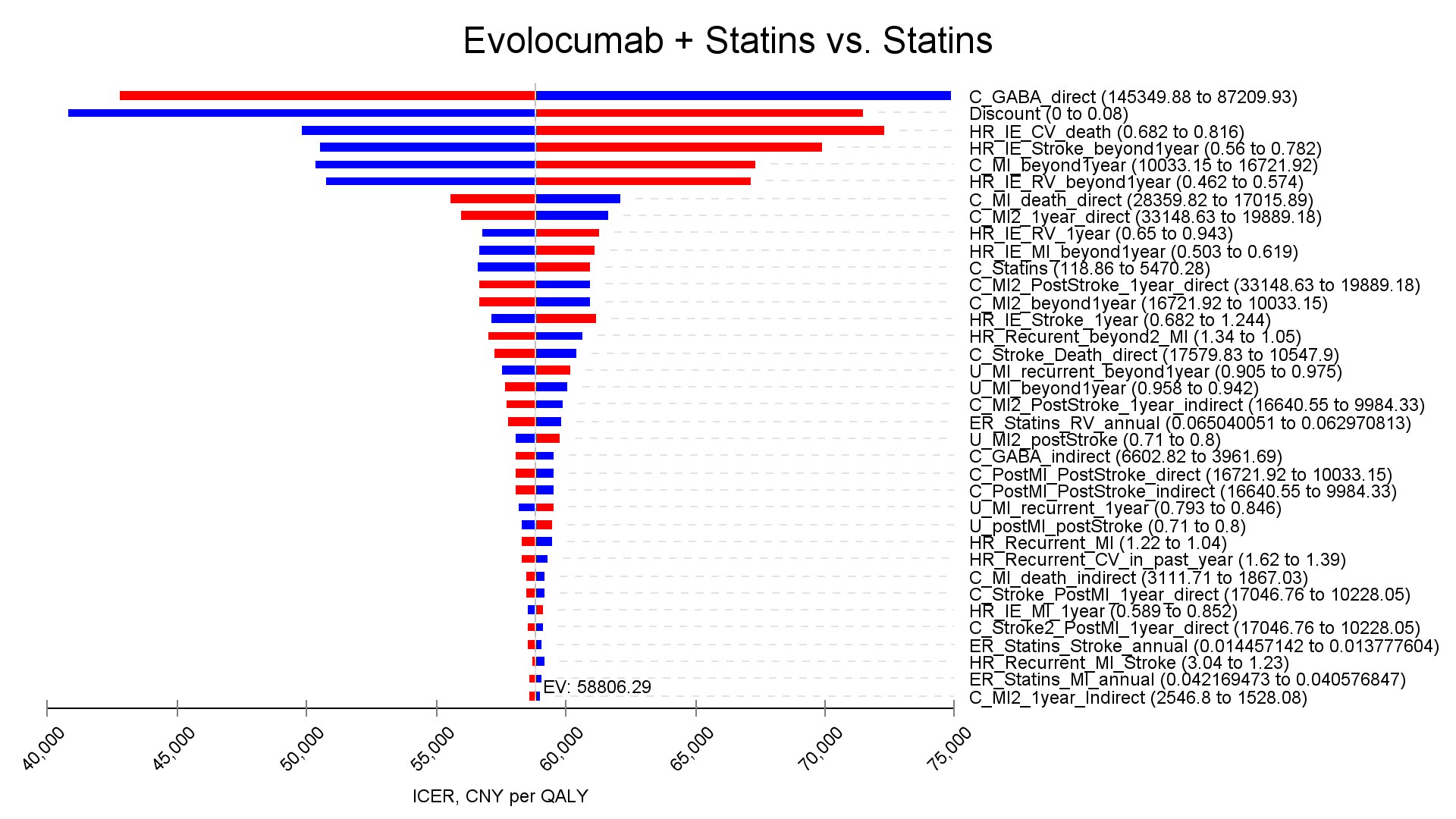

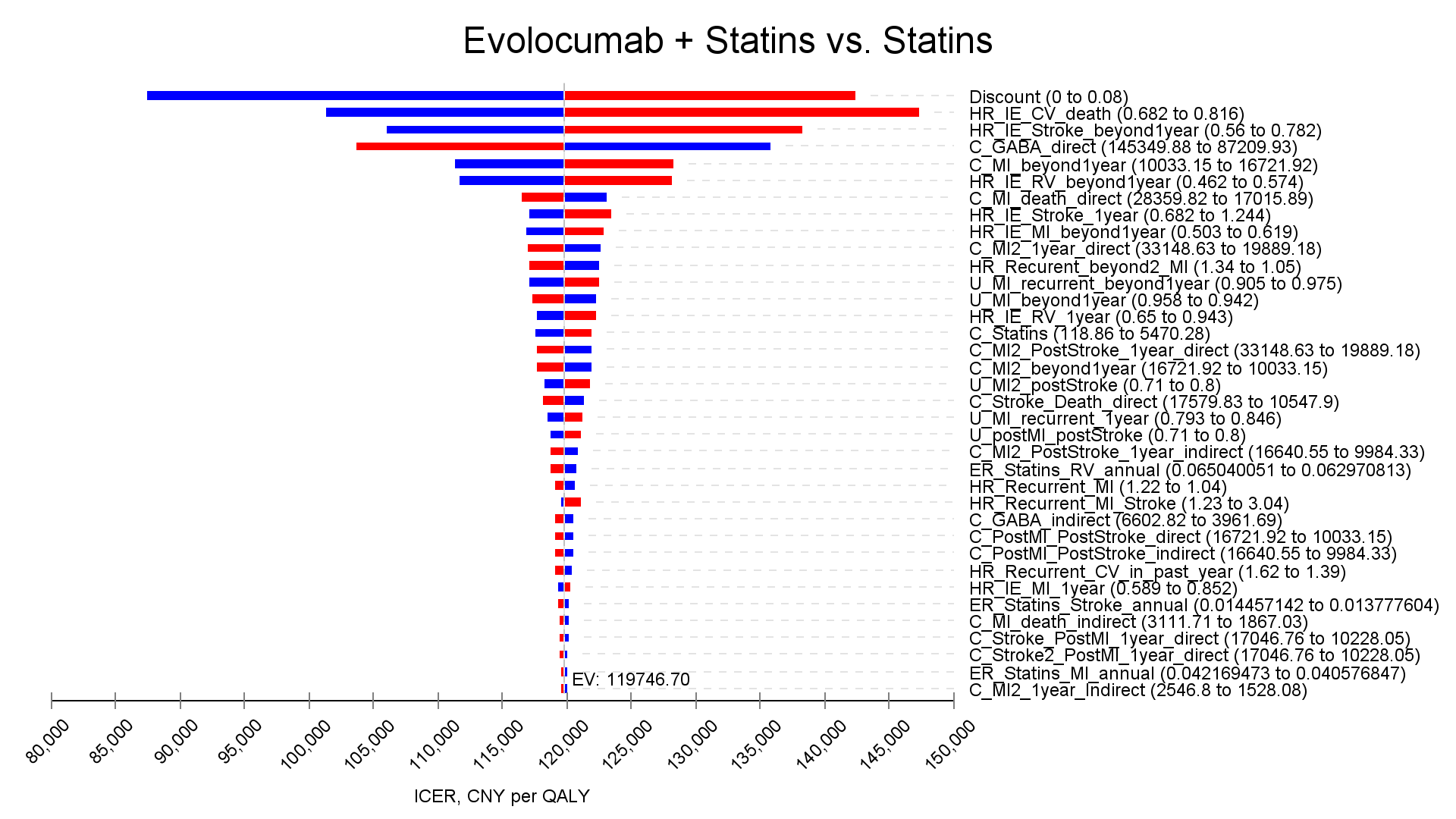


C evolocumab 140 mg Q2W D evolocumab 420 mg QM

Fig. S3 Tornado plots for one-way deterministic sensitivity analysis of the SuValue® database population with LDL-C levels ≥70 mg/dL

A and B are the results from the Chinese healthcare perspective, C and D are the results from the Chinese private payer perspective;

C_, Cost; CNY, Chinese yuan, CV: cardiovascular; ER_, event rate; EV, expected value; HR_, Hazard ratio; ICER, incremental cost-effectiveness ratio; IE_, Intervention effect; MI, myocardial infarction; RV_, revascularization; U_, Utility; WTP, willingness-to-pay.


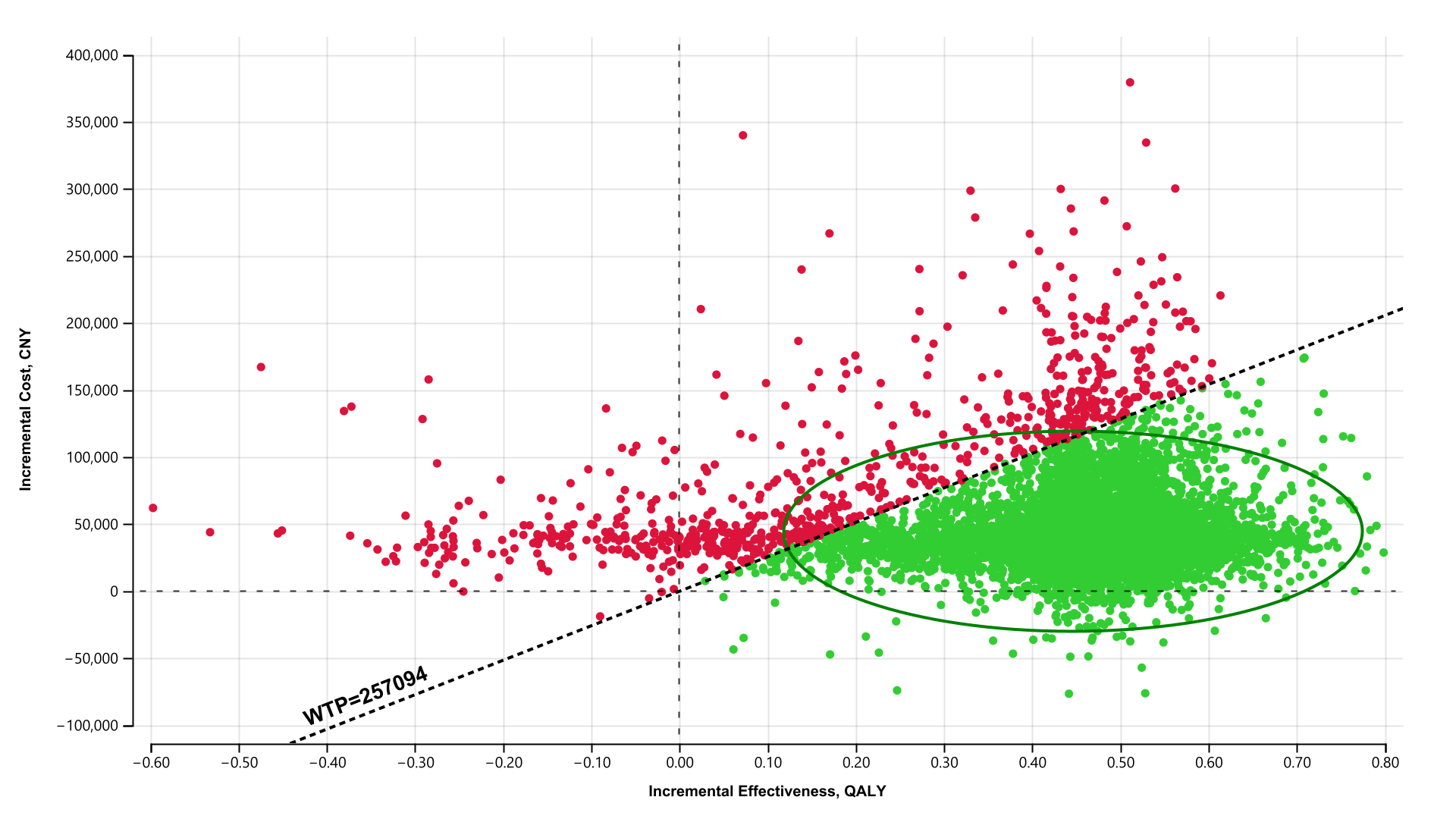

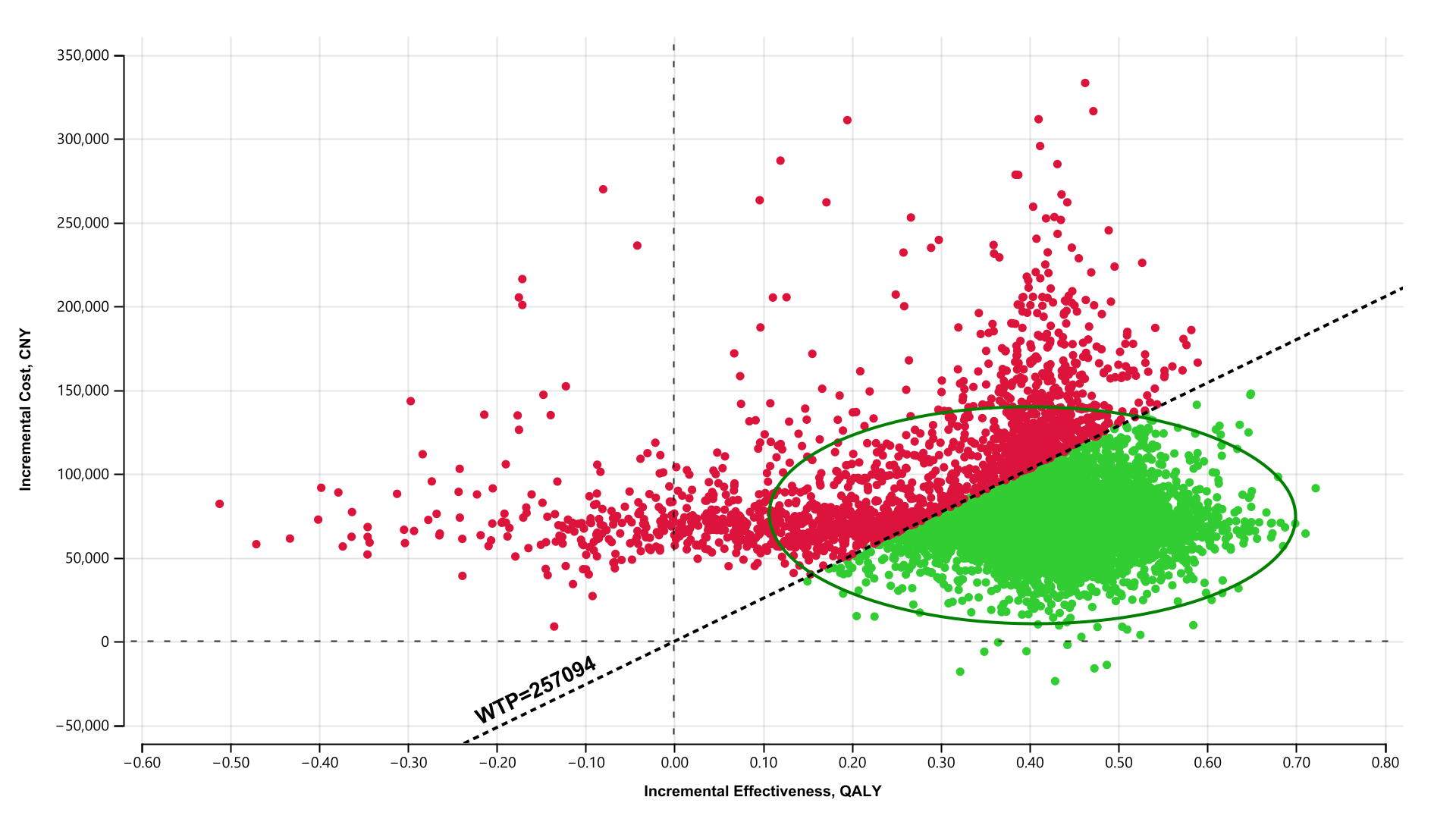


A evolocumab 140 mg Q2W B evolocumab 420 mg QM


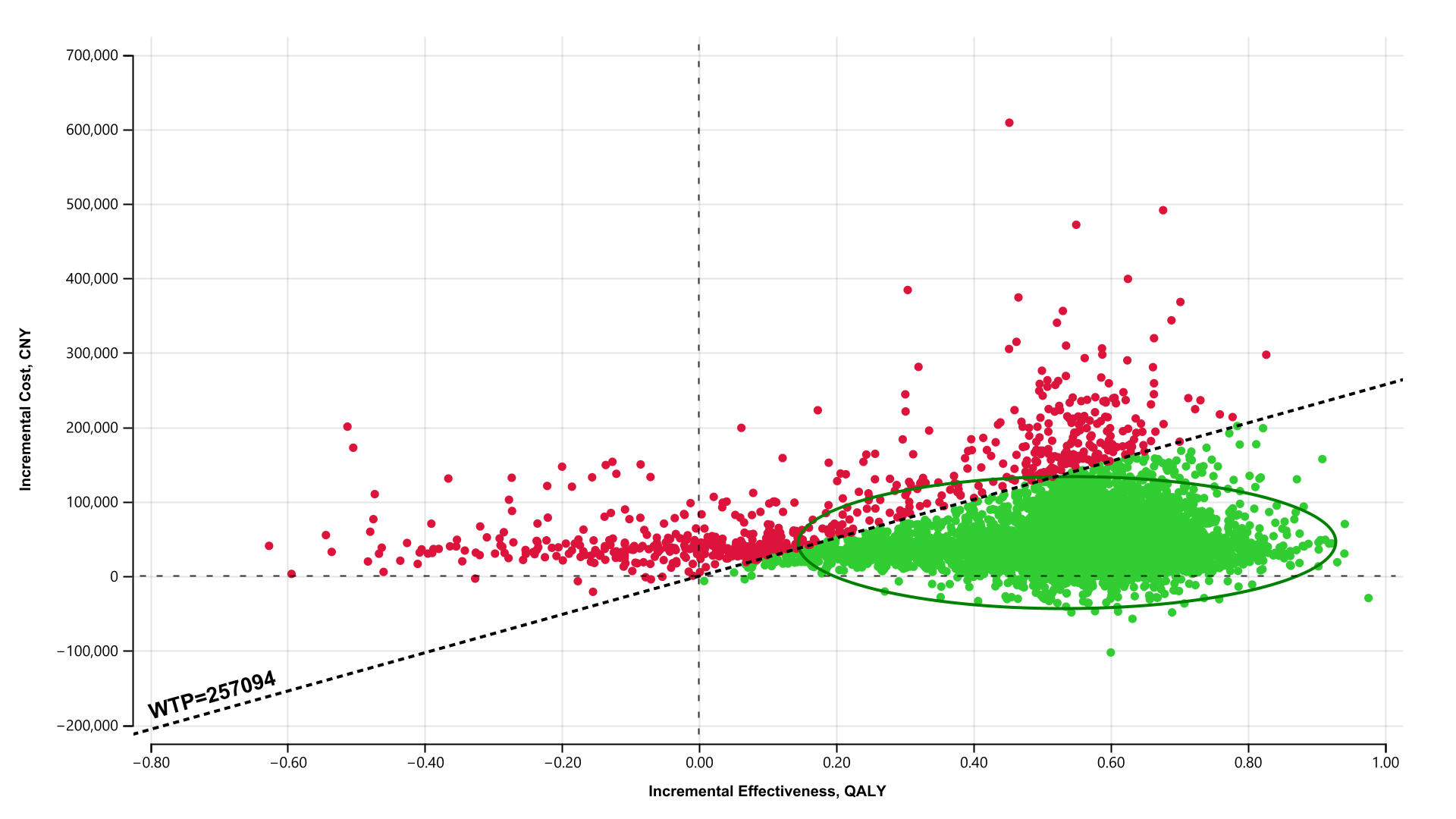

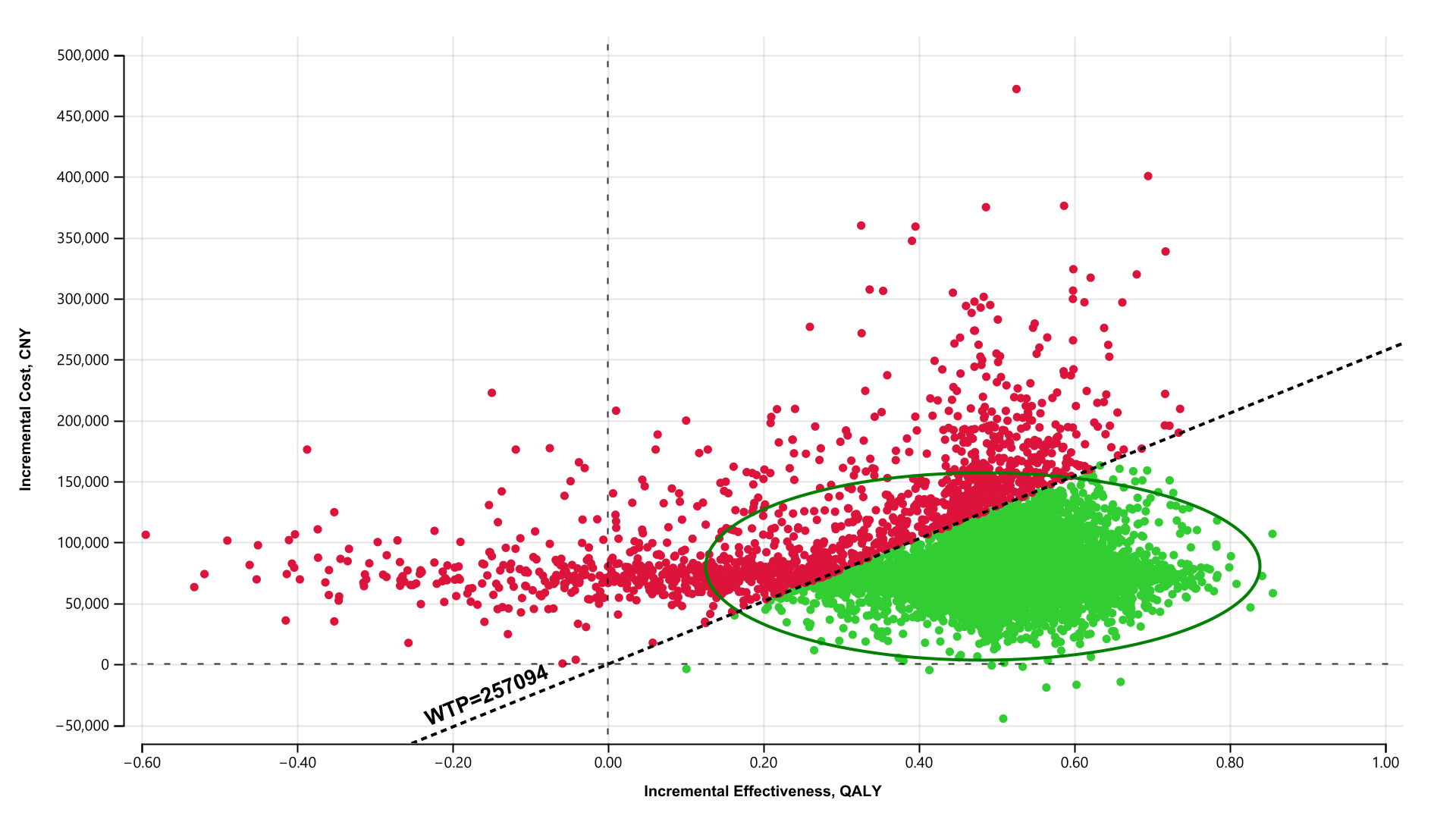


C evolocumab 140 mg Q2W D evolocumab 420 mg QM

Fig. S4 Monte Carlo simulation scatters plot in probabilistic sensitivity analyses of the BERSON study population.

A and B are the results from the Chinese healthcare perspective, C and D are the results from the Chinese private payer perspective;

The dotted line shows the willingness-to-pay threshold, with a slope of CNY 257,094 per quality-adjusted life-year gained. CNY, Chinese yuan; QALY, quality-adjusted life-year; WTP, willingness-to-pay.


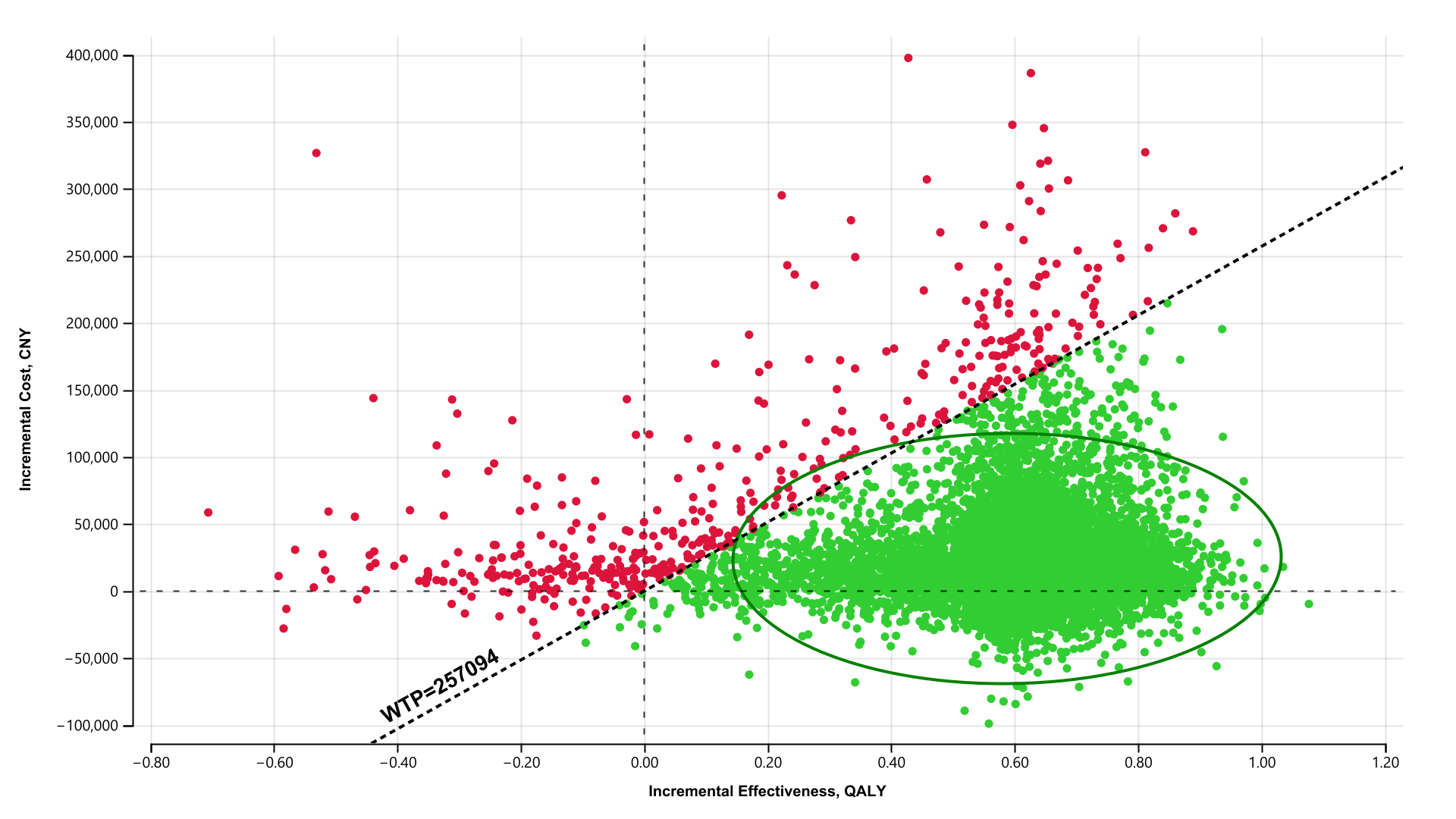

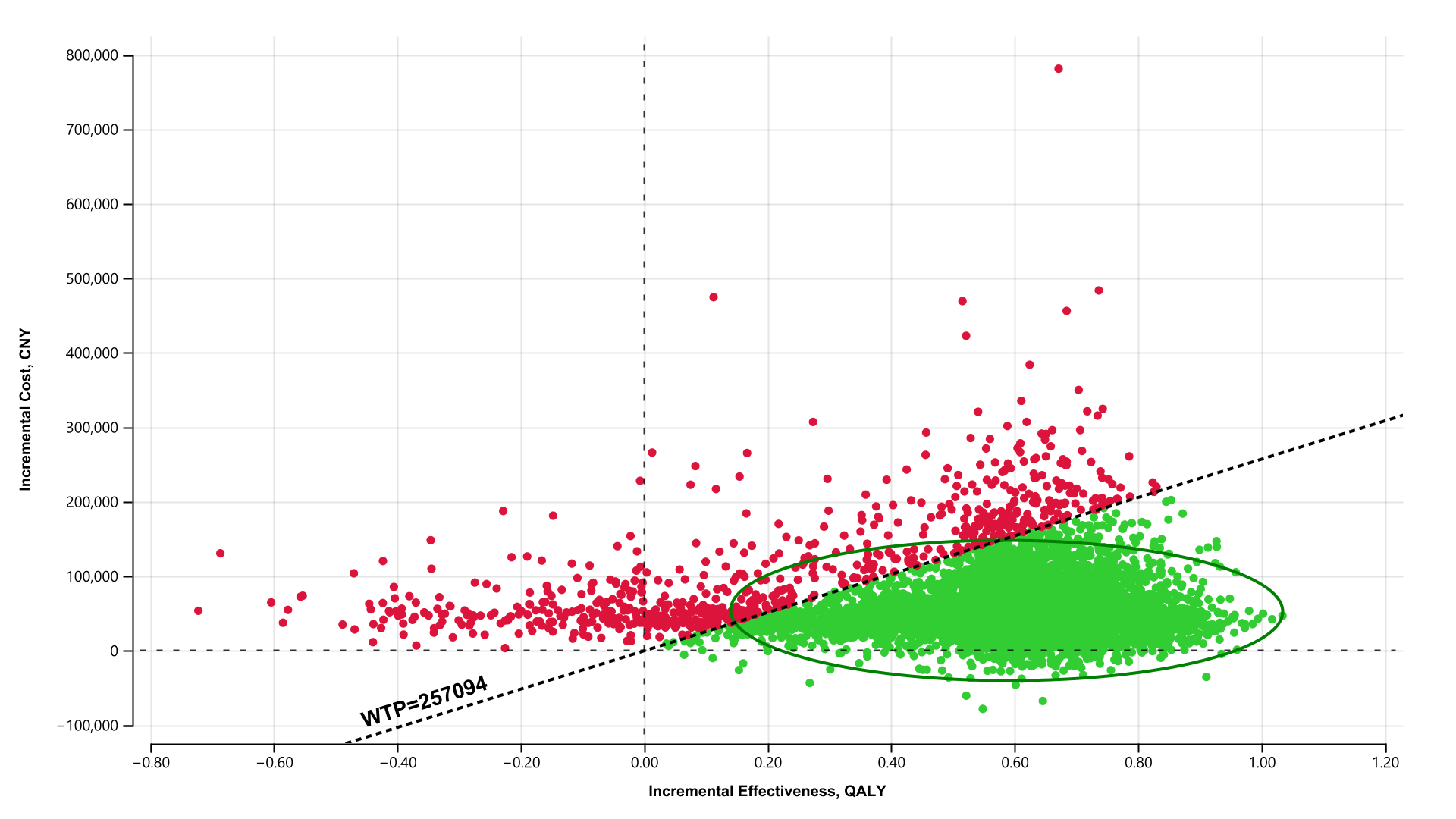


A evolocumab 140 mg Q2W B evolocumab 420 mg QM


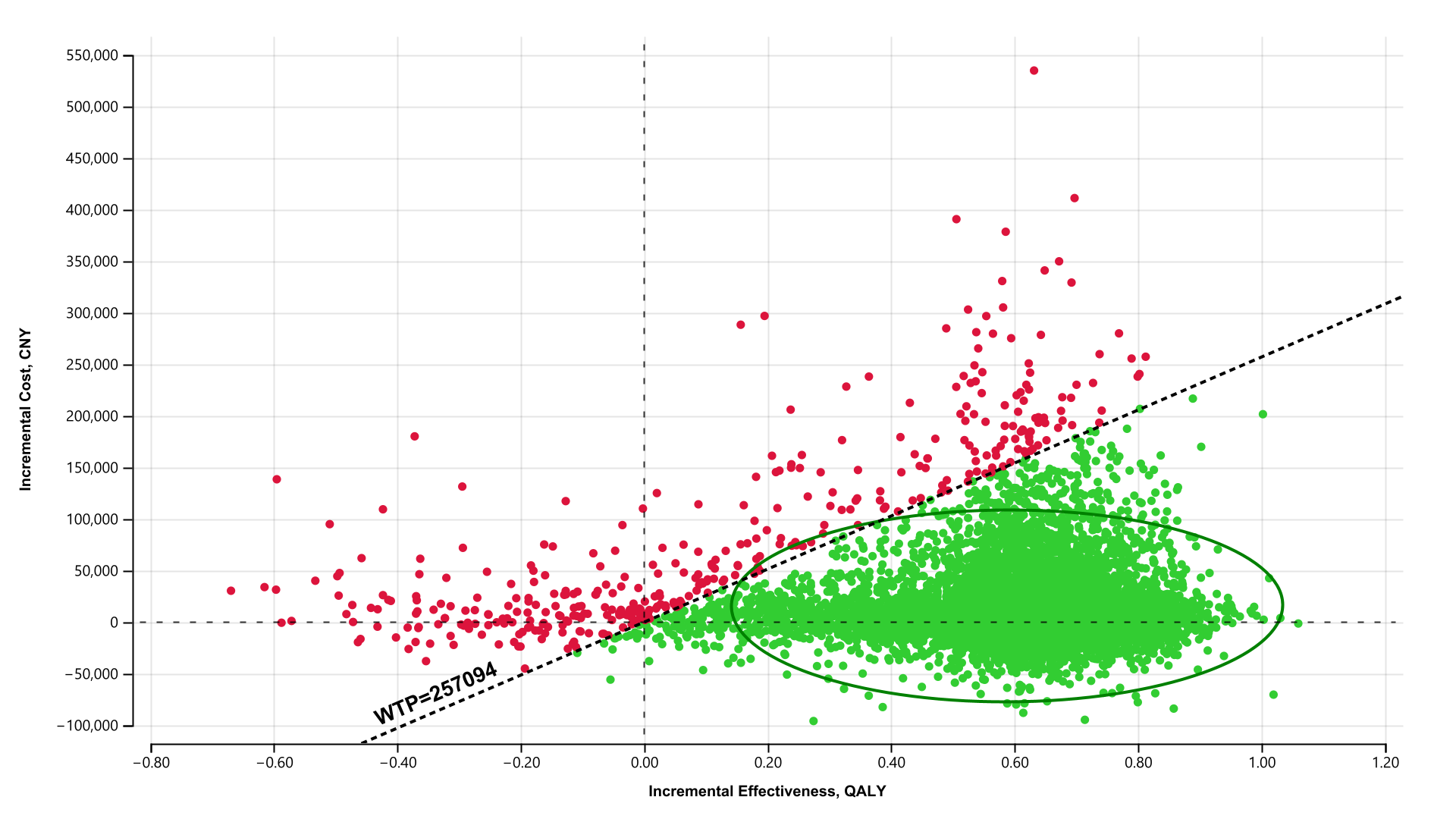

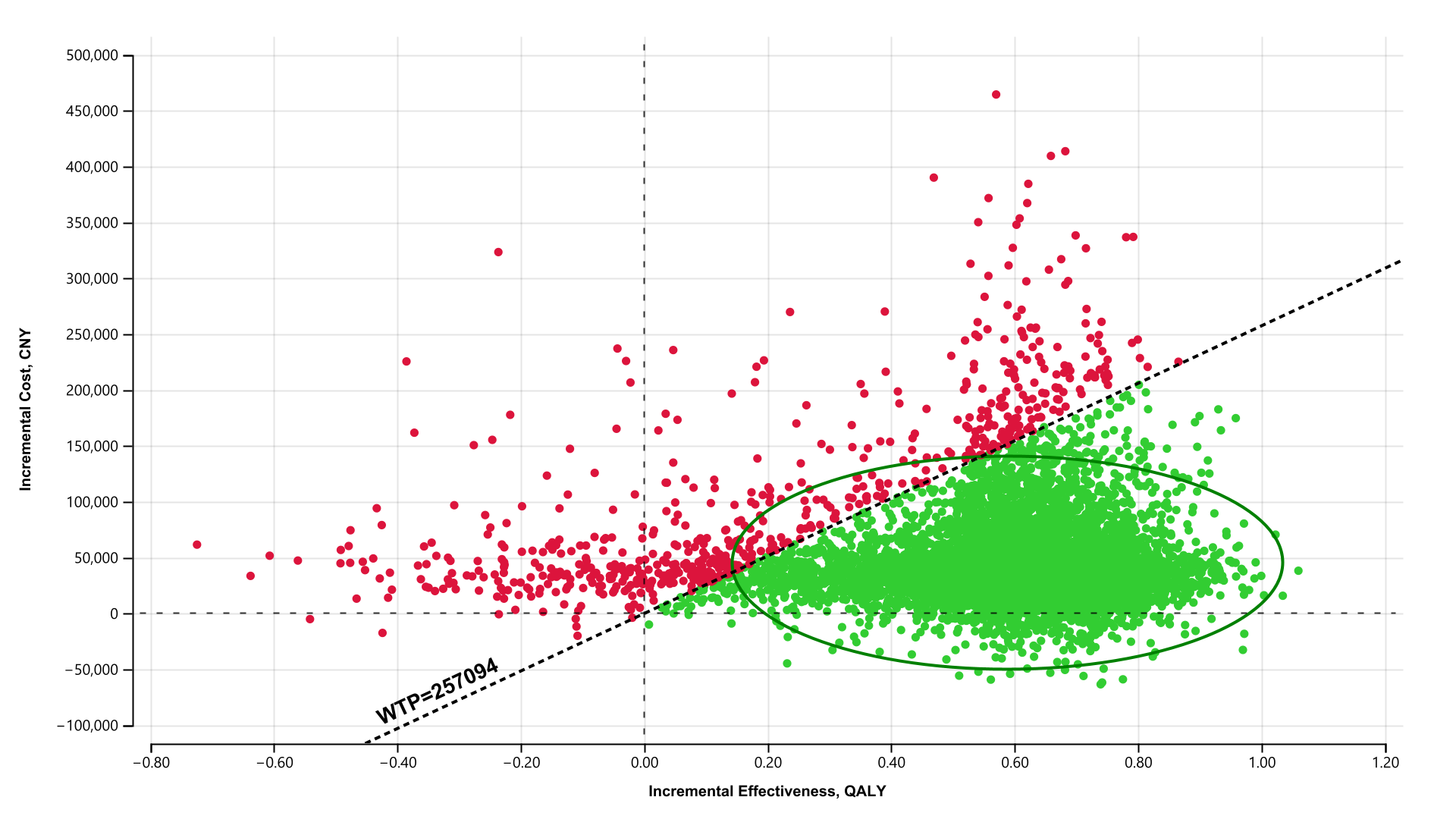


C evolocumab 140 mg Q2W D evolocumab 420 mg QM

Figure S5 Monte Carlo simulation scatters plot in probabilistic sensitivity analyses of the SuValue® database population with LDL-C levels ≥100 mg/dL.

A and B are the results from the Chinese healthcare perspective, C and D are the results from the Chinese private payer perspective;

The dotted line shows the willingness-to-pay threshold, with a slope of CNY 257,094 per quality-adjusted life-year gained. CNY, Chinese yuan; QALY, quality-adjusted life-year; WTP, willingness-to-pay.


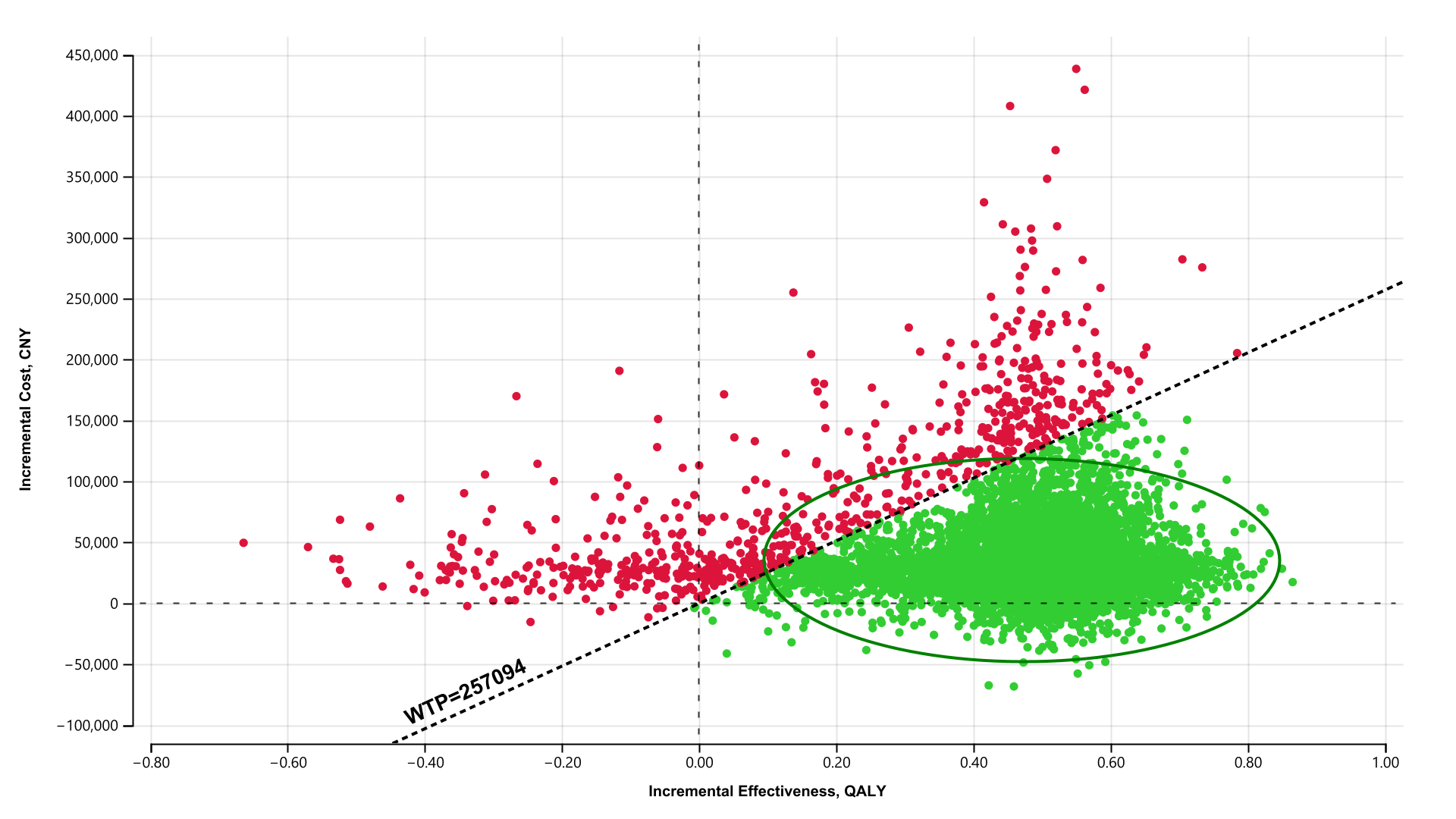

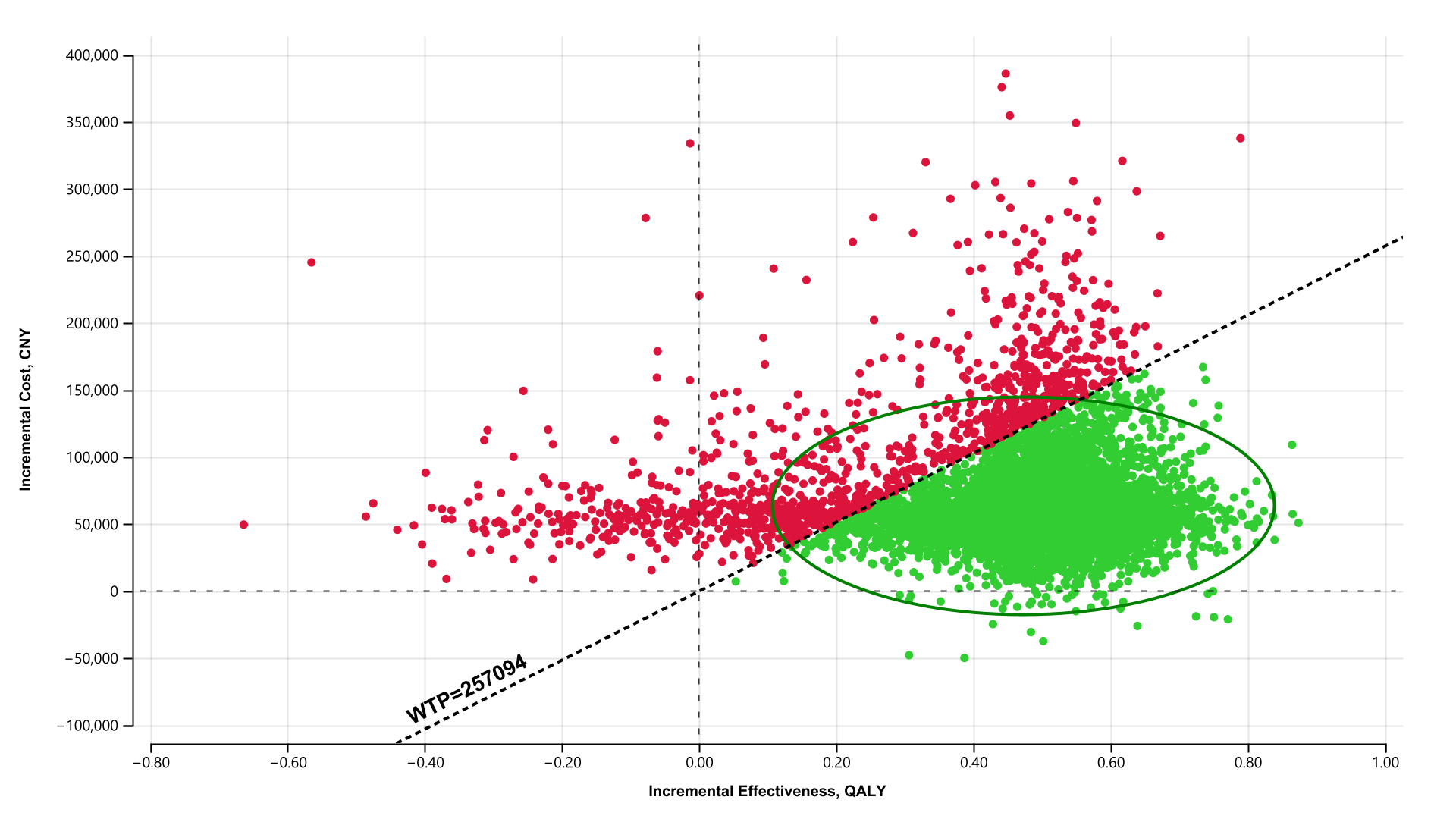


A evolocumab 140 mg Q2W B evolocumab 420 mg QM


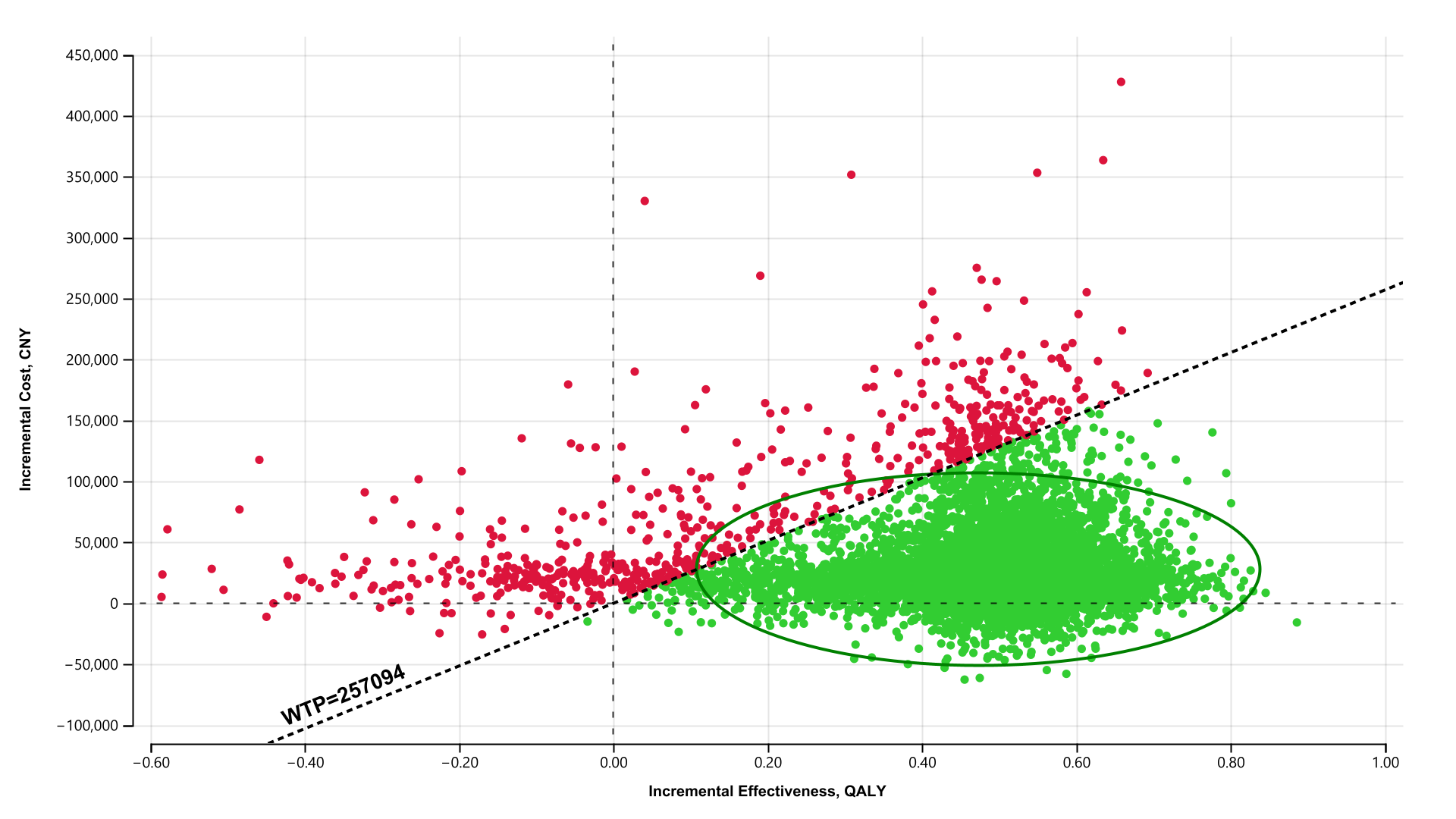

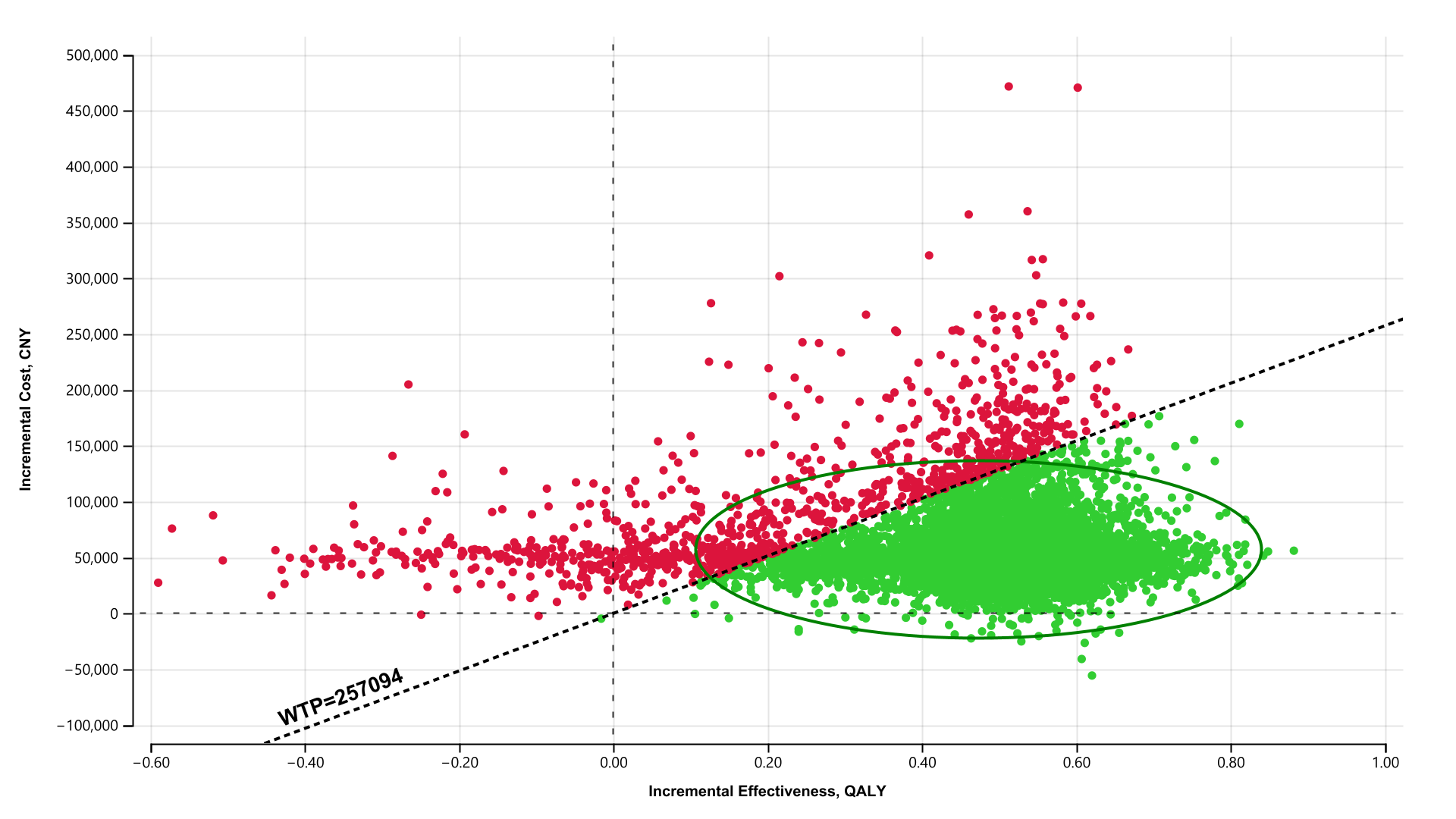


C evolocumab 140 mg Q2W D evolocumab 420 mg QM

Figure S6 Monte Carlo simulation scatters plot in probabilistic sensitivity analyses of the SuValue® database population with LDL-C levels ≥70 mg/dL.

A and B are the results from the Chinese healthcare perspective, C and D are the results from the Chinese private payer perspective;

The dotted line shows the willingness-to-pay threshold, with a slope of CNY 257,094 per quality-adjusted life-year gained. CNY, Chinese yuan; QALY, quality-adjusted life-year; WTP, willingness-to-pay.


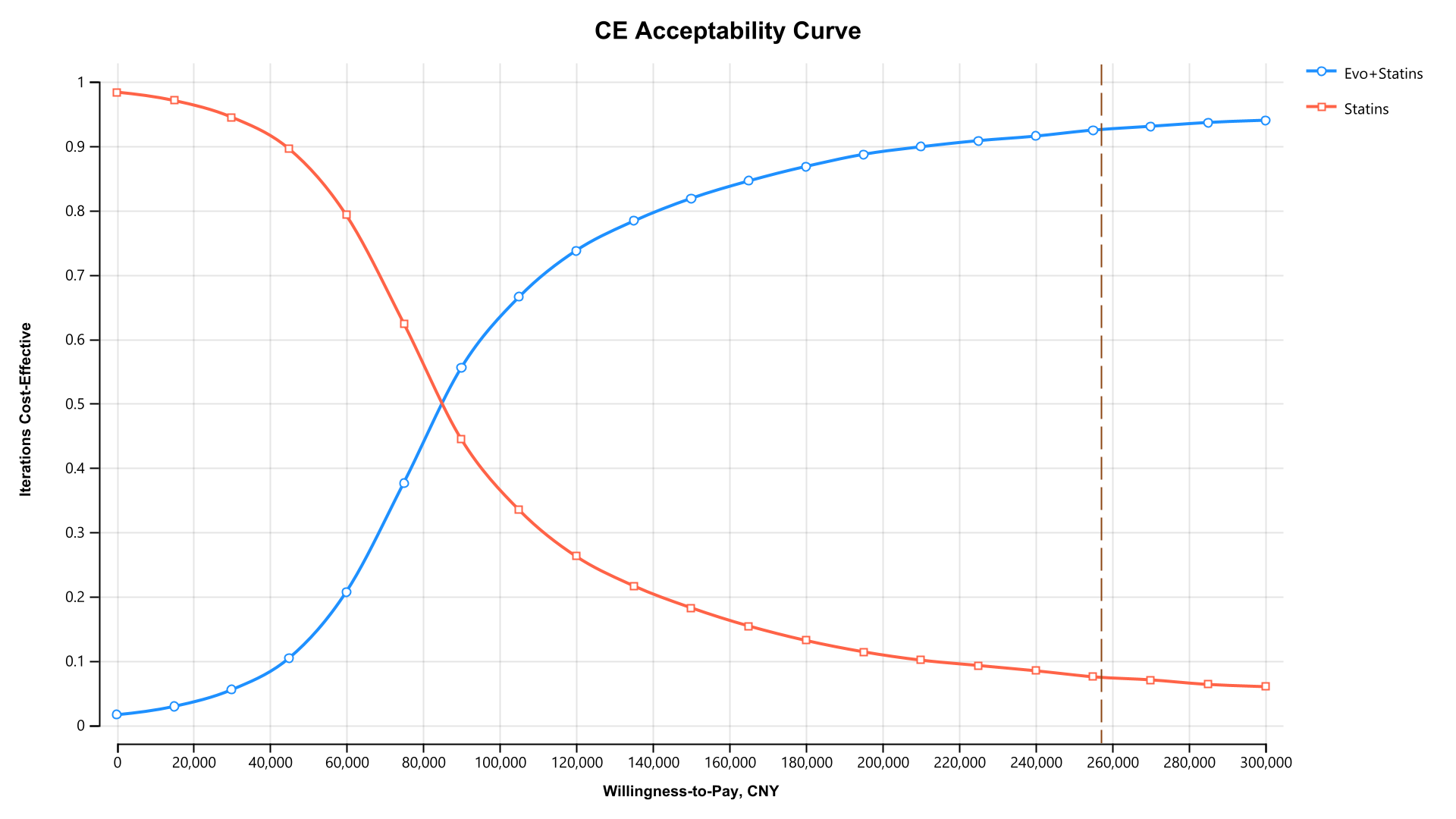

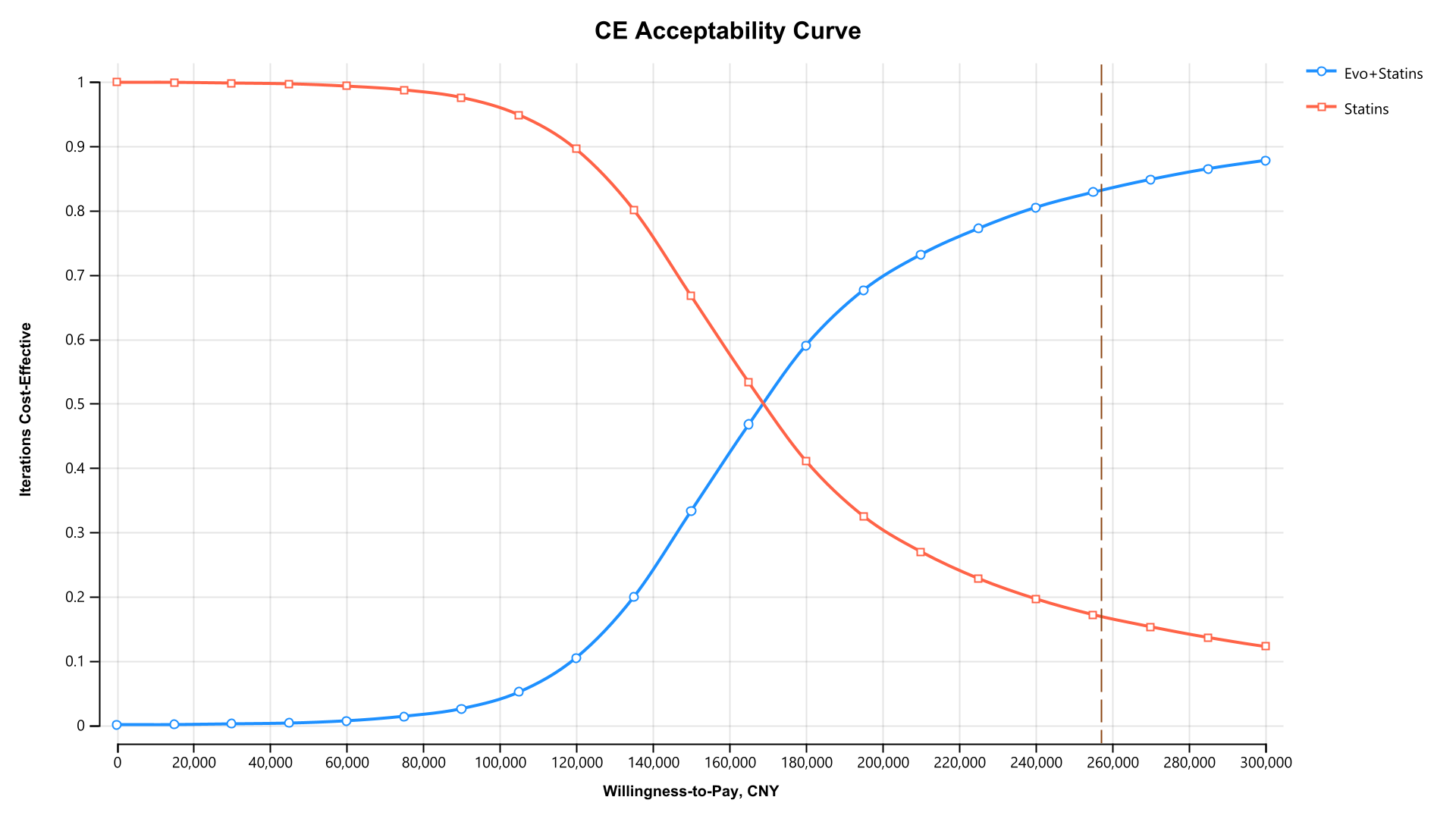


A evolocumab 140 mg Q2W B evolocumab 420 mg QM


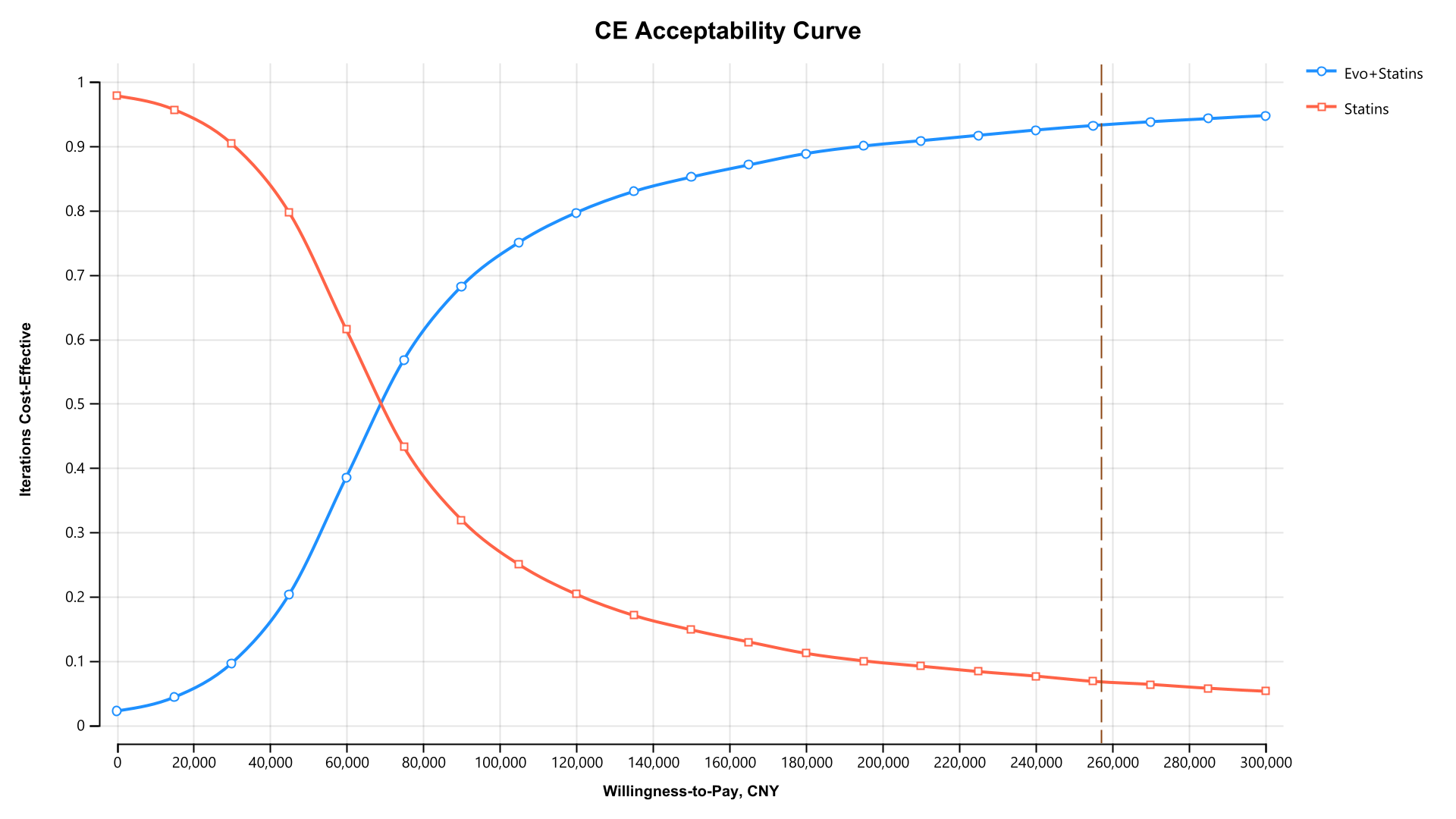

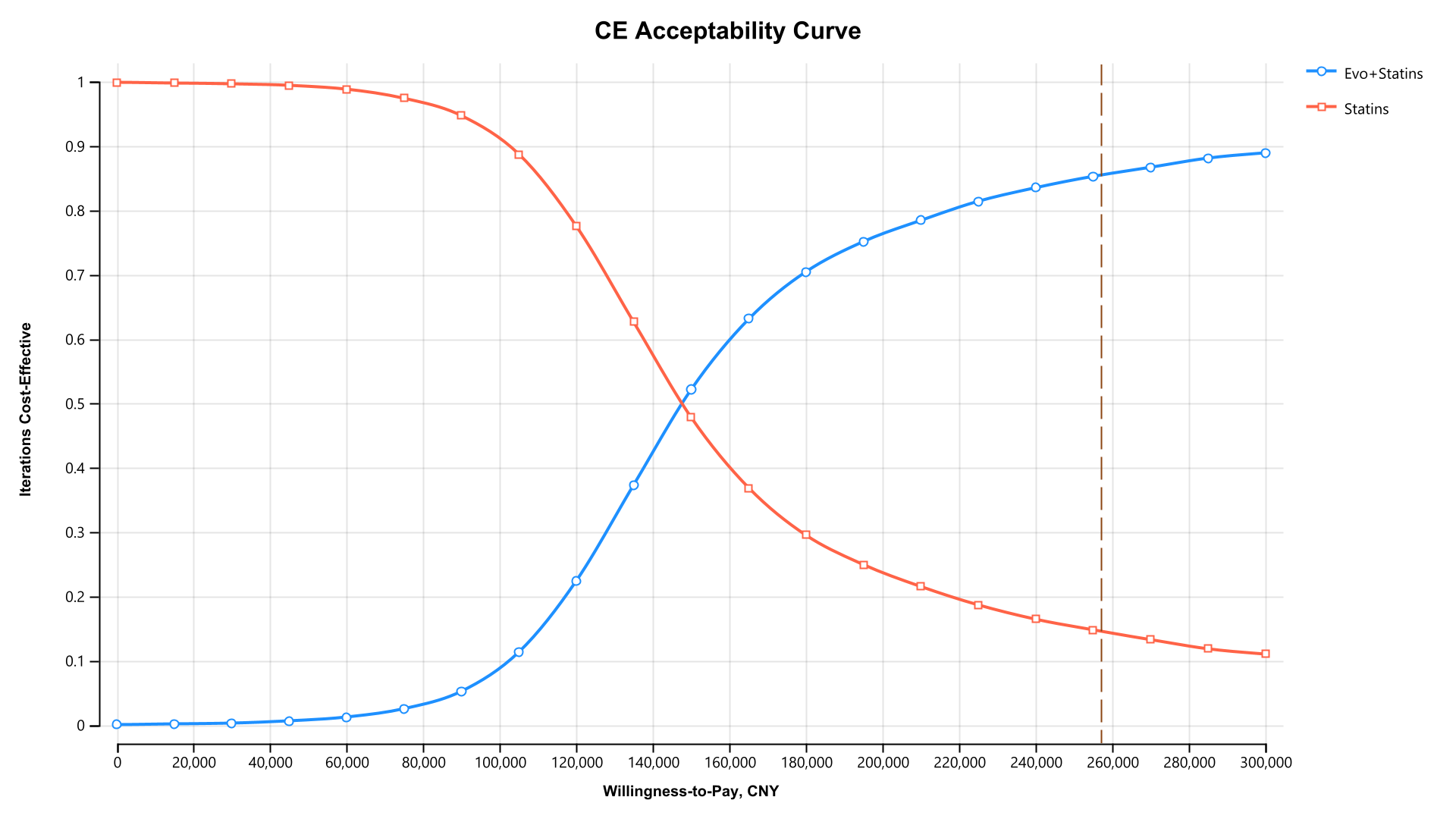


C evolocumab 140 mg Q2W D evolocumab 420 mg QM

Figure S7 Cost-effectiveness acceptability curves in probabilistic sensitivity analyses of the BERSON study population.

A and B are the results from the Chinese healthcare perspective, C and D are the results from the Chinese private payer perspective;

The dashed line shows the willingness-to-pay threshold of CNY 257,094 per quality-adjusted life-year gained. CNY, Chinese yuan; Evo, evolocumab; QALY, quality-adjusted life-year.


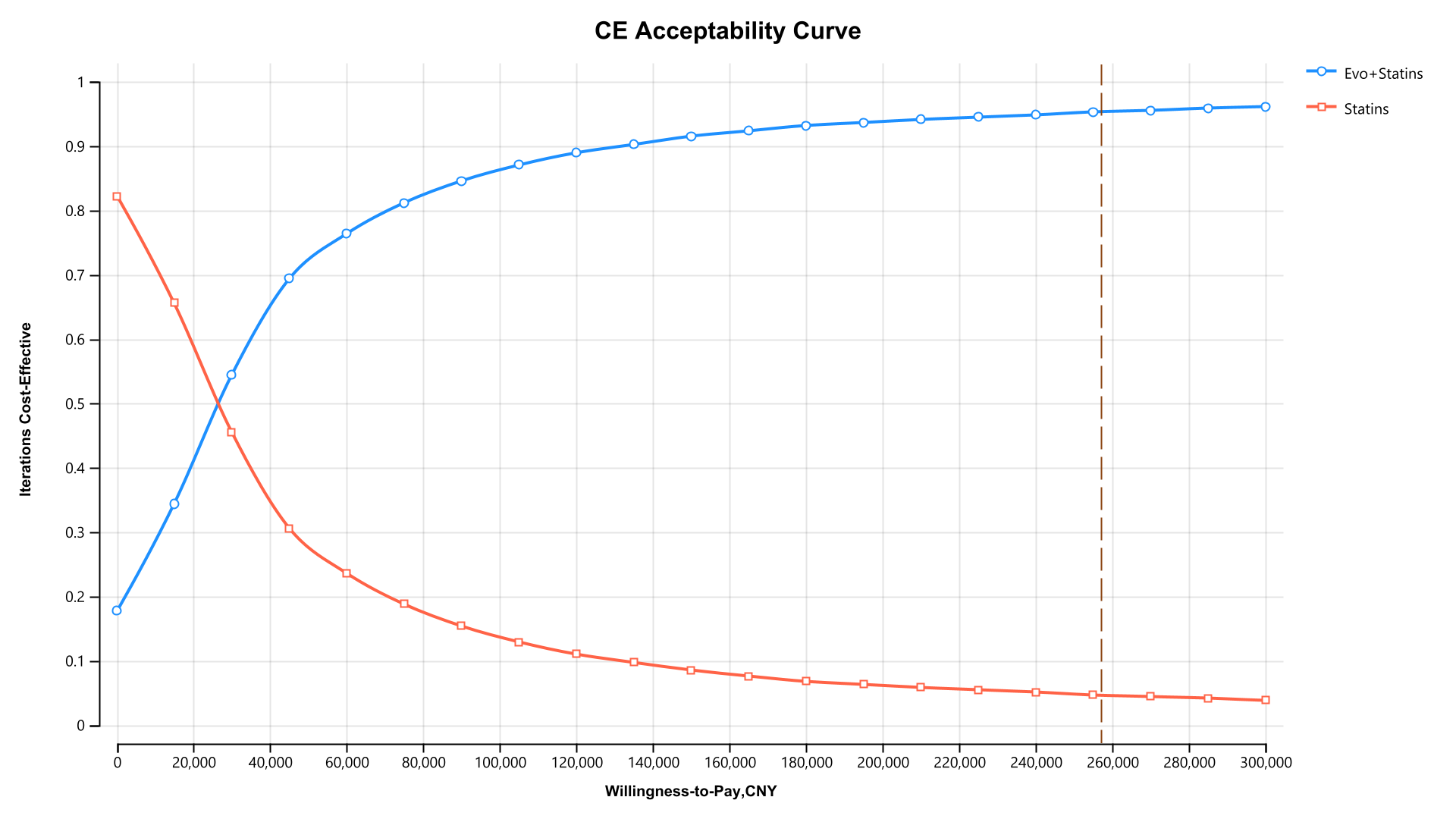

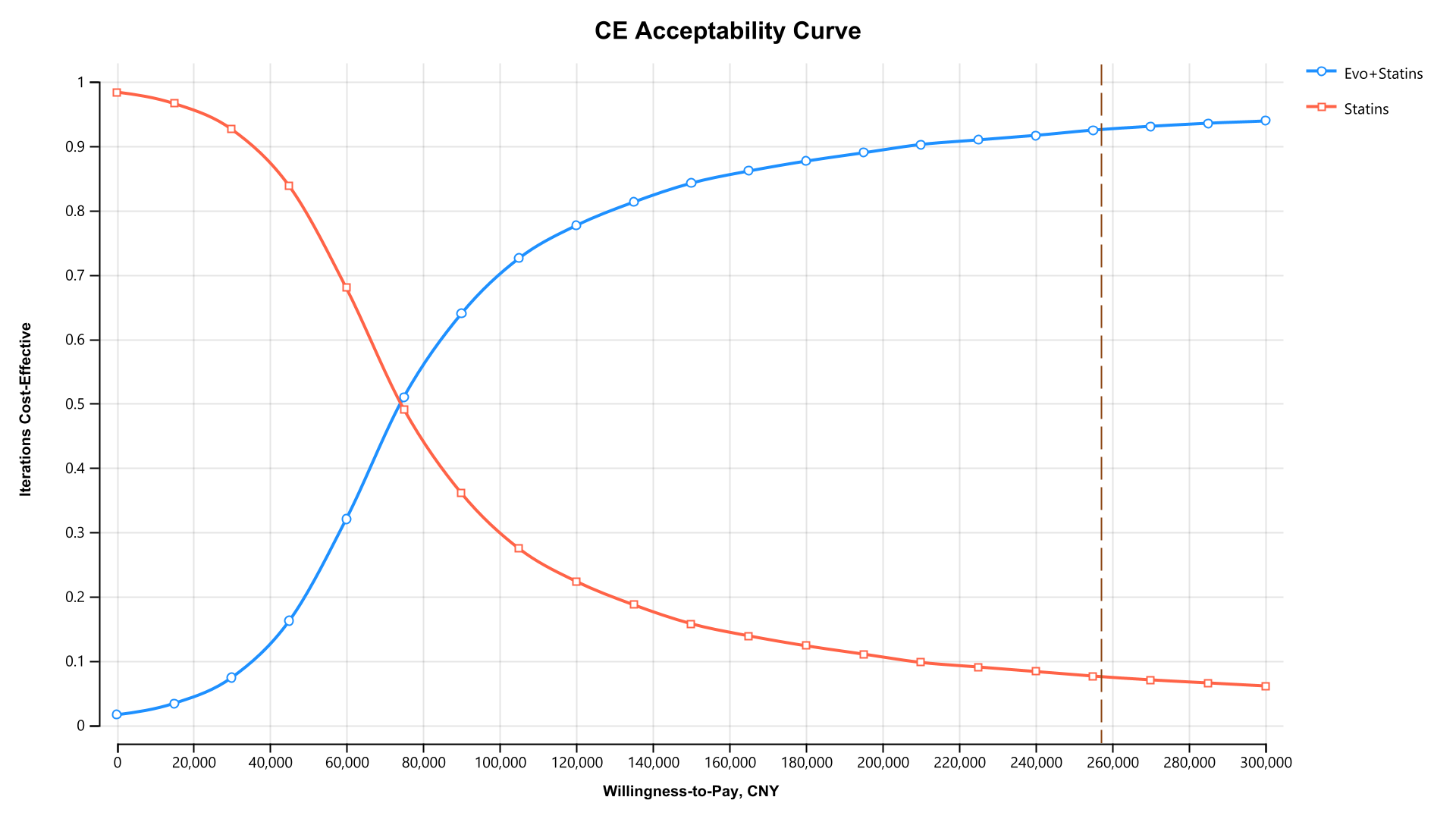


A evolocumab 140 mg Q2W B evolocumab 420 mg QM


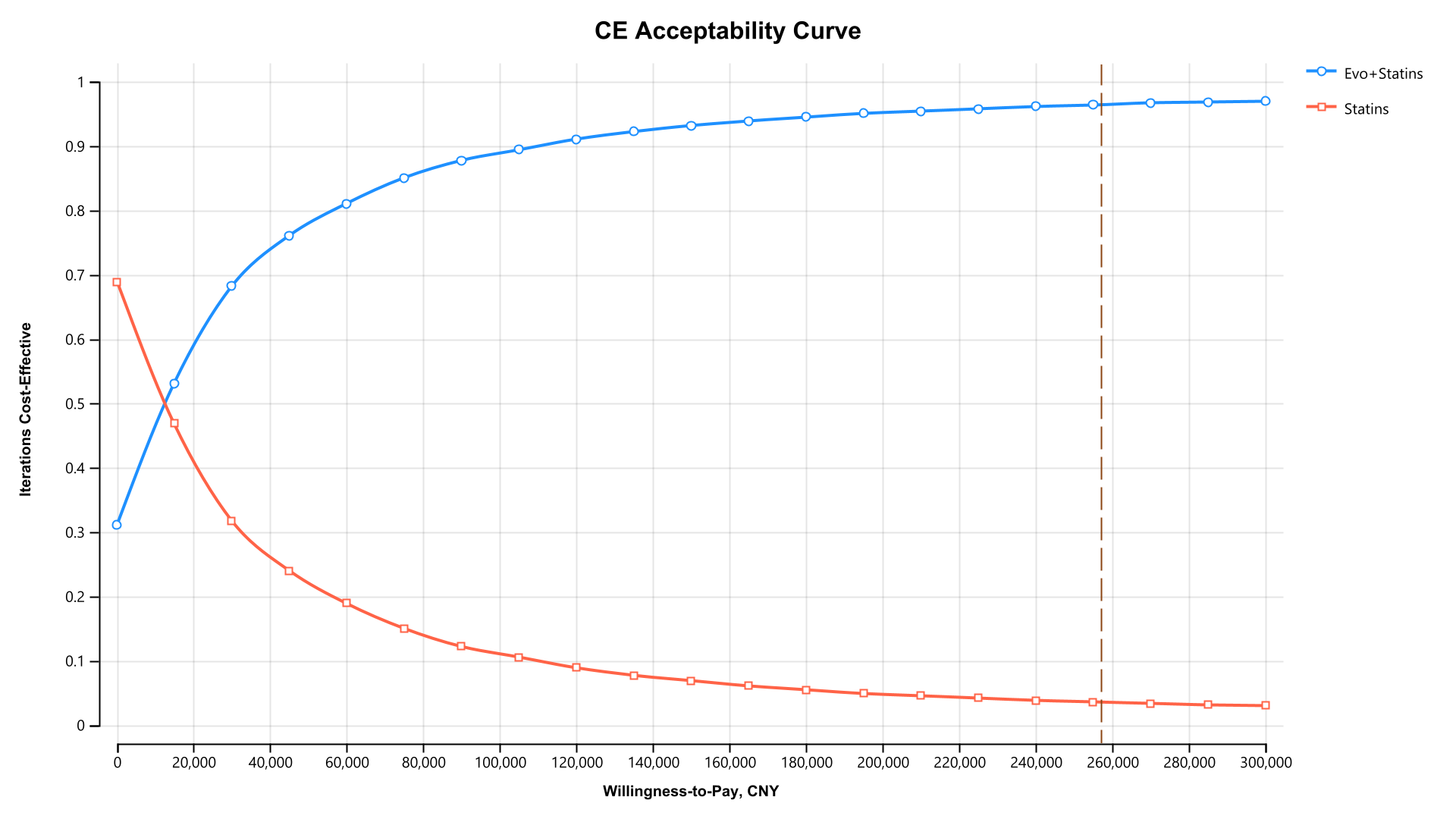

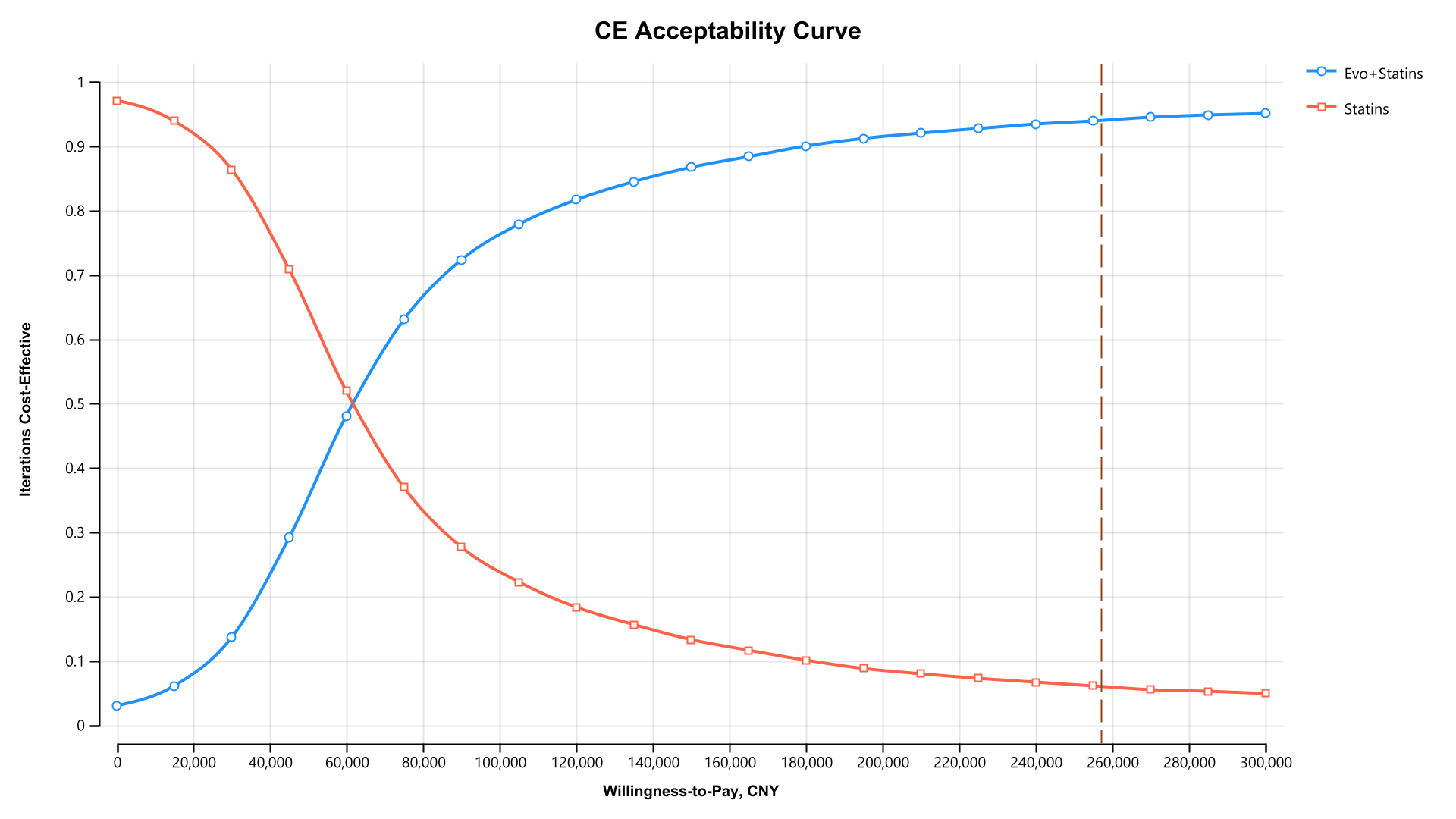


C evolocumab 140 mg Q2W D evolocumab 420 mg QM

Figure S8 Cost-effectiveness acceptability curves in probabilistic sensitivity analyses of the SuValue® database population with LDL-C levels ≥100 mg/dL.

A and B are the results from the Chinese healthcare perspective, C and D are the results from the Chinese private payer perspective;

The dashed line shows the willingness-to-pay threshold of CNY 257,094 per quality-adjusted life-year gained. CNY, Chinese yuan; Evo, evolocumab; QALY, quality-adjusted life-year.


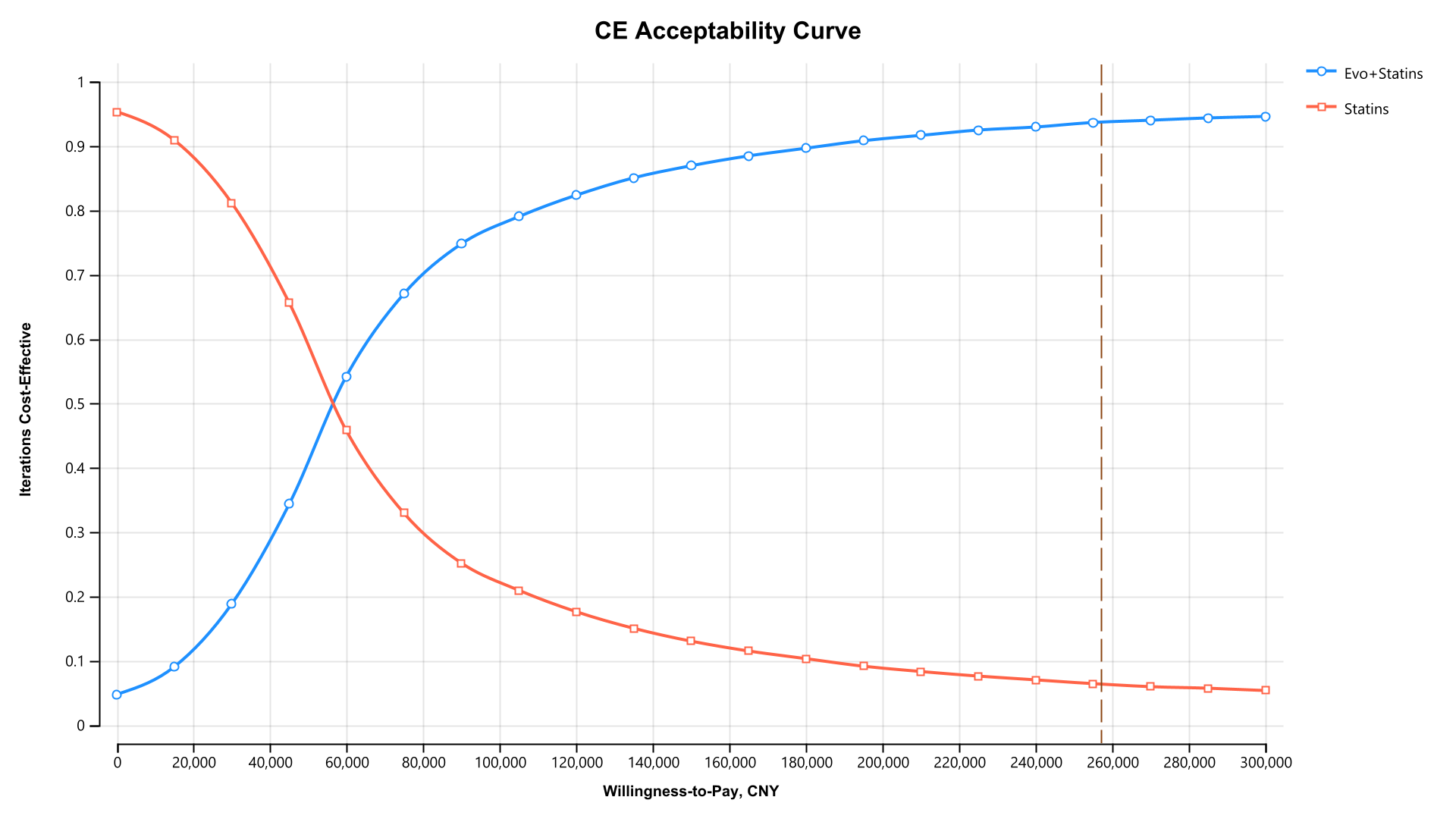

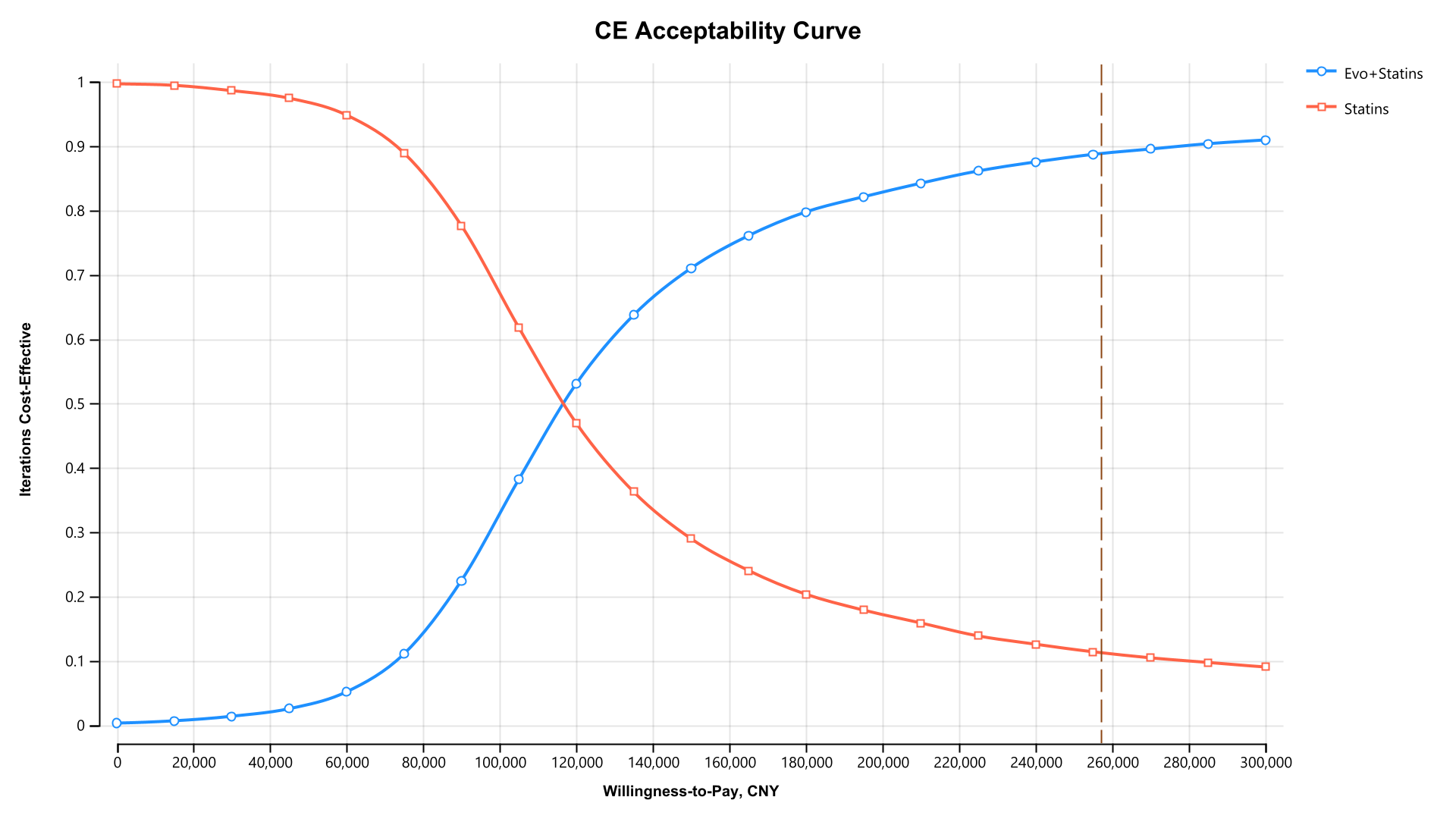


A evolocumab 140 mg Q2W B evolocumab 420 mg QM


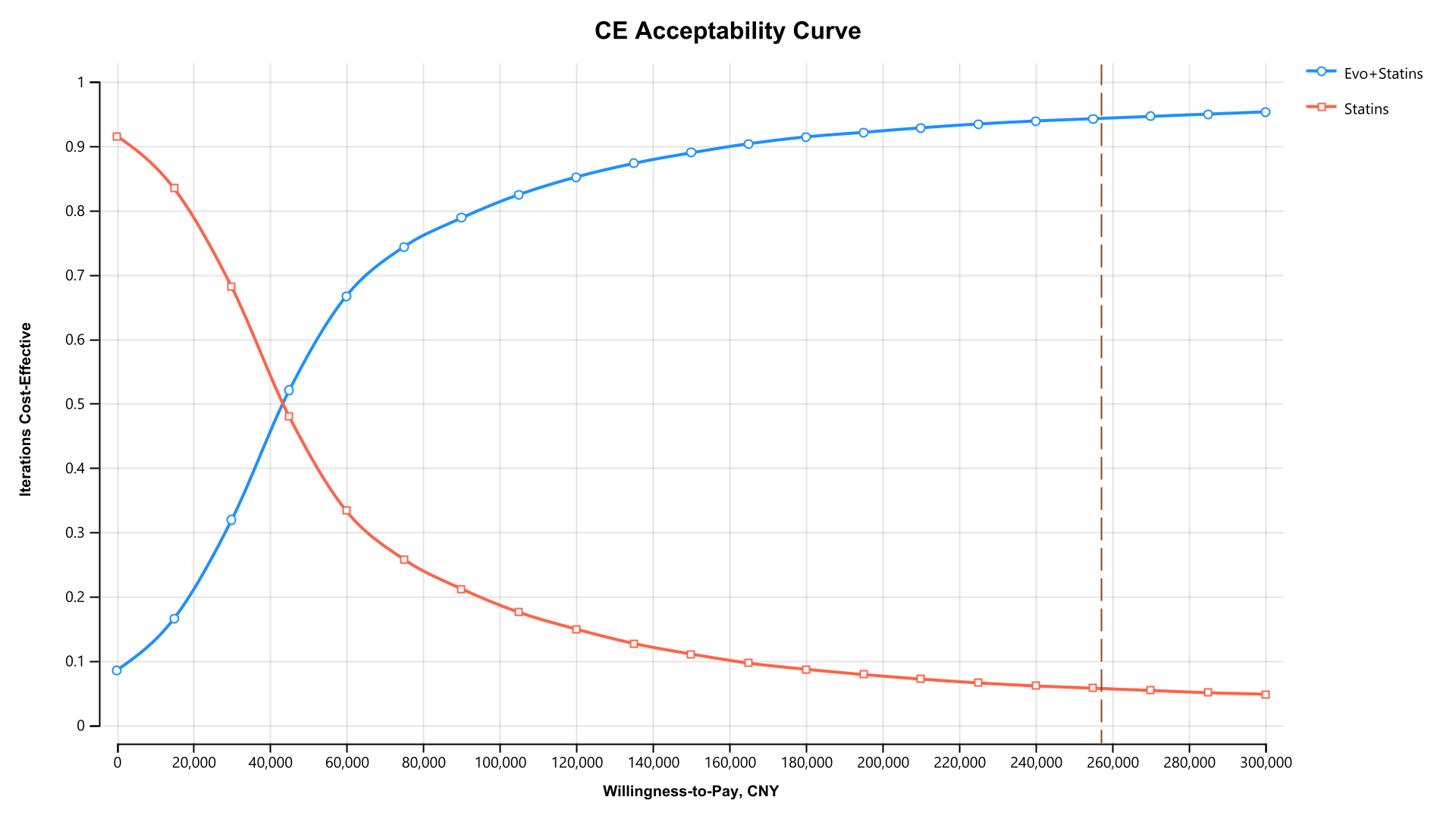

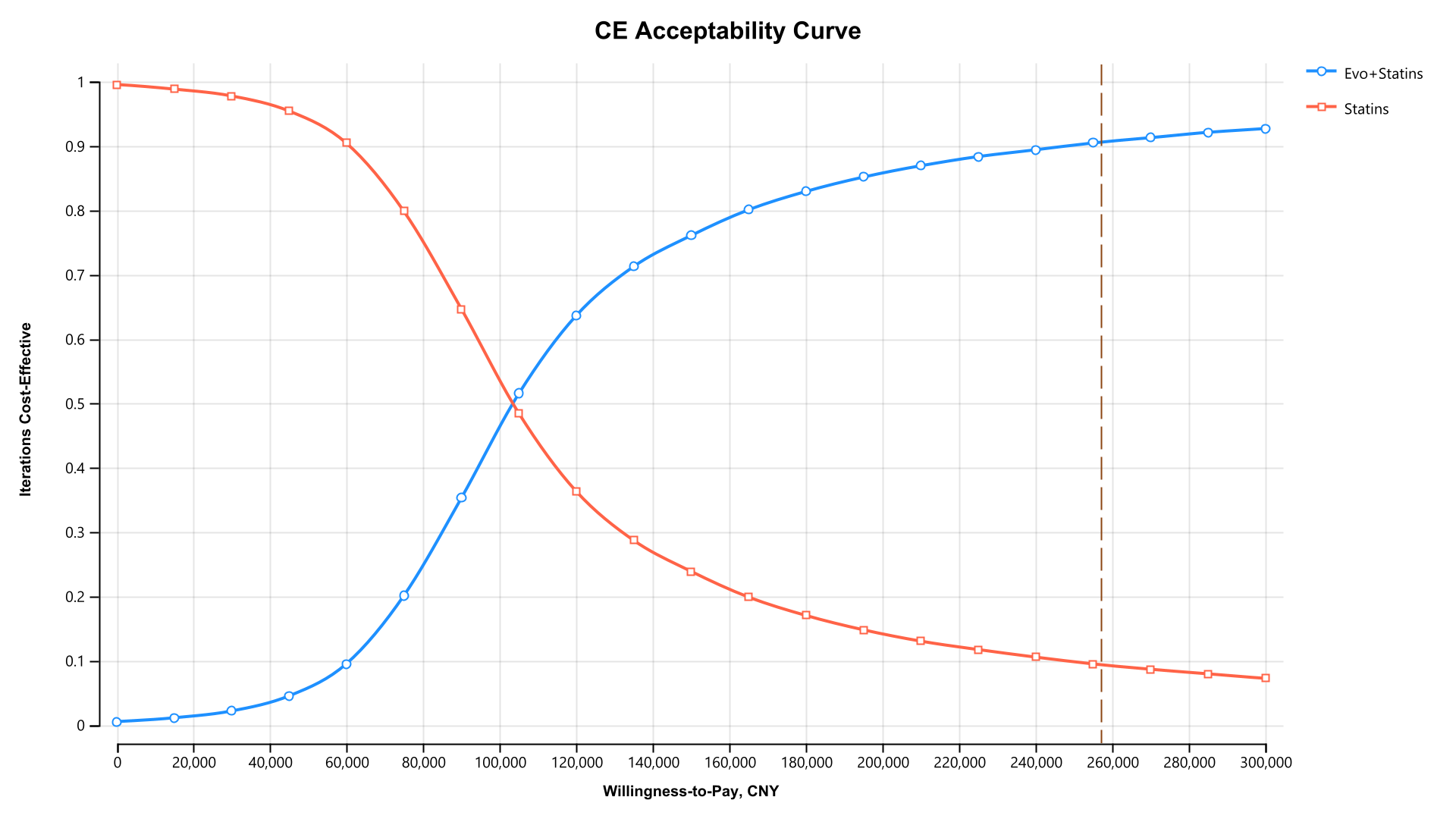


C evolocumab 140 mg Q2W D evolocumab 420 mg QM

Figure S9 Cost-effectiveness acceptability curves in probabilistic sensitivity analyses of the SuValue® database population with LDL-C levels ≥70 mg/dL.

A and B are the results from the Chinese healthcare perspective, C and D are the results from the Chinese private payer perspective;

The dashed line shows the willingness-to-pay threshold of CNY 257,094 per quality-adjusted life-year gained. CNY, Chinese yuan; Evo, evolocumab; QALY, quality-adjusted life-year.

**Reference**

1. Husereau D., Drummond M., Augustovski F., et al. Consolidated Health Economic Evaluation Reporting Standards (CHEERS) 2022 Explanation and Elaboration: A Report of the ISPOR CHEERS II Good Practices Task Force. Value Health. 2022;25(1):10-31. https://doi.org/ 10.1016/j.jval.2021.10.008.

2. Danese M. D., Pemberton-Ross P., Catterick D., Villa G. Estimation of the increased risk associated with recurrent events or polyvascular atherosclerotic cardiovascular disease in the United Kingdom. Eur J Prev Cardiol. 2021;28(3):335-343. https://doi.org/ 10.1177/2047487319899212.

3. Dreyer R. P., Zheng X., Xu X., et al. Sex differences in health outcomes at one year following acute myocardial infarction: A report from the China Patient-Centered Evaluative Assessment of Cardiac Events prospective acute myocardial infarction study. Eur Heart J Acute Cardiovasc Care. 2019;8(3):273-282. https://doi.org/ 10.1177/2048872618803726.

4. Xi X., Wang X., Xie W., et al. Comparison of Evolocumab and Ezetimibe, Both Combined with Statin Therapy, for Patients with Recent Acute Coronary Syndrome: A Cost-Effectiveness Analysis from the Chinese Healthcare Perspective. Cardiovasc Drugs Ther. 2022. https://doi.org/ 10.1007/s10557-021-07276-x.

5. Xie W., Song Y., Qin X., Jin P. Cost-Effectiveness of Evolocumab in Adult Patients with Atherosclerotic Cardiovascular Disease from Chinese Healthcare Perspective. Adv Ther. 2023;40(2):489-503. https://doi.org/ 10.1007/s12325-022-02372-2.

6. Chen Y., Yuan Z., Lu J., et al. Randomized study of evolocumab in patients with type 2 diabetes and dyslipidaemia on background statin: Pre-specified analysis of the Chinese population from the BERSON clinical trial. Diabetes Obes Metab. 2019;21(6):1464-1473. https://doi.org/ 10.1111/dom.13700.

7. China Cholesterol Education Program Working Committee, Atherosclerosis Thrombosis Prevention, Control Subcommittee of Chinese International Exchange, et al. China cholesterol education program (CCEP) expert advice for the management of dyslipidaemias to reduce cardiovascular risk (2019). Chin J Intern Med. 2020;59(1):18-22. https://doi.org/ 10.3760/cma.j.issn.0578-1426.2020.01.003.

8. Cardiology Atherosclerosis and Coronary Heart Disease Working Group of Chinese Society of. Chinese expert consensus on lipid management of very high⁃risk atherosclerotic cardiovascular disease patients. Chin J Cardiol. 2020;48(04):280-286. https://doi.org/ 10.3760/cma.j.cn112148‑20200121‑00036.

9. Sabatine M. S., Giugliano R. P., Keech A. C., et al. Evolocumab and Clinical Outcomes in Patients with Cardiovascular Disease. N Engl J Med. 2017;376(18):1713-1722. https://doi.org/ 10.1056/NEJMoa1615664.

10. Lorenzatti A. J., Eliaschewitz F. G., Chen Y., et al. Randomised study of evolocumab in patients with type 2 diabetes and dyslipidaemia on background statin: Primary results of the BERSON clinical trial. Diabetes Obes Metab. 2019;21(6):1455-1463. https://doi.org/ 10.1111/dom.13680.

11. Keech A. C., Oyama K., Sever P. S., et al. Efficacy and Safety of Long-Term Evolocumab Use Among Asian Subjects - A Subgroup Analysis of the Further Cardiovascular Outcomes Research With PCSK9 Inhibition in Subjects With Elevated Risk (FOURIER) Trial. Circ J. 2021;85(11):2063-2070. https://doi.org/ 10.1253/circj.CJ-20-1051.

12. Winning price for drugs. 2023. https://www.pharmcube.com/product/index. Accessed 03 Feb 2023.

13. Yin X., Huang L., Man X., et al. Inpatient Cost of Stroke in Beijing: A Descriptive Analysis. Neuroepidemiology. 2018;51(3-4):115-122. https://doi.org/ 10.1159/000491091.

14. Kong D., Liu X., Lian H., et al. Analysis of Hospital Charges of Inpatients with Acute Ischemic Stroke in Beijing, China, 2012-2015. Neuroepidemiology. 2018;50(1-2):63-73. https://doi.org/ 10.1159/000484212.

15. Yu Li. Analysis on Influencing Factors of Total Hospitalization Costs in Patients with Acute Myocardial Infarction. China Health Insur. 2022(04):45-47. https://doi.org/ 10.19546/j.issn.1674-3830.2022.4.009.

16. Wang P., Zhang B., Jin L., Liao H., Dong T. Association of various risk factors with prognosis and hospitalization cost in Chinese patients with acute myocardial infarction: A clinical analysis of 627 cases. Exp Ther Med. 2015;9(2):603-611. https://doi.org/ 10.3892/etm.2014.2087.

17. She R., Yan Z., Hao Y., et al. Health-related quality of life after first-ever acute ischemic stroke: associations with cardiovascular health metrics. Qual Life Res. 2021;30(10):2907-2917. https://doi.org/ 10.1007/s11136-021-02853-x.

18. Du Xudong Wang Ju, Meng Hongdao, Zhu Cairong, Zhu Ping, Li Mier. Health Utility of Patients with Stroke Measured by EQ-5D and SF-6D. J Sichuan Univ (Med Sci Edi). 2018;49(02):252-257. https://doi.org/ 10.13464/j.scuxbyxb.2018.02.020.

19. Wenbin DENG Qingwen，LIU. Analysis of health⁃related quality of life and influencing factors in patients with stroke. Journal of Nanjing Medical University（Social Sciences）. 2020;20(05):459-463.

20. Matza L. S., Stewart K. D., Gandra S. R., et al. Acute and chronic impact of cardiovascular events on health state utilities. BMC Health Serv Res. 2015;15:173. https://doi.org/ 10.1186/s12913-015-0772-9.

21. China National Health Commission of the People’s Republic of. China health statistics yearbook (2022). ed. 2022th, vol. Beijing: Peking Union Medical college Press; 2022.

22. Consumer price index: medical care, Annual average wage and Gross Domestic Product. 2023. http://www.gov.cn/shuju/index.html. Accessed 03 Feb 2023.
